# Diversity of Biological Effects Induced by Longwave UVA Rays (UVA1) in Reconstructed Skin

**DOI:** 10.1371/journal.pone.0105263

**Published:** 2014-08-20

**Authors:** Claire Marionnet, Cécile Pierrard, Christelle Golebiewski, Françoise Bernerd

**Affiliations:** L'Oréal Research and Innovation, Aulnay sous Bois, France; University of Tennessee, United States of America

## Abstract

Despite their preponderance amongst the ultraviolet (UV) range received on Earth, the biological impacts of longwave UVA1 rays (340–400 nm) upon human skin have not been investigated so thoroughly. Nevertheless, recent studies have proven their harmful effects and involvement in carcinogenesis and immunosuppression. In this work, an i*n vitro* reconstructed human skin model was used for exploring the effects of UVA1 at molecular, cellular and tissue levels. A biological impact of UVA1 throughout the whole reconstructed skin structure could be evidenced, from morphology to gene expression analysis. UVA1 induced immediate injuries such as generation of reactive oxygen species and thymine dimers DNA damage, accumulating preferentially in dermal fibroblasts and basal keratinocytes, followed by significant cellular alterations, such as fibroblast apoptosis and lipid peroxidation. The full genome transcriptomic study showed a clear UVA1 molecular signature with the modulation of expression of 461 and 480 genes in epidermal keratinocytes and dermal fibroblasts, respectively (fold change> = 1.5 and adjusted p value<0.001). Functional enrichment analysis using GO, KEGG pathways and bibliographic analysis revealed a real stress with up-regulation of genes encoding heat shock proteins or involved in oxidative stress response. UVA1 also affected a wide panel of pathways and functions including cancer, proliferation, apoptosis and development, extracellular matrix and metabolism of lipids and glucose. Strikingly, one quarter of modulated genes was related to innate immunity: genes involved in inflammation were strongly up-regulated while genes involved in antiviral defense were severely down-regulated. These transcriptomic data were confirmed in dose-response and time course experiments using quantitative PCR and protein quantification. Links between the evidenced UVA1-induced impacts and clinical consequences of UVA1 exposure such as photo-aging, photo-immunosuppression and cancer are discussed. These early molecular events support the contribution of UVA1 to long term harmful consequences of UV exposure and underline the need of an adequate UVA1 photoprotection.

## Introduction

Human skin is the largest body organ. At the interphase between the external environment and internal milieu, it has developed optimal mechanisms for sensing external factors, protecting, restoring and maintaining body homeostasis [1]. Solar ultraviolet (UV) rays constitute one of the external factors to which human skin is acutely as well as chronically exposed and can induce biological and clinical damage, such as sunburn, immunosuppression, photocarcinogenesis and photoaging. The complete range of UV rays is composed of UVC (100–290 nm), that are stopped by the ozone layer, UVB (290–320 nm) and UVA wavelengths (320–400 nm) that include UVA2 or shortwave UVA (320–340 nm) and UVA1 or longwave UVA (340–400 nm). UV rays that can reach the earth surface are a combination of UVB and UVA wavelengths. UVB are more energetic than UVA and their impact have been extensively studied and described. UVB can directly induce DNA damage, such as the mutagenic cyclobutane pyrimidine dimers and 6–4 photoproducts. Only UVB are responsible for the production of vitamin D by direct conversion of 7-dehydrocholesterol into vitamin D3 and they can induce the well-known sunburn reaction. UVA rays are mostly responsible for the generation of reactive oxygen species (ROS) leading to oxidative stress. UVA can reach the deep dermis and induce dermal damage. In the long term UVA are mostly involved in skin photoaging. Both UVA and UVB have been shown to be responsible for pigmentation, photoimmunosuppression and photocarcinogenesis [2].

While UVB account for around 5% total UV energy received at ground level, long wavelength UVA1 are the main component of terrestrial UV radiation (around 75% of total energy received on earth) [3]. Due to their energetic properties, UVA1 are less impacted than UVB by geo-orbital and environmental parameters, such as latitude, time of the year, hour of the day, meteorological conditions and ozone layer thickness. The latitudinal bands of UVA distribution are wider than those of UVB leading to intense exposure conditions in equatorial and tropical regions including India, Central and South America and Africa. In addition, it is important to note that the intensity of UVA1 rays exhibits low variation with increased latitude, indicating that loads of UVA1 throughout the year outside of the tropics are lower but more uniform throughout the year [4]. Indeed, in more temperate latitudes UVA irradiance is less affected by seasons than UVB irradiance leading in mid-winter in North Europe to erythemal exposure equally achieved by UVB and UVA [5], [6].

The high penetration properties of UVA1 also make that these wavelengths ranges deeply penetrate into the skin and reach the deep dermis, even through glass or cloudy sky. It has been estimated that 100 times more UVA than UVB photons reach the dermis [7].

In addition to solar exposure, UVA1 can be artificially emitted, during skin phototherapy treatments or during sunbed tanning sessions using devices emitting mainly UVA1.

Because of their lower energetic properties, the specific biological and clinical contributions of UVA1 to skin damage have long been underestimated and have not been investigated so thoroughly until recently. Today, an increasingly growing body of information converges to describe some harmful impacts of UVA1 on skin [5]. Studies carried out on mice showed that UVA1 were carcinogenic and responsible for photoaging signs such as dryness, skin thickening, leathery appearance, sagging and wrinkling [8], [9]. In humans, UVA1 exposure during phototherapy sessions leads to acute side effects including skin dryness, pruritus, polymorphic light eruptions and herpes simplex virus reactivation [10], [11]. A potential carcinogenic effect in humans can be strongly suspected since these wavelengths are able to induce DNA lesions and mutagenesis, and an increasing risk towards melanoma has also been associated with use of sunbeds [5], [12], [13]. In addition, UVA1 wavelength range, in contrast to UVA2, is highly immunosuppressive in human *in vivo* and is thought to be the largest contributor to immunosuppression resulting from incidental daily sun exposure [14], [15].

Considering the growing body of information that shows harmful impact of UVA1, it was important to increase knowledge of cellular, tissue and molecular effects of UVA1 on skin. *In vitro* reconstructed human skin, composed of both a living dermal equivalent and a fully differentiated epidermis, has been shown to be a useful tool for studying the cutaneous response towards UV exposure. The three dimensional architecture of the model allows us to take into account UV penetration properties depending on wavelength [16]. Using this model, we have previously reproduced the major epidermal alterations induced by UVB as well as identified direct dermal damage occurring after UVA (including UVA2 and UVA1) through ROS generation, dermal fibroblast apoptosis, matrix metalloproteinase (MMP) release and modulation of the expression of genes related to extracellular matrix homeostasis [17]–[19].

The present work aimed at exploring in depth the impact of UVA1 upon skin by combining the uses of this *in vitro* reconstructed human skin model and different morphological, biochemical and molecular techniques such as full genome transcriptomic analysis to depict, without any preconception, an exhaustive vision of the effects of UVA1 upon skin.

## Material and Methods

### Keratinocyte and fibroblast cultures

Normal human skin was obtained from surgical residues of breast reduction surgery, with the patients' written informed consent in accordance with the Helsinki Declaration and with Article L. 1243-4 of the French public Health Code. Patients' written informed consents were collected and kept by the surgeon. The samples were anonymized before their reception by the authors. Only age, sex and anatomical site of samples were specified to the authors. The authors did not participate in sample collection. Given its special nature, surgical residue is subject to specific legislation included in the French Code of Public Health (anonymity, gratuity, sanitary/safety rules…). This legislation does not require prior authorization by an ethics committee for sampling or use of surgical waste (http://www.ethique.sorbonne-paris-cite.fr/?q=node/1767). Normal epidermal human keratinocytes (NHK) were obtained and cultured as described by Rheinwald and Green on a feeder layer of Swiss 3T3 fibroblasts [20]. Human dermal fibroblasts were isolated from mammary skin explants.

### In vitro reconstructed skin [21]

Dermal equivalents were prepared as previously described using 7 ml of a mixture containing 10^6^ human dermal fibroblasts and 1.5 mg/ml native bovine type I collagen (Symatèse, France) in a 60 mm Petri dish. The dermal equivalents were allowed to contract for 4 days at 37°C, 5% CO_2_. Human epidermal keratinocytes grown in primary culture (33000/cm^2^) were seeded on this support using stainless rings. After 2 h rings were removed and the cultures were kept submerged for 7 days, allowing the cells to form a monolayer. The culture was then raised to the air-liquid interface on a grid and kept for 7 days. The medium was as previously described [22] and changed 3 times per week.

### Irradiation source and procedure

UVA1 spectrum was delivered by using a 1000 W Xenon lamp equipped with a dichroic mirror (Oriel, les Ulis, France) + WG360 2 mm thick filter (Schott, Clichy, France). The spectral irradiance was measured using a spectroradiometer (Macam Photometrics, Livingston, UK) (Figure S1). In order to deliver all of UVA1 wavelengths (up to 400 nm), a part of visible light spectrum (400–450 nm) could not be separated from applied UVA spectra of wavelengths. During UV exposure, the reconstructed skin medium was replaced by Dulbecco's phosphate-buffered saline (PBS) without calcium and magnesium (Gibco BRL). After UV exposure, PBS was removed and fresh medium was added. Reconstructed skin samples were then incubated at 37°C, 5% CO_2_ for different time periods depending on the performed analysis.

### Histology

48 hours after UVA1 exposure, samples were taken and fixed in neutral formalin. Paraffin sections were stained with haematoxylin, eosin, and saffron.

### Reactive Oxygen Species (ROS) assay

Reconstructed skins were incubated with 50 µM 2',7'-dichlorodihydrofluorescein diacetate (DCFH-DA, Invitrogen, Eugen, USA) for 30 min, at 37°C, 5% CO2. After PBS washing, samples were exposed or not to UVA1. Immediately after exposure, reconstructed skin samples were frozen in liquid nitrogen and 5 µm cryostat sections were made and fixed with acetone to allow the visualization of fluorescence generated by ROS in cells of the reconstructed skins. Green fluorescence was quantified in reconstructed skins using ImageJ software (http://rsb.info.nih.gov/ij/). The depth of immunostaining was measured as follows: green DCFH-DA positive cells were automatically detected using Histolab software and the distance between dermal epidermal junction and the deepest positive cells were measured in each condition. Means were compared using a Student's t test. Two means were considered statistically different when p<0.05.

### 8-Isoprostane detection

Determination of the amount of secreted 8-isoprostane in culture medium of UVA1 or sham-exposed reconstructed skin was performed 24 hours after UV exposure, using an enzyme immunoassay (Cayman Chemical, Ann Arbor, MI, USA), according to the manufacturer's instructions. The means of 8-isoprostane amounts were compared using a Student's t test. Mean of 8-isoprostane amounts were considered statistically different when p<0.05.

### Immunostaining

Immunostaining of vimentin was performed on air-dried vertical 5 µm cryosections using mouse monoclonal antibody against human vimentin (1∶20, Monosan, Unden, the Netherlands), as described [15]. Fluorescein isothiocyanate (FITC)-conjugate rabbit anti-mouse immunoglobulins (1∶80, Dako, Denmark), was used as a second antibody. Nuclear counterstaining using propidium iodide was carried out routinely.

### Assessment of secreted protein amount (ELISA)

The amounts of matrix metalloproteinases (MMP1, MMP3, MMP9) and cytokines IL-1β, IL-2, IL-4, IL-5, IL-6, IL-8, IL-10, IL-12 p70, TNFα, CSF2 ( = GM-CSF) were assessed in culture medium using Human MMP 3-Plex Ultra sensitive kit and Human Demonstration 10-Plex Tissue Culture Kit (MSD, Gaithersburg, MD, USA), respectively, according to manufacturer's instructions. The amount of CCL20, GDF15, HGF and CXCL10 were assessed using Human CCL20/MIP-3 alpha, human GDF-15, human HGF Quantikine and human CXCL10/IP10 ELISA Kits (R&D Systems Europe, Lille, France) respectively, according to manufacturer's instructions.

*Determination of significant modulations in protein amount*

For each tested protein and at each UVA1 dose, the mean protein amount of control samples was compared to the mean protein amount of UVA1 exposed samples using a Student's t test. Mean protein amounts were considered statistically different when p<0.05.

### Total RNA extraction

Reconstructed skin samples were rinsed in Dulbecco's PBS without calcium and magnesium (Gibco BRL). The epidermis was peeled off from dermal equivalent using fine forceps. Immediately after collection, epidermis and dermal equivalent were immersed separately in lysis buffer (Rneasy mini-kit, Qiagen). Epidermis disruption was performed by vortexing. The disruption of dermal equivalent was performed using a 2.5 mm stainless steel bead and a Mixer Mill MM300 (Retsch, Germany) for 2 minutes at 25 Hertz. The dermal equivalent lysate was submitted to Proteinase K (Qiagen) digestion (40 units, 20 min, 55°C).

The total RNA was obtained according to the manufacturer's instructions using Rneasy midi-kit (Qiagen). Dnase I treatment (27 units, 15 min) of total RNA was performed directly on the spin columns to eliminate genomic contamination of RNA samples. The quality, integrity and amount of total RNA were analyzed using a 2100 Bioanalyzer and an RNA NanoChip (Agilent Technologies, USA) and NanoDrop ND-1000 spectrophotometer (Thermo Fisher Scientific, USA), respectively. The average RIN of the total RNA samples was 9.5+/−0.4.

### Affymetrix microarray

#### Labeling and hybridization

For each sample, 300 ng of total RNA were amplified and labeled using the Affymetrix Whole-transcript (WT) Sense target Labeling Protocol without rRNA reduction (www.affymetrix.com). Ten µg of cRNA was carried out into the second cycle for first strand cDNA synthesis reaction. Affymetrix GeneChip Human Gene 1.0 ST arrays were hybridized with 5.5 µg of labeled sense DNA, washed and stained according to the Affymetrix GeneChip Expression Analysis Manual. Arrays were subsequently scanned on a GCS3000 7G Scanner. Data capture and initial array quality assessment were performed with the Affymetrix GeneChip Command Console v2.0 software.

#### Data analysis

Background correction and quantile normalization were performed for the raw microarray data using R and Bioconductor tools resulting in probe sets intensities for each GeneChip [23]. Hierarchical clusterings were generated using R software (www.r-project.org) and SpotFire Decision Site for Functional Genomics software.

#### Selection of modulated genes by UVA1 exposure in Affimetrix microarray experiments

The differential expression analysis between UVA1 exposed samples and control samples was performed using Limma package [24]. Pvalues were adjusted using the False Discovery Rate method [25]. For each probe set, the ratio of modulation induced by UVA1 was calculated as the ratio of mean mRNA amount in UVA1 exposed samples on mean mRNA amount in control samples. A probe set was considered to be modulated when: (i) at least a 1.5-fold modulation was induced by UVA1 (ratio >1.5 or <0.67) and (ii) mRNA amount in UVA1 exposed samples was statistically different from mRNA amount in control samples (Adjusted p (Adjp) value <0.001).

#### Functional enrichment analysis

The probe sets found significantly modulated by UVA1 in Affymetrix experiments (AdjP<0.001 and at least a 1.5-fold modulation) were subjected to functional enrichment analysis with both the Gene Ontology (GO) and Kyoto Encyclopedia of Genes and Genomes (KEGG) annotation databases. Over-representation of GO terms or KEGG pathways were tested using the GOstats Bioconductor package [26].

#### Bibliography study

The bibliographic study was performed for the genes that were found significantly modulated (AdjP<0.001) by at least 2 fold after UVA1 exposure, using Pubmed tools (http://www.ncbi.nlm.nih.gov/pubmed). For preferentially determining gene involvement in skin biology, the bibliographic research was performed using keywords related to skin. In case a gene or its corresponding protein was not described in skin biology, bibliographic study was enlarged without any key word. Genes of interest were then dispatched into functional groups.

### Quantitative reverse transcription-polymerase chain reaction (RT-PCR)

1 µg of total RNA was used for first strand cDNA synthesis using an Advantage RT-for-PCR kit (Clontech, Saint Quentin en Yvelines, France), according to the manufacturer's instructions.

Quantitative PCR (Q-PCR) was performed using the LightCycler and the LightCycler-FastStart DNA Master Sybr Green kit (Roche) as previously described [27]. Glyceraldehyde-3-phosphate-dehydrogenase (GAPDH), beta-2-microglobulin (B2M), ribosomal protein L13a (RPL13A), S28 (RPS28) and S9 (RPS9) mRNA were quantified with the LightCycler in each sample and used for normalization using Genorm application [28], [29]. Primer sequences are detailed in Table S1.

#### Determination of significant modulations in mRNA amount in Q-PCR assays

For each gene, each UVA1 dose and at each time point, to calculate whether the mean mRNA amount of control samples was statistically different from the mean mRNA amount of UVA1 exposed samples, means of the log of mRNA amount were compared using two-tailed Student's *t*-test (p<0.05).

## Results

### Cellular and tissue effects in reconstructed skin exposed to UVA1

**Figure 1:**
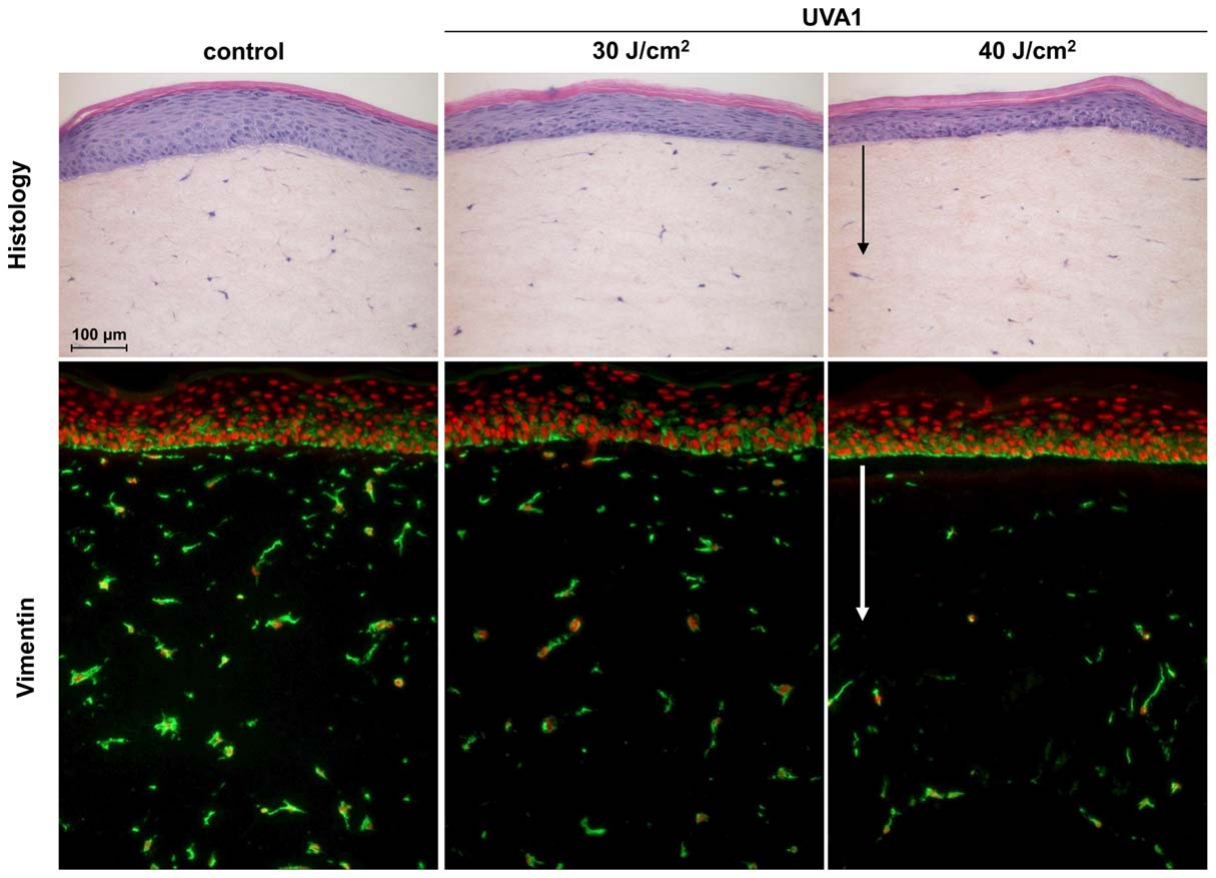
Cellular effects in human reconstructed skin exposed to UVA1. Sham-exposed (control) and UV-exposed samples were taken for classical histology and for vimentin staining (vimentin: green labeling, nuclei counterstaining: red labeling) at 48 h post UVA1 exposure. Arrows indicate fibroblast disappearance in human dermal equivalent.

To appreciate the impact of UVA1 wavelengths upon human reconstructed skin and based upon our previous studies on UVA exposure, we determined the UVA1 Biologically Efficient Dose (BED), *i.e.* the minimal dose necessary to induce morphological changes without destroying the tissue using histology and vimentin immunostaining to assess the viability of dermal fibroblasts and epidermal keratinocytes [16] (Figure 1). Control reconstructed skins exhibited a fully differentiated epidermis with well-structured horny layers onto a dermal equivalent showing fibroblasts embedded in a collagen matrix. The UVA1 BED, found to be 40 J/cm^2^, led to alteration of dermal superficial fibroblasts that disappeared 48 hours after UV exposure (Figure 1). Terminal deoxynucleotidyl transferase (TdT)-mediated dUTP nick end labeling (TUNEL) assay revealed that UVA1 induced fibroblasts death by induction of apoptosis: six hours after exposure to UVA1 BED, most of the fibroblasts of reconstructed skin were TUNEL positive (Figure S2). The epidermis was also, but to a lesser extent, affected by UVA1 exposure with alterations of granular layers, internalization of loricrin protein and in some cases the appearance of parakeratosis (Figure S3). Basal and suprabasal keratinocytes showed no histological modifications. Induction of apoptosis was not detected in epidermis of reconstructed skin exposed to UVA1 (Figure S2). To evaluate the biological contribution of UVA1 in the total UVA spectrum, UVA1 effects were compared with those induced by the whole UVA spectrum (including UVA2+UVA1) (Figure S4). We showed that effects of UVA1 and total UVA spectra were qualitatively similar, at quite equivalent doses (40 J/cm^2^ UVA1 and 35–40J/cm^2^ total UVA), with at these doses the clear disappearance of superficial dermal fibroblasts 48 h post exposure (compare Figure 1 and Figure S4). Altogether, these results show that UVA1 wavelengths have morphological and cellular effects on reconstructed skin and exhibit a significant contribution to the impact of the total UVA spectrum.

### Oxidative stress and DNA damage induced by UVA1 exposure in reconstructed skin

**Figure 2:**
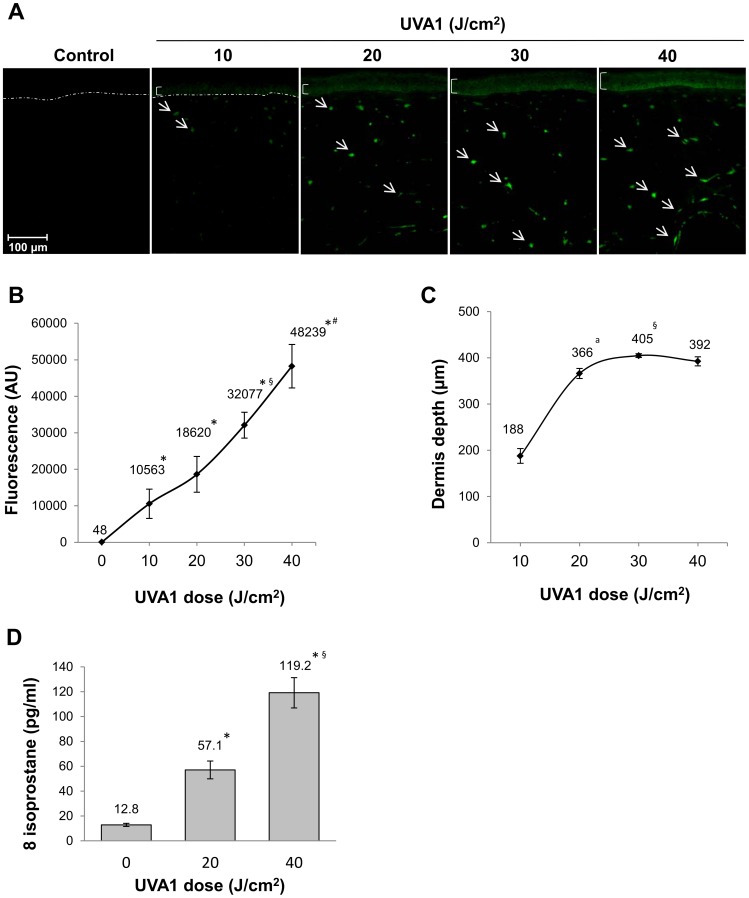
ROS and lipid peroxidation detection in reconstructed skin exposed to UVA1. ROS assay was performed using sections of reconstructed skin after DCFH-DA probe incorporation and UVA1. Bracket and arrows indicated the fluorescent keratinocytes and fibroblasts, respectively, in UVA1-exposed samples. None of them were detected in unexposed reconstructed skin. The dotted line indicates dermal epidermal junction (**A**). Levels of DCFH-DA probe fluorescence in reconstructed skin after UVA1 exposure. AU, arbitrary units (**B**). Distance between dermal epidermal junction and the deepest positive DCFH-DA cells. Indicated values correspond to the mean of 6 measurements in each experimental condition (**C**). 8-isoprostane amount in culture medium of reconstructed skin 24 hours after UVA1 exposure (**D**). ^*^, mean value significantly different from mean value at 0 J/cm^2^; ^a^, mean value significantly different from mean value at 10 J/cm^2^; ^§^, mean value significantly different from mean value at 20 J/cm^2^; ^#^, mean value significantly different from mean value at 30 J/cm^2^ (p<0.05, Student's t test).

The induction of ROS by raising doses of UVA1 (10 J/cm^2^–40 J/cm^2^) was visualized and quantified after incorporation of a DCFH-DA probe. UVA1 exposure led to a dose-dependent significant increase in ROS production in both dermal fibroblasts and epidermal keratinocytes starting at a dose as low as 10 J/cm^2^ UVA1 (Figures 2A and 2B). At this dose ROS were detected up to 188 µm deep in dermis and reached the deepest cells of the dermis at 30 and 40 J/cm^2^ UVA1, at around 400 µm deep in dermis (Figures 2A and 2C). The arachidonic acid derivative 8-isoprostane has been established as a marker for lipid peroxidation. A significant and dose-dependent increase in 8-isoprostane was found in the culture medium of reconstructed skin exposed to UVA1, reaching a nine-fold increase for the 40J/cm^2^ UVA1 dose, as compared to unexposed samples (Figure 2D).

Cyclobutane pyrimidine dimer (CPD) detection was performed by immunostaining 1 hour after exposure of reconstructed skins to UVA1, using a monoclonal anti-thymine dimer antibody. UVA1 exposed skin samples exhibited a low but clear staining in nuclei of basal keratinocytes, while the positive control sample exposed to UVB showed a strong staining in keratinocyte nuclei throughout the reconstructed epidermis (Figure S5).

### Analysis of gene expression using Affymetrix microarrays

To describe an overall view of molecular early events occurring after UVA1 exposure, a transcriptomic study was performed using a whole-transcript Affymetrix array. Three control reconstructed skins were sham-exposed (control) and 3 reconstructed skins were exposed to the UVA1 BED (40 J/cm^2^). Six hours following exposure, for each reconstructed skin, dermis was separated from epidermis and total RNA was extracted from dermal fibroblasts and epidermal keratinocytes, separately. For these 12 samples, the expression of about 20,000 genes was studied using the 1.0ST Affymetrix microarray. Very stringent quality controls at the different steps of the protocol (total RNA integrity, reverse transcription rate, amplification, cRNA labeling efficiency, hybridization on the chips, quality of the probe sets), did not lead to the exclusion of any sample.

### Overall gene expression analysis (figure 3)

**Figure 3:**
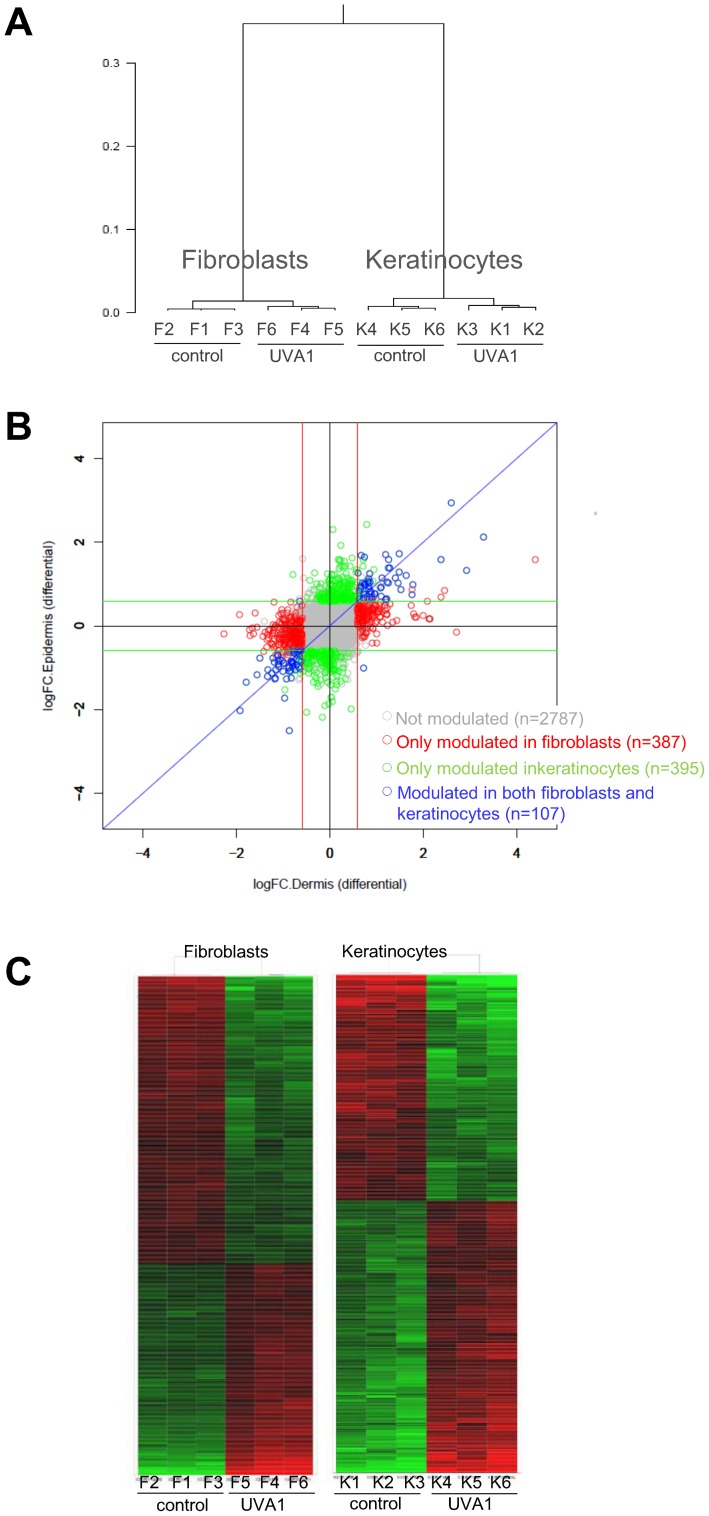
Overall analysis of gene expression in reconstructed skin exposed to UVA1, using microarray. Triplicates of reconstructed skins were unexposed (control) or exposed to 40 J/cm^2^ UVA1. Six hours later, a full genome transcriptomic study was conducted using Affymetrix microarray in fibroblasts (F) and keratinocytes (K), separately, for the 3 control reconstructed skins (samples F1-F3 and K1-K3) and for the 3 UVA1 exposed reconstructed skins (samples F4-F6 and K4-K6). **A**: Hierarchical clustering based on all probe set normalized expression data, using Ward's method and correlation distance. Y-axis of dendrogram represents the linkage distance that separates singletons or clusters. Height at which two clusters are merged in dendrogram reflects distance of the two clusters. **B**: Fold change comparison plot between fibroblasts and keratinocytes depicting number of significantly modulated probe sets 6 hours after exposure to 40J/cm^2^ UVA1. Each probe set is plotted. On y-axis: log of fold change value in keratinocytes; on x-axis: log of fold change value in fibroblasts. In keratinocytes and in fibroblasts, 502 and 494 probe sets were differentially expressed between UVA1 exposed and control reconstructed skins (fold-change >1.5 or <0.67, Adjp<0.001), respectively. Blue circles (n = 107) represent probe sets modulated in both keratinocytes and fibroblasts. Green (n = 395) and red (n = 387) circles represent probe sets modulated only in keratinocytes and in fibroblasts, respectively. Grey circles (n = 2787): un-modulated probe sets. **C**: Heat map showing relative expression levels of the probe sets differentially expressed between UVA1-exposed and control samples (fold-change threshold >1.5 or <0.67 and Adjp value<0.001). Two-dimensional hierarchical clustering was carried out with the 502 and 494 probe sets differentially expressed between UVA1 exposed and control samples in keratinocytes and fibroblasts, respectively. Euclidean distance and Ward's method, based on normalized log2-transformed gene expression value relative to median value of each row were used [85]. Each row represents a probe set, each column represents one sample. Red, high expression. Black, median expression. Green, low expression.

The visualization of the 12 gene expression profiles was performed using hierarchical clustering based on all probe set normalized expression data (Figure 3A). First, the generated dendrogram showed that triplicates of each experimental condition were very close to each other, attesting the reproducibility of the assay. Second, it underlined that fibroblast and keratinocyte gene expression was markedly different as attested by the length of the vertical lines of the dendrogram (linkage distance) between fibroblast and keratinocytes clusters. Third, it showed that clusters of control and of UVA1 samples were clearly separated. This revealed that exposure to 40 J/cm^2^ UVA1 can alter gene expression in both fibroblasts and keratinocytes of reconstructed skin.

In order to determine the number and nature of modulated genes by UVA1 in each cell type, 2 criteria were used: fold change value above 1.5 (up-regulation by UVA1) or below 0.67 (down-regulation by UVA1) and Adjp value<0.001.

According to these filters, 494 probe sets representative of 461 genes were modulated by UVA1 in fibroblasts and 502 probe sets representative of 480 genes in keratinocytes. One hundred and seven probe sets, representing less than 22% of the modulated probe sets, were commonly modulated by UVA1 in fibroblasts and in keratinocytes (Figure 3B). These results underline that dermal fibroblasts and epidermal keratinocytes exhibited a strong and specific response to UVA1 exposure.

The 494 and 502 UVA1- modulated probe sets in fibroblasts and keratinocytes respectively were used to perform a two-dimensional hierarchical clustering (Figure 3C). This representation allowed us to visualize up and down-regulations induced by UVA1. In fibroblasts UVA1 gene modulations were distributed as follows: 58% (285/494) of up-regulations and 42% (209/494) of down-regulations. In keratinocytes this distribution was inverted with 45% (228/502) of down-regulations and 55% (274/502) of up-regulations.

### Distribution of UVA1 modulated genes in functional families and pathways

In order to determine which functions and pathways were affected, the 494 and 502 probe sets found modulated by UVA1 in fibroblasts and in keratinocytes separately were classified using a functional enrichment analysis performed with GO and KEGG annotation databases. In fibroblasts and in keratinocytes, the most significant GO terms were related to response to stimulus, signaling and cell communication (up-regulated genes) and response to virus (down-regulated genes) (Table 1). In fibroblasts, GO terms related to cell death and apoptosis were also strongly over-represented for up-regulated genes, as well as GO terms related to development and morphogenesis and to migration (down-regulated genes) (Table 1 and Table S2). In keratinocytes specifically, GO terms related to biosynthesis and metabolism of glucose, protein or phosphate were significantly overrepresented among up-regulated genes; while for down-regulated genes, the most over-represented GO terms were related to lipid metabolism (Table 1 and Table S3). KEGG enrichment analysis in fibroblasts indicated modulated pathways involved in immunity (including for example Toll-like receptor signaling pathway), cancer, cardiomyopathy, adhesion, MAPK and Notch signaling pathways, glutathione metabolism and Extra Cellular Matrix (ECM)-receptor interaction (Table S4). In keratinocytes over represented KEGG pathways were related to immune response and related signaling pathways (such as cytokine-cytokine receptor interaction, and Jak STAT signaling pathway or RIG-I-like receptor signaling pathway) and to metabolism pathways (lipid, glucose, glutathione and nitrogen) (Table S5). Results of GO and KEGG analysis were consistent and attested that reconstructed skin exposure to UVA1 acted as a stress with cell response to stimulus, involving numerous cytokines, growth factors and transcription factors and revealed a disturbance of metabolism especially in keratinocytes.

**Table 1:** Summary of most significant enriched GO Biological Process terms in reconstructed skins exposed to UVA1.

	Number of GO terms	Pvalue min	Pvalue max
**Up-regulated probe sets in fibroblasts**			
Response to stimulus	19	2.4e-12	2.7e-07
Cell death/Apoptosis	11	5.9e-12	2.5e-07
Signaling	12	3.9e-10	2.9e-07
Protein modification	3	1.3e-08	1.7e-07
Cell communication	2	9.8e-08	2.7e-07
Regulation of metabolic process/biological process	4	1.0e-07	1.8e-07
**Down-regulated probe sets in fibroblasts**			
Response to virus	5	8.1e-12	3.2e-11
Response to cytokine/Innate immunity	4	9.8e-08	5.6e-07
Development/Morphogenesis	28	3.3e-09	1.3e-05
Cell migration/Motility	6	3.2e-08	2.9e-06
Signaling	2	7.6e-07	2.2e-06
Response to stimulus	5	7.8e-07	4.4e-06
**Up-regulated probe sets in keratinocytes**			
Response to stimulus	21	2.3e-11	7.8e-06
Signaling	13	4.6e-09	4.2e-06
Cell communication	3	3.8e-08	1.1e-06
Cell death/Apoptosis	4	6.0e-07	6.7e-06
Glucose metabolism	3	3.2e-07	3.8e-06
Phosphate metabolism	2	1.9e-06	3.6e-06
Protein metabolism	2	3.4e-06	7.4e-06
Development	1	9.2e-07	9.2e-07
Oxidative stress response	1	5.2e-06	5.2e-06
**Down-regulated probe sets in keratinocytes**			
Lipid metabolism	24	1.9e-06	0.0033
Response to virus	13	4.9e-06	0.0034
Metabolic process	6	0.00012	0.0014
Cell migration	2	0.0028	0.0028
Response to stress	2	0.0028	0.0033
Miscellanous	3	0.00017	0.0024

Summary of the list of the top 50 enriched GO terms related to Biological Process (BP) for the up-regulated probe sets and down-regulated probe sets in fibroblasts and keratinocytes of reconstructed skins exposed to UVA1. The detailed lists are given in Tables S2 and S3, for fibroblasts and keratinocytes, respectively.

Although exploiting GO and KEGG databases with a large number of genes gave an informative overall and very general view of involved pathways in response to UVA1, these tools were not found completely satisfying since i) databases include information that does not focus on skin topics and are mainly driven by cancer data and ii) all of the modulated probe sets were not classified in GO and KEGG enrichment analysis.

**Figure 4:**
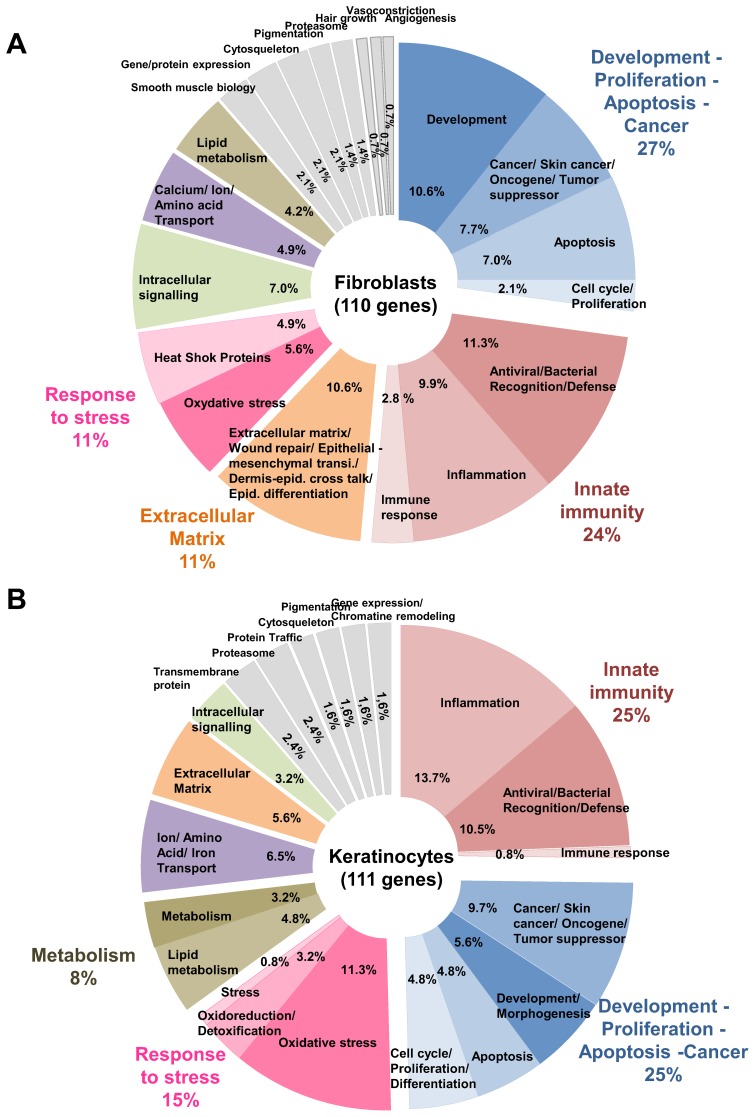
Distribution of UVA1 modulated genes in functional families. In order to perform an exhaustive bibliographic analysis including literature related to skin and dermatology, the list of UVA1 modulated genes was reduced by using a fold change threshold>2 or <0.5, and an Adjp<0.001. Under these criteria, 134 and 141 genes were found modulated in fibroblasts and keratinocytes respectively of UVA1 exposed reconstructed skins. In fibroblasts 24 genes out of 134 could not be classified because their functions were poorly described; 110 genes were distributed in functional families (**A**). In keratinocytes 30/141 genes could not be classified; 111 genes were distributed in functional families (**B**). Some genes could be classified in several functional families. Lists of gene names associated with gene bank accession number, fold change values and their distribution in functional families are given in Tables S6 and S7, for fibroblasts and keratinocytes respectively.

To provide a more detailed and specific biological information a manual bibliographic analysis based upon Pubmed literature focusing on skin biology was performed on UVA1 modulated genes from a restricted list that was established using a fold change threshold>2 or <0.5 and an Adjp<0.001. Under these criteria, 134 and 141 genes were found modulated in fibroblasts and keratinocytes of UVA1 exposed reconstructed skins, respectively. Gene distributions in functional families are given in Figure 4 and detailed in Tables S6 and S7. Although most UVA1 modulated genes differ between fibroblasts and keratinocytes (Figure 3B), this classification revealed that both cell types implemented responses involving common functional processes. For instance, in fibroblasts and keratinocytes, UVA1 affected the expression of genes classified in the following families: Development, Cell cycle/proliferation, Apoptosis Cancer, Innate immunity Extracellular matrix, Response to oxidative stress, Ion/amino acid/calcium/iron transport, Lipid metabolism, and Intracellular signaling (Figure 4).

One quarter of the responses to UVA1 included genes encoding proteins involved in innate immunity (24% and 25% in fibroblasts and keratinocytes respectively). In this functional family, two main biological processes were found, i.e. Inflammation and Antiviral or antibacterial recognition and defense. Interestingly, the Inflammation family was mostly composed of genes encoding pro-inflammatory markers that were all up-regulated following UVA1 exposure, in fibroblasts as well as in keratinocytes (Tables S6 and S7). The highest intensities of up-regulation were found in this Inflammation family. On the other hand, the Antiviral or antibacterial recognition and defense family, included interferon inducible genes and genes encoding double stranded RNA or C-type lectin-like receptors, that were all down-regulated after UVA1 exposure, in both fibroblast and in keratinocytes (Tables S6 and S7).

Another quarter of UVA1 modulated and classified genes encoded proteins involved in processes related to cell homeostasis, such as development, apoptosis, cell cycle and cancer, (Figure 4). In fibroblasts, genes involved in development represented 11% of the modulated and classified genes and were mostly down-regulated. On the other hand, most of the apoptosis markers (7% and 5% of the modulated and classified genes in fibroblasts and keratinocytes respectively), were up-regulated (Tables S6 and S7). Genes related to cancer, proliferation and cell cycle represented 10 and 14.5% of the modulated and classified genes in fibroblast and keratinocytes, respectively.

In fibroblasts and in keratinocytes, genes involved in response to oxidative stress represented 6 and 11% of the UVA1 modulated and classified genes, respectively. They were mostly up-regulated and many of them belong to the Nrf2 inducible pathway (Figure 4, Tables S6 and S7). In fibroblast the Response to stress family also included genes encoding heat shock proteins that were all up-regulated (5% of modulated genes) following UVA1 exposure. In keratinocytes the gene encoding ATF3 transcription factor, was included in the sub family “stress” of the Response to stress family and was up-regulated.

### Analysis of UVA1 dose and time effect on gene expression

In order to validate microarray data and to specify UVA1-gene modulation profiling of markers representative of main functional families, quantitative PCR experiments at different time points (2 h, 6 h and 24 h post exposure) and at different doses (20 and 40 J/cm^2^) were performed in fibroblasts and keratinocytes of reconstructed skins (Tables 2 and 3, respectively).

**Table 2:** Gene expression modulation assessed by quantitative PCR in fibroblasts of reconstructed skins exposed to UVA1.

	20 J/cm^2^			40 J/cm^2^		
	2 h	6 h	24 h	2 h	6 h	24 h
**Inflammation**						
CSF2	**2.8**	2.6	1.4	**5.1**	**18.5**	9.8
IL1A	2.3	1.0	2.7	3.2	**1.9**	3.0
IL1B	1.4	1.5	2.6	**2.0**	2.6	**6.9**
IL6	**4.7**	3.7	0.9	**4.3**	**7.4**	**2.4**
IL8	**5.0**	**6.1**	0.6	1.4	**23.1**	2.4
PTGS2	14.1	1.1	0.9	**37.5**	**6.9**	**4.4**
CCL20	1.1	**3.1**	0.9	1.1	**6.0**	**4.7**
LIF	1.2	1.0	0.8	2.3	4.2	**6.8**
TNFAIP3	1.1	**1.8**	1.0	**2.1**	**2.8**	**1.8**
ICAM1	1.0	**2.4**	1.3	0.9	**3.4**	**1.9**
**Anti Viral/Bacterial Recognition/Defense**						
SAMD9	0.7	0.8	1.1	0.7	**0.4**	0.8
SAMD9L	0.8	**0.4**	0.8	0.8	**0.3**	**0.3**
IFIT1	0.7	**0.4**	1.0	0.9	**0.1**	**0.5**
IFIT2	**0.3**	**0.6**	0.9	0.4	**0.3**	**0.4**
IFIT3	**0.5**	**0.5**	**3.3**	**0.4**	**0.2**	**1.8**
MX1	0.5	0.6	1.1	0.7	**0.2**	0.7
MX2	0.7	0.5	0.8	0.9	**0.1**	**0.3**
OAS1	0.5	0.7	**4.0**	0.8	**0.4**	2.0
OAS2	0.6	**0.4**	1.0	1.0	**0.2**	0.7
GBP1	0.9	**0.6**	0.8	1.1	**0.3**	**0.2**
GBP2	0.7	0.7	0.9	0.8	**0.4**	**0.3**
GBP5	0.6	**0.5**	0.9	0.6	**0.3**	**0.6**
GBP6*	0.8	1.4	2.1	0.9	0.6	0.6
TLR3	0.6	**0.5**	1.3	0.7	**0.3**	0.6
DDX58	0.7	**0.5**	0.8	0.7	**0.3**	**0.7**
CLEC2A	0.6	0.8	0.8	0.6	**0.5**	**0.2**
**Oncogene/Tumor suppressor/Cancer**						
JUN	1.0	1.0	1.1	**2.2**	**3.8**	1.0
**Development**						
APCDD1	**0.4**	**0.4**	**0.5**	**0.5**	**0.3**	**0.1**
**Proliferation**						
IGF1	**0.6**	**0.5**	**0.4**	0.7	**0.2**	**0.1**
**Apoptosis**						
DDIT3	**4.8**	2.2	0.6	**7.7**	**4.0**	1.5
NR4A1	**3.3**	0.7	0.5	**5.5**	2.9	0.8
IER3	**1.7**	1.1	1.1	2.3	**3.2**	**2.3**
**Response to stress**						
DNAJB1	1.2	1.2	1.4	**2.0**	**5.3**	**3.1**
HSPA1A	1.2	1.0	1.1	**2.6**	**6.3**	**2.4**
HSPA6	1.0	**5.5**	**6.9**	**4.2**	**183.4**	**198.4**
ATF3*	**4.6**	1.7	1.1	**13.2**	**10.7**	**6.0**
**Response to oxidative stress**						
HMOX1	**7.4**	**3.9**	0.6	**7.3**	**11.2**	1.3
TXNRD1	1.1	**2.7**	1.0	**2.3**	**5.8**	**2.8**
NQO1	1.3	1.3	1.1	0.8	**1.9**	1.5
SLC7A11	1.8	**5.6**	2.7	2.1	**11.3**	**3.3**
TXNIP	**0.6**	**0.7**	**0.5**	**0.4**	**0.3**	**0.3**
**Extracellular matrix**						
COL1A1	**0.5**	**0.6**	**0.4**	0.7	**0.4**	**0.2**
MMP1	0.9	**1.8**	**2.9**	0.8	**2.3**	**10.6**
MMP3*	0.8	0.7	**2.1**	0.9	1.1	**9.5**
SERPINB2	1.8	1.5	**1.8**	1.4	1.7	**8.2**
GDF15	4.7	**3.2**	**3.8**	**6.1**	**8.0**	**7.9**
**Intracellular signaling**						
GEM	1.4	**1.7**	ND	**2.9**	**3.1**	**1.6**

Reconstructed skins were exposed to 20 or 40 J/cm^2^ UVA1 and recovered 2, 6 or 24 hours later. Expression of genes found modulated in the microarray study and/or representative of main functional families was analyzed by quantitative PCR in fibroblasts of reconstructed skin. Ratios of modulation induced by UVA1 exposure were calculated for each condition as the ratio of mean mRNA amount in UVA1 exposed samples to mean mRNA amount in sham exposed control samples (columns 2 to 7). Bold text indicate mean ratio values with significant differences between UVA1 exposed and control samples (p <0.05, Student's t test). All studied genes, except those underlined and those marked with an asterisk, were found modulated by UVA1 in microarray experiments, in fibroblasts. Underlined genes were not found modulated in microarray experiments but were of interest regarding UVA stress and photoageing. Asterisks indicate genes found modulated in keratinocytes but not in fibroblasts, in microarray experiments. ND, not detected.

**Table 3:** Gene expression modulation assessed by quantitative PCR in keratinocytes of reconstructed skins exposed to UVA1.

	20 J/cm^2^			40 J/cm^2^		
	2 h	6 h	24 h	2 h	6 h	24 h
**Inflammation**						
CSF2	1.8	1.8	**0.5**	**5.3**	**19.1**	1.1
IL1A	2.0	**1.8**	1.0	**3.3**	**5.5**	1.4
IL1B	1.2	1.6	0.8	**1.9**	**2.2**	1.5
IL6	2.4	3.7	0.4	**3.1**	**6.0**	0.5
IL8	0.8	**7.4**	0.5	**2.9**	**52.1**	4.3
PTGS2	**4.7**	**2.0**	0.6	**17.4**	**9.5**	0.9
CCL20	**2.5**	**3.5**	1.2	**13.2**	**66.2**	**2.9**
LIF	ND	ND	ND	ND	ND	ND
TNFAIP3	**1.6**	**1.8**	0.8	**3.1**	**12.2**	**1.8**
ICAM1	1.8	**1.5**	0.8	2.5	**2.8**	1.0
**Anti Viral/Bacterial Recognition/Defense**						
SAMD9	0.9	0.7	0.8	0.7	**0.5**	**0.6**
SAMD9L	2.2	3.5	3.8	2.4	**1.1**	**2.5**
IFIT1	0.7	**0.4**	0.9	**0.7**	**0.1**	0.7
IFIT2	**0.4**	0.7	1.0	**0.2**	**0.1**	**0.5**
IFIT3	0.6	0.8	1.1	**0.5**	**0.2**	1.0
MX1	0.6	1.4	1.0	**0.4**	**0.2**	**4.2**
MX2	**0.5**	**0.5**	1.1	**0.6**	**0.2**	1.3
OAS1*	0.9	0.8	1.2	1.0	**0.4**	1.3
OAS2*	0.9	0.7	0.8	1.5	**0.2**	**0.5**
GBP1*	1.1	0.8	0.4	1.2	**0.2**	0.4
GBP2	0.8	1.0	0.7	0.8	**0.6**	0.1
GBP5^§^	1.0	0.9	0.9	0.8	**0.4**	1.0
GBP6	0.7	0.8	0.8	0.6	**0.3**	**0.3**
TLR3	0.7	0.6	1.0	0.8	**0.2**	0.7
DDX58*	0.9	1.2	1.0	1.8	2.3	0.9
CLEC2A	0.9	**0.5**	**0.8**	**0.6**	**0.3**	**0.2**
**Oncogene/Tumor suppressor/Cancer**						
JUN*	1.3	1.7	0.7	**2.5**	2.1	0.9
ODC1	**1.5**	**2.0**	1.0	**2.3**	**3.7**	**1.8**
FOSB*	**6.9**	**2.1**	0.9	**20.4**	**19.1**	**1.8**
CTSL1	1.1	**1.8**	1.0	**1.5**	**3.4**	1.3
CTSH	0.9	0.7	0.7	**0.7**	**0.4**	**0.3**
**Development**						
APCDD1	0.7	**0.6**	**0.6**	0.7	**0.3**	**0.3**
BMP2	**2.2**	**1.6**	0,6	**4.4**	**7.1**	1.1
OSR2	**0.8**	**0.6**	0,9	**0.5**	**0.3**	**0.4**
**Proliferation**						
IGF1*	ND	ND	ND	ND	ND	ND
**Apoptosis**						
DDIT3	**6,3**	**2,5**	1,5	**13,2**	**6,1**	0,8
NR4A1*	**12.7**	**4.5**	0.6	**14.1**	**19.5**	1.7
IER3	1.1	1.3	**0.8**	1.7	**2.5**	1.1
**Epidermal differentiation/proliferation**						
KRT10	1.1	1.0	0.7	1.1	0.7	**0.3**
KRT2	**0.6**	0.7	**0.4**	0.7	0.5	**0.3**
SERPINB2	1.0	1.2	0.8	**2.0**	1.1	1.3
TGM1	0.8	**1.8**	0.9	0.8	**2.0**	1.0
**Response to Stress**						
DNAJB1*	0.9	1.3	1.2	1.6	1.4	1.1
HSPA1A*	1.0	1.3	0.9	1.3	1.4	1.0
HSPA6*	1.0	1.7	**0.7**	**1.8**	**9.1**	**2.4**
ATF3	**18.6**	**8.9**	**0.6**	**45.7**	**52.9**	**2.0**
**Response to oxidative stress**						
HMOX1*	1.1	1.1	0.9	1.3	1.2	0.8
TXNRD1	1.8	**1.9**	1.4	**5.0**	**2.9**	1.4
NQO1	**2.2**	**3.0**	3.1	**2.6**	**4.1**	4.8
SLC7A11	**2.0**	**7.4**	1.8	3.3	**4.6**	**0.3**
TXNIP	**0.4**	**0.2**	**0.6**	**0.4**	**0.2**	**0.1**
**Extracellular matrix**						
MMP3	1.3	1.1	1.1	**2.4**	**3.0**	1.0
GDF15	**1.7**	**9.8**	17.5	**4.7**	**21.8**	30.2
**Intracellular signaling**						
GEM	**3.8**	**3.2**	0.6	**5.3**	**12.0**	**1.8**

Reconstructed skins were exposed to 20 or 40 J/cm^2^ UVA1 and recovered 2, 6 or 24 hours later. Expression of genes found modulated in the microarray study and/or representative of main functional families was analyzed by quantitative PCR in keratinocytes of reconstructed skin. Ratios of modulation induced by UVA1 exposure were calculated for each condition as the ratio of mean mRNA amount in UVA1 exposed samples to mean mRNA amount in sham exposed control samples (columns 2 to 7). Bold text indicate mean ratio values with significant differences between UVA1 exposed and control samples (p <0.05, Student's t test). All studied genes, except those underlined and those marked with an asterisk were found modulated by UVA1 in microarray experiments, in keratinocytes. Underlined genes were not found modulated in microarray experiments but were of interest regarding UVA stress, photoageing and keratinocyte biology. Asterisks indicate genes found modulated in fibroblasts but not in keratinocytes, in microarray experiments. ND, not detected.

We particularly focused on genes related to innate immunity (inflammation and antibacterial/antiviral defense), cancer, development, proliferation and apoptosis; as well as genes related to stress and oxidative stress response, extracellular matrix and epidermal differentiation- proliferation balance and intracellular signaling. In keratinocytes and in fibroblasts, UVA1 induced gene modulations found using microarrays were confirmed using quantitative PCR. Moreover a clear dose-response was underlined for most of the studied markers (Tables 2 and 3).

Genes encoding pro-inflammatory markers were strikingly found up-regulated in both cell types as early as 2 hours after UVA1 exposure. On the contrary genes encoding proteins involved in antiviral and antibacterial recognition and defense were strongly down-regulated, particularly at 6 hours following UVA1 exposure.

Genes encoding proteins related to apoptosis such as DDIT3, NR4A1 and IER3 were up-regulated by UVA1 exposure in both cell lines, whereas the IGF1 proliferation marker and the APCDD1 development-related gene were down-regulated (in fibroblasts and both cell types respectively).

The response to oxidative stress was characterized by an induction of expression of Nrf2 target genes (HMOX1 in fibroblasts, TXNRD1 and NQO1 in both cell types), of SLC7A11 and the down-regulation of TXNIP (inhibitor of thioredoxin), in both fibroblasts and keratinocytes.

The epidermal proliferation/differentiation balance was also affected by UVA1 exposure with the down-regulation of K2 and K10 gene expression at 24 hours post exposure and the induction of SERPINB2 and TGM1 gene expression.

UVA1 also changed the expression of genes encoding proteins involved in extracellular matrix composition and remodeling, such as COL1A1 gene expression that was down-regulated, and MMP1, MMP3, SERPINB2 or GDF15 genes whose expression was induced in fibroblasts of UVA1 exposed samples. These regulations peaked at 24 hours following UVA1 exposure. In keratinocytes MMP3 and GDF15 were also induced by UVA1 exposure.

The cell line specificity of response to UVA1 revealed in microarray data was assessed using quantitative PCR performed in both cell types on several specific genes (GBP6, ATF3 and MMP3 specific for keratinocytes in microarray experiments; OAS1, OAS2, GBP1, GBP5, DDX58, JUN, IGF1, NAR4A1, DNAJB1, HSPA1A, HSPA6, HMOX1 and LIF specific for fibroblasts in microarray experiments). Q-PCR experiments confirmed that GBP6 was only modulated in keratinocytes and not in fibroblasts as well as MMP3 which was down regulated in keratinocytes at time points 2 and 6 hours while in fibroblasts it was 24 hours after UVA1 exposure. Q-PCR experiments also confirmed that DDX58, IGF1, DNAJB1, HSPA1A HMOX1 and LIF genes were specific for the response of fibroblast to UVA1 exposure, since they were not modulated at any time point and at any UVA1 dose or not detected in keratinocytes (Table 3). The modulation of JUN was found in keratinocytes but at an earlier time point (2 h).

The specificity of ATF3 modulation by UVA1 in keratinocytes in microarray experiments was not confirmed in Q-PCR experiments, since a strong and significant up-regulation was also found in fibroblasts. However, it should be noted that in microarray data the 1.9-fold induction of ATF3 in fibroblasts was not considered significant (AdjPvalue  = 0.0017) under our statistical criteria (AdjP value <0.001). Q-PCR experiments revealed that OAS1, OAS2, GBP1, NR4A1, and HSPA6 were modulated at 6 hours after UVA1 exposure in both fibroblasts (Table 2) and keratinocytes (Table 3). Again the specificity of their modulation in fibroblasts underlined in the microarray data was the consequence of our stringent statistical criteria, since all of these genes showed absolute fold modulation above 1.4 but AdjP values comprised between 0.003 and 0.02 in microarray experiments. GBP5 gene showed no significant modulation 6 hours following UVA1 in keratinocytes in microarray experiments (down-regulation of 1.15 compared to control and AdjP value  = 0.315) but was found significantly down-regulated in these cells in Q-PCR results (fold change = 0.4, *i.e.* down-regulation of 2.5 compared to control and pValue<0.05). Therefore, with the exception of the marker GBP5, for all tested genes, Q-PCR results confirmed those from microarray's, *i.e.* changes by UVA1 of the expression of genes re1ated to important functional families and a cell response specificity for the genes GBP6 (only modulated in keratinocytes), DDX58, IGF1, DNAJB1, HSPA1A, HMOX1 and LIF (only modulated in fibroblasts).

### Effect of UVA1 on the level of proteins secreted by reconstructed skins

**Figure 5:**
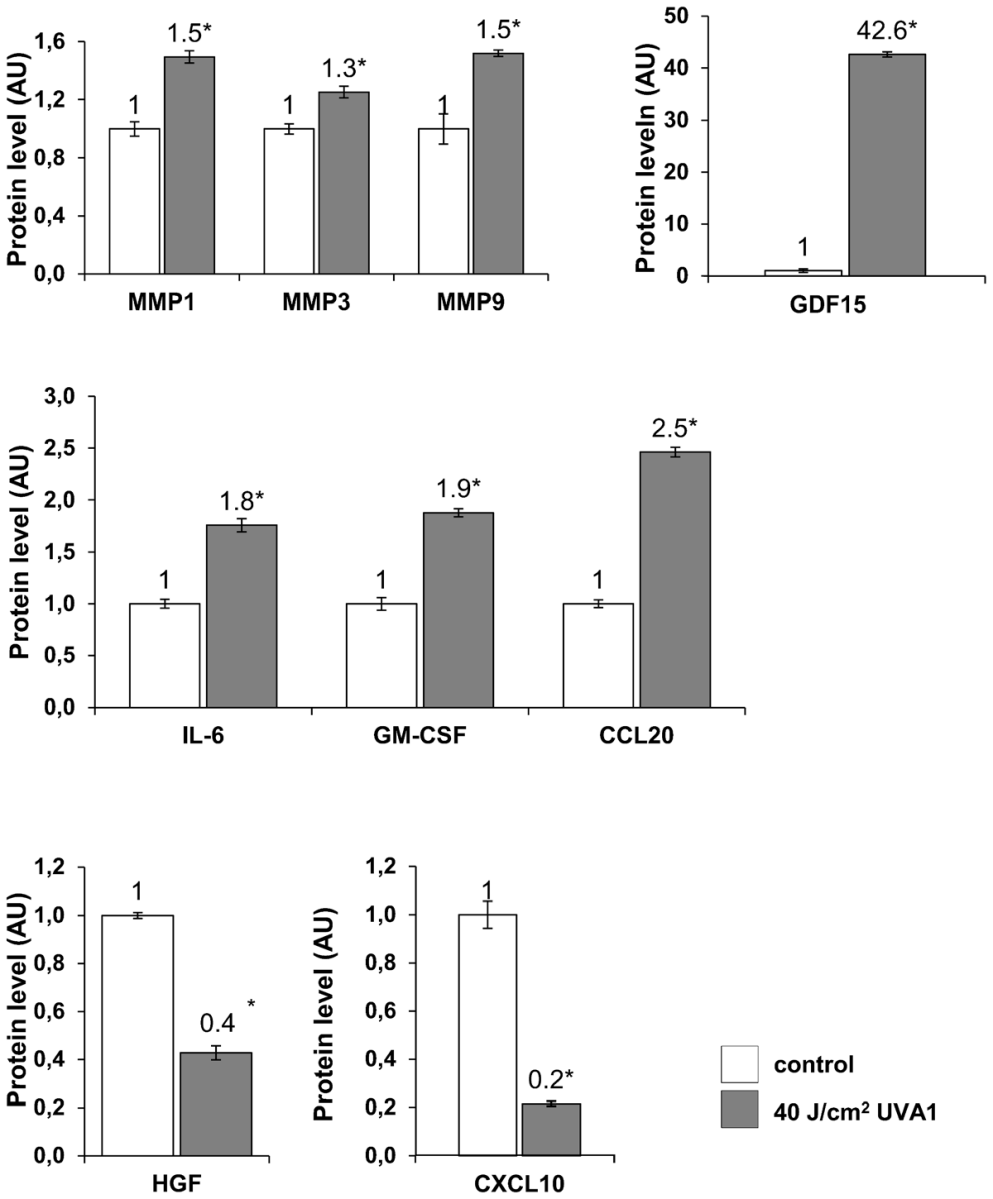
Levels of secreted proteins in culture medium of reconstructed skin exposed to UVA1. Culture media were taken at 48 hours post UVA1 exposure and used to measure the amount of extracellular matrix remodeling proteins (matrix metalloproteinases, MMPs and GDF15), pro-inflammatory proteins (IL-6, GM-CSF/CSF2, CCL20 and GDF15), the HGF growth factor and the CXCL10 ( = IP10) interferon inducible protein. Values of control samples were adjusted to the 1 value. Asterisks indicate a significant difference between mean protein amount of control samples and mean protein amount of UVA1 exposed samples (p<0.05, Student's t test). AU, arbitrary units.

The amount of proteins encoded by some of genes whose expression was affected by UVA1 exposure was quantified in the culture medium 48 hours after exposure to 40 J/cm^2^ UVA1 (Figure 5). Proteins involved in ECM degradation and remodelling (MMP1, MMP3, MMP9 and GDF15) and in skin inflammation (IL-6, CSF2 ( = GM-CSF), CCL20 and GDF15) were shown to be increased by UVA1 exposure. The amount of HGF protein, involved in cell growth, was decreased, as well as the amount of CXCL10 protein (also named interferon gamma inducible protein, IP10), encoded by an interferon inducible gene. The modulations of these protein amounts by UVA1 correlated with the gene expression data.

## Discussion

This study aimed at characterizing the biological impacts of UVA1 upon skin cells, using a reconstructed skin model that shares similar properties with human skin, such as epidermal differentiation and 3-dimensional structure, allowing us to particularly appreciate the impact of UVA1 from surface to depth. To our knowledge, only one study had previously studied the modulation of few endpoints after UVA1 exposure (Sellamed 3000 source) of living skin equivalents (morphology, TUNEL, IL1, HO-1 and 8 oxo G) [30]. We are mostly in agreement with their results but our study offers a much wider view of UVA1 impact on reconstructed human skin using a more relevant UVA1 source.

The biological efficient UVA1 dose in our biological system was 40 J/cm^2^ corresponding to a physiological dose that could be received in a few hours [31]. In our experiments this dose was the maximal dose used in this study and we showed that many of the biological changes induced by UVA1 occurred at doses below 40 J/cm^2^.

### 1-Immediate injury induced by UVA 1: oxidative stress and DNA damage

First of all, UVA1 immediately induced the production of reactive oxygen species (ROS), in a dose-dependent manner in both fibroblasts and keratinocytes of reconstructed skin, even after a dose as low as 10 J/cm^2^ of UVA1. Increasing doses of UVA1 induced ROS deeply in the epidermal basal layer and in the dermal equivalent, with ROS detected in the deepest dermal fibroblasts (400 µm) with the doses of 30 and 40 J/cm^2^. This suggests that higher doses could generate ROS deeper than 400 µm; a probable case in human skin where dermis can reach 1 to 3 mm thickness. These results particularly emphasized the deep impact of UVA1 wavelengths in line with data obtained using physical approaches showing the deep penetration of long UV wavelengths [32]. One important ROS-induced pathway is the cell membrane damage by lipid peroxidation. Twenty-four hours post UVA1 exposure a strong increase in stable end product formed by free radical-catalysed peroxidation of arachidonic acid, 8-isoprostane, was observed. Evidence of UVA1 induced lipid peroxidation has also been reported *in vivo* [33]. In addition, 8-isoprostane may also mediate other biological responses involved in vascular, proinflammatory and nociceptive processes.

Our experiments confirmed that UVA1 induced detectable thymine dimers in basal keratinocytes of reconstructed skin, in agreement with recent *in vivo* data showing the generation of CPD especially in basal keratinocytes of human skin [34]. Together with oxidative DNA damage such as 8 oxo-guanine, UVA1 induced DNA damage have been shown to be mutagenic *in vitro* and *in vivo* and UVA–induced pyrimidine dimers would be more mutagenic than those induced by UVB [12], [35]–[37]. Interestingly, UVA1 induced DNA mutations were also preferentially formed in the basal layer of human epidermis attesting a particular vulnerability of this epidermal layer, location of epidermal stem cells, proliferative keratinocytes and melanocytes [38], [39].

### 2- A global stress response to UVA1 exposure with major morphological alterations in the dermal compartment

#### Fibroblast apoptosis

Following UVA1 immediate injury, a biological impact throughout the whole skin structure could be evidenced, from morphology to gene expression analysis. The morphological effects induced by UVA1 BED dose confirmed that UVA1 *per se* contribute to the biological effects of UVA exposure, particularly in the dermis with a clear disappearance of dermal superficial fibroblasts, reinforcing the significant biological impact of UVA1 in deeper layers of the reconstructed skin. It appeared that UVA1 cytotoxicity towards fibroblast observed in our skin model was mostly due to apoptosis detected as early as 6 hours after UVA1 exposure. Interestingly, epidermal keratinocytes showed no apoptotic process, although DNA damage and ROS accumulation could be detected, showing a higher susceptibility of fibroblast toward UVA1 cytotoxicity, in agreement with previous *in vitro* data [40]–[42]. UVA1 induced apoptosis of dermal fibroblasts was correlated with the up-regulation of genes related to cell death and apoptosis, as illustrated in GO enrichment analysis and bibliographic study. Among these genes, early inducible genes such as DDIT3, IER3, BIRC3 and three members of the nuclear receptor subfamily 4, group A (NR4A1, -A2, -A3) were significantly up-regulated 2 to 6 hours post exposure to 20 and/or 40 J/cm^2^ UVA1. Although the precise mechanisms are still unknown, Breuckmann *et al*, suggested different apoptotic mechanisms of action between UVB and UVA1 in human T cells: for UVA1 an immediate initiation of apoptosis (6 hours after UV exposure) followed by early membrane rupture, while for UVB a delayed apoptosis (24 hours after UVB exposure) [43].

#### Stress response

Transcriptomic analysis showed that UVA1 induced DNA damage and ROS generation were followed by a response to stress. In GO enrichment analysis the terms “response to a stimulus” were among the most significant, in fibroblasts and keratinocytes of reconstructed skin exposed to UVA1. Bibliographic analysis confirmed that 11% (in fibroblasts) and 15% (in keratinocytes) of the modulated and classified genes belonged to the stress response family.

Particularly, the reconstructed skin cells exhibited a defense response to oxidative stress, with the up-regulation of the expression of Nrf2 pathway genes such as HMOX1, TXNRD1, NQO1, FTL, GCLM, AKR1C2 and AKR1C3, two to six hours following the generation of ROS by UVA1. This expression of antioxidant response genes would be induced by UVA1 mediated lipid oxidation [33], [44]. Interestingly, apart from the protection from ROS cell damage, it was recently shown *in vivo* that activation of the Nrf2 pathway in keratinocytes caused corneocyte fragility, alterations of the epidermal lipid barrier, inflammation and overexpression of mitogens inducing keratinocytes proliferation [45].

Stress response was also attested by the strong up-regulation of the expression of genes encoding heat shock proteins (HSP) such as DNAJB1, DNAJB9 (HSP40 family) HSPA1A ( = HSP72), HSPA1B, HSPA6 (HSP70 family), HSPB8 and HMOX1 especially in fibroblasts of reconstructed skin six hours after UVA1 exposure. Heat shock proteins are chaperone molecules whose expression is induced in order to respond to sudden environmental changes. HSP 70 and HSP72 are induced in keratinocytes after UVB exposure [46]–[48]. It has been shown that HMOX1 and HSP72 gene expression is induced after UVA exposure [49] [50]. Our present study shows that in addition to HMOX1 and HSP72, UVA1 can modulate the expression of several members of HSP70 and HSP40 families as well as HSPB8 that could be part of a natural defense mechanism against UV [51].

#### Cell type specificity of response

The transcriptomic study confirmed that UVA1 can alter the epidermis as well as the dermis in the depth with similar numbers of modulated genes in keratinocytes and fibroblasts (480 and 461 respectively). Our microarray experiments also evidenced that fibroblasts and keratinocytes exhibited specific responses, with less than 22% of the modulated probe sets commonly modulated by UVA1 in both cell types. This cell type specificity was confirmed for several markers using Q-PCR: GBP6 was only modulated in keratinocytes; DDX58, IGF1, DNAJB1, HSPA1A, HMOX1 and LIF were only modulated in fibroblasts. This specificity of response could be in part explained by the fact that fibroblast and keratinocytes have a different basal gene expression as illustrated in Figure 3A. This could be also due to the location of the skin cells in the 3D model where dermal fibroblasts received the longest wavelengths whereas epidermal keratinocytes receive the whole UVA1 spectrum. This difference of gene response after UV exposure between fibroblasts and keratinocytes had already been observed in previous studies [52], [53].

### 3- Diversity of the biological response face to UVA1 exposure

Apart from modulated genes linked to stress response and apoptotic process, the transcriptomic study allowed us to establish a wide view of biological pathways and functions impacted by UVA1 exposure including innate immunity, extracellular matrix, development, metabolism and cancer. These early molecular events can be informative of consequences of such UVA1 exposure occurring in a long term process or after repetitive exposures.

#### Extra-cellular matrix

The expression of genes related to extracellular matrix composition and remodeling was modulated in reconstructed skin exposed to UVA1. For instance, members of the TGF pathway (including BMP2 and GDF15) were up-regulated while the growth factors FGF1, FIGF and HGF (gene and protein) were down-regulated in fibroblasts. Moreover in fibroblast, genes and proteins of matrix metalloproteases MMP1 and MMP3 were up-regulated, mostly 24 hours after UVA1, while COL1A1 gene was repressed. These results are in agreement with previous data showing that UVA induce dermal damage such as alterations of collagen and elastic fibers and MMP-1 expression [54] [55] [56], [57]. These alterations can be correlated with *in vivo* clinical signs of photoaging due to chronic exposure to UVA. This is particularly well illustrated by cases of unilateral dermatoheliosis occurring on site of the face chronically exposed to UVA through a glass window (*e.g.* truck drivers) and showing striking skin thickening, roughness, wrinkling and laxity associated with an accumulation of elastotic material within dermis [58]. In addition, changes in fibroblast homeostasis and microenvironment can promote tumor progression [59].

#### Metabolism

Apart from classical photoaging related genes, UVA1 exposure also altered the expression of genes related to lipid metabolism in keratinocytes and fibroblasts, (5 and 4% respectively), such as CH25H (cholesterol 25-hydroxylase), ELOVL3 (elongation of very long chain fatty acids -like 3) and ACSS3 (acyl-CoA synthetase short-chain family member 3). Interestingly Kim *et al* recently showed an alteration of lipid metabolism in the epidermis during photoaging process and acute UV exposure, with decreased amount of free fatty acids and triglycerides [60]. In addition, a metabolomic study revealed an increased degradation of triglycerides in sun-exposed skin [61]. Since skin lipids mediate various skin physiological responses such as epidermal barrier homeostasis, epidermal proliferation, energy metabolism and MMP-1 increase, modulation of lipid metabolism by UVA1 may alter these functions [62] [63]. In addition to lipid metabolism, UVA1 also modulated genes related to glucose metabolism especially in epidermal keratinocytes, as revealed by GO analysis. For instance, three pyruvate deshydrogenase kinase genes (PDK1, PDK2, PDK3) were down-regulated by UVA1. Since these enzymes inhibit the conversion of pyruvate into lactate, exposure to UVA1 may induce this conversion, promoting energy production. ALDOC gene encoding a glycolytic enzyme involved in the balance glycolysis/gluconeogenesis and H6PD gene (hexose-6-phosphate dehydrogenase) were also down-regulated by UVA1 whereas PGM3, PYGB and UGDH were up-regulated. Altogether these results attest a marked disruption in glycolysis and glycogen degradation, pathways that have been considered as major contributors of energy production in skin. This alteration of glucose metabolism by acute UVA1 exposure could be correlated with the up-regulation of metabolites such as glucose, lactate and 3-phosphoglycerate in sun-exposed human skin [61].

#### Development, cancer and proliferation

In both cell types, one quarter of the UVA1-modulated and classified genes were related to cellular homeostasis including apoptosis, proliferation, development and cancer functional families. Enrichment of KEGG pathways, GO analysis and bibliographic study showed that UVA1 modulated the expression of genes involved in cancer, such as oncogenes that were up-regulated (FOS, FOSB, MAFG, ABL2, MET, ETS1), tumor suppressors that were down-regulated (FETB and RARRES1), cathepsin genes (CTSH, CTSL1) or PTGS2 gene that have a crucial role in the development of skin cancers [64] [65]. Most of these genes had been shown to be modulated by UV. We show here that UVA1 wavelengths *per se* modulate these markers. It may be hypothesized that up-regulation of an activated oncogene or down-regulation of a tumor suppressor by UVA1 would favor tumor development.

#### Immunity

One of the two major gene functional families affected by UVA1 exposure was innate immunity, in fibroblasts and in keratinocytes of reconstructed skin, illustrating the immune competence of these cutaneous cells [66].

The induction of inflammation by ultraviolet radiation, especially UVB, is well described [67] [68]. We previously showed that UVA spectrum, including UVA2 and UVA1, can induce proinflammatory mediators in reconstructed skin [19]. We now show that UVA1 exposure *per se* induced an increase in the level of inflammation markers, such as IL6, CCL20 and CSF2 (GM-CSF) genes and proteins as well as IL1A, IL1B, IL8, PTGS2 (COX-2), TNFAIP3, ICAM1 and LIF genes. These results are in agreement with previous data showing that UVA1 was responsible for the modulation of cytokines such as IL1 and IL6 over production in fibroblasts; leading to an increase in MMP1 [69]. The use of experimental filters with different absorption spectra proved the involvement of UVA1 wavelengths in IL1 and IL6 production [57].

Besides their proinflammatory potency, UV can induce immunosuppression. It was described that UVA1, as well as UVB, altered adaptive immunity by significantly reducing response to delayed-type hypersensitivity and to contact hypersensitivity in human [14], [70]. Due to the far greater proportion of UVA1 in solar UV, the relative solar immune suppressive efficiency of UVA1 was threefold higher than that of UVB at doses received during normal daily activities [15]. UVA1 induced alteration of adaptive immunity can involve cellular and molecular mechanisms such as isomerization of urocanic acid [71], morphological alteration and depletion of Langerhans cells *via* the generation of ROS and reactive nitrogen species [72]–[74] and reduction of calcineurin activity due to ROS [75].

Interestingly, our results showed that UVA1 could affect antiviral and antibacterial innate immunity with a strong down-regulation, 6 hours after UVA1 exposure, of numerous genes involved in antiviral and antibacterial defense, such as interferon (IFN) inducible genes (SAMD9, SAMD9L, IFIT1, IFIT2, IFIT3, MX1, MX2, OAS1, OAS2, GBP2, GBP5, GBP6, CXCL10…), as well as genes encoding receptors to double stranded RNA (TLR3, DDX58) and C type lectin receptors (CLEC2A, CLEC2B). In the absence of viral infection, cells constitutively produce very low levels of type 1 IFN [76]. In response to viral and other microbial infection, IFNα/β are massively produced and trigger the induction of interferon-inducible genes, downstream of the Jak-Stat or other IFN-regulated pathways [77]. This interferon response constitutes a strong barrier against viral multiplication in the infected host [78]. Loss of IFNα/β signaling in animal models usually leads to uncontrolled viral replication [79]. Apart from antiviral activity, the type 1 interferon response is also involved in tumor suppression [80]. We show here that in fibroblasts and keratinocytes of reconstructed skin, the constitutive IFNα/β signaling was strongly inhibited by UVA1 exposure. This UVA1 driven down-regulation may have deleterious consequences on antiviral/antibacterial and antitumoral defense. Although elicitation of IFN response is fully documented, down-regulation of IFN signaling has mostly been described in the case of virus for evading host immune response [81]. To our knowledge, only two papers reported down-regulation of IFN signaling after UV exposure. In a murine keratinocyte cell line stimulated by IFNγ, UVB down regulated IFN-signaling by interfering with phosphorylation of STAT-1 and IRF-1 binding. The authors stated that inhibition of IFN activity by UV light may contribute to its immunosuppressive activity [82], [83]. In addition, a recent study showed that narrowband UVB inhibits IFNα or INFγ induced expression of double stranded RNA receptors in human primary keratinocytes [84]. This down-regulation of expression of antiviral defense genes by UVA1 can be correlated with the reactivation of herpes simplex virus following UVA1 phototherapy in human, one of the most reported side effect [10], and more generally to reactivation of herpes simplex and herpes zoster viruses observed after the first sun exposure of summer.

Considering that human beings are significantly exposed to UVA1 rays all along their lives, implementing the characterization of their impacts upon human skin is a paramount objective. Using a reconstructed skin model we showed that UVA1 generated oxidative stress and DNA damage, stressing skin, from surface to depth, from tissue to molecular level, affecting a wide variety of cellular functions. Ultimately, the UVA1 induced damage evidenced in this study might be linked to clinical consequences such as photo-aging, photo-immunosuppression and cancer. This data, together with previous one recently published, highly plea for an adequate and efficient photoprotection in the UVA1 range.

## Supporting Information

Figure S1 UVA1 and total UVA (UVA2+UVA1) spectra. Spectra were delivered using a 1000 W Xenon lamp equipped with a dichroic mirror. WG360 2 mm or WG335 3 mm thick filter was added to deliver the UVA1 spectrum (340–450 nm) or the total UVA (UVA2+UVA1) spectrum (320–450 nm), respectively. In order to deliver all UVA1 wavelengths (up to 400 nm), a part of visible light, ranging from 400 to 450 nm, could not be avoided and was part of both UVA1 and total UVA spectra.
(PPTX)

Figure S2 TUNEL assay on reconstructed skin exposed to UVA1. Reconstructed skins were exposed to 40 J/cm^2^ UVA1 and TUNEL reaction was performed at 0 h, 1 h, 2 h, 3 h, 6 h and 24 h following UV exposure, as described [15] using the In Situ Cell Detection Kit (Roche Diagnostic, Germany) on 4% formaldehyde fixed frozen sections. Nuclear conterstaining using propidium iodide was carried out routinely (red signal). Some TUNEL positive fibroblasts (green signal, indicated by white arrows) were detected in dermal equivalent 3 hours after UVA1 exposure. Six hours after exposure, most fibroblasts were stained and the level of signal intensified at 24 hours.
(PPTX)

Figure S3 Epidermal alterations induced by UVA1. 48 hours after 40J/cm^2^ UVA1 exposure, reconstructed skins were taken for histology (haematoxylin, eosin, saffron) and loricrin immunostaining using a rabbit polyclonal antibody against loricrin (Dr Magnaldo; [86]) and FITC-conjugate swine anti rabbit immunoglobulin as second antibodies. Histology of UVA1 exposed samples revealed an alteration of granular layers, with a disappearance of keratohyalin granule and, in some cases, the appearance of parakeratosis (black arrows). The impact of UVA1 on granular layers was also evidenced by loricrin immunostaining. In non-exposed control samples, loricrin staining was in periphery of granular cells while UVA1 led to a subcellular redistribution of loricrin, leading to a wider cytoplamic localization (white arrows).
(PPTX)

Figure S4 Cellular effects in human reconstructed skin exposed to total UVA (UVA2+UVA1). Sham-exposed (control) and UV-exposed samples were taken for classical histology and for vimentin staining (vimentin: green labeling, nuclei counterstaining: red labeling) at 48 h post (UVA1+UVA2) exposure (see Figure S1 for UVA1+UVA2 spectrum). Arrows indicate fibroblast disappearance in human dermal equivalent. The BED of total UVA was found to be 35–40 J/cm^2^ (depending on experiments).
(PPTX)

Figure S5 Cyclobutane pyrimidine dimers (CPD) immunostaining in human reconstructed skin exposed to UVA1. Reconstructed skins were exposed to 40 J/cm^2^ UVA1 or to 382 mJ/cm^2^ UVB (positive control). Skin samples were harvested one hour after exposure in order to perform CPD immunostaining using a monoclonal anti-thymine dimer antibody (1∶1000, TDM2, CosmoBio, UK), a biotinylated goat anti-mouse secondary antibody (BA-9200, Vector Laboratories, UK), and Vectasein Elite ABC Kit for peroxidase detection (PK-6100, Vector Laboratories, UK). UVB-exposed reconstructed skins exhibited strong positive staining in nuclei of keratinocytes, throughout the epidermis. In UVA1 exposed reconstructed skin a lower but clear signal was detected in nuclei of basal keratinocytes compared to non exposed skin sample.
(PPTX)

Table S1 Primer sequences used in quantitative PCR experiments.
(DOCX)

Table S2 Most significant enriched GO terms Biological Process in fibroblasts of reconstructed skin exposed to UVA1. Detailed list of the top 50 enriched GO terms related to Biological Process (BP) for the up-regulated probe sets and down-regulated probe sets in fibroblasts of reconstructed skins exposed to UVA1. GOBPID: Gene ontology identity of enriched terms. Size: total number of probes on microarray belonging to specific GO identities. Count: number of differentially expressed probe sets on microarray belonging to specific GO identities.
(DOCX)

Table S3 Most significant enriched GO terms Biological Process in keratinocytes of reconstructed skin exposed to UVA1. Detailed list of the top 50 enriched GO terms related to Biological Process (BP) for the up-regulated probe sets and down-regulated probe sets in keratinocytes of reconstructed skins exposed to UVA1. GOBPID: Gene ontology identity of enriched terms. Size: total number of probes on microarray belonging to specific GO identities. Count: number of differentially expressed probe sets on microarray belonging to specific GO identities.
(DOCX)

Table S4 Enriched KEGG pathways for the 494 probe sets found modulated in fibroblasts of reconstructed skin exposed to UVA1. KEGGID: KEGG identity of enriched terms. Size: total number of probes on microarray belonging to specific KEGGID. Count: number of differentially expressed probe sets on microarray belonging to specific KEGGID.
(DOCX)

Table S5 Enriched KEGG pathways for the 502 probe sets found modulated in keratinocytes of reconstructed skin exposed to UVA1. KEGGID: KEGG identity of enriched terms. Size: total number of probes on microarray belonging to specific KEGGID. Count: number of differentially expressed probe sets on microarray belonging to specific KEGGID.
(DOCX)

Table S6 Restricted list of the 134 genes modulated by UVA1 in fibroblasts of reconstructed skins, in Affimetrix microarrays. Selection criteria of the restricted list of modulated genes were as follows: fold change modulation threshold >2 or <0.5, and the Adjp value <0.001. Ratio values <1 were transformed as -1/ratio value, so that positive and negative values denote up-regulations (red) and down-regulations (green), respectively. Twenty-six genes were classified into two or three functional families. They were marked with an asterisk. The section “Other” includes 23 genes that could not be classified in functional families, because their functions were not enough described or determined.
(DOCX)

Table S7 Restricted list of the 141 genes modulated by UVA1 in keratinocytes of reconstructed skins, in Affimetrix microarrays. Selection criteria of the restricted list of modulated genes were as follows: fold change modulation threshold >2 or <0.5, and the Adjp value <0.001. Ratio values <1 were transformed as -1/ratio value, so that positive and negative values denote up-regulations (red) and down-regulations (green), respectively. Eleven genes were classified into two or three functional families. They were marked with an asterisk. The section “Other” includes 30 genes that could not be classified in functional families, because their functions were not enough described or determined.
(DOCX)
