# Supplementary material for: Diversity of Biological Effects Induced by Longwave UVA Rays (UVA1) in Reconstructed Skin
Source: PLoS One. 2014 Aug 20;9(8):e105263. doi: 10.1371/journal.pone.0105263 (PMC4139344; doi:10.1371/journal.pone.0105263)
Supplement: Figure S1 — UVA1 and total UVA (UVA2+UVA1) spectra. Spectra were delivered using a 1000 W Xenon lamp equipped with a dichroic mirror. WG360 2 mm or WG335 3 mm thick filter was added to deliver the UVA1 spectrum (340–450 nm) or the total UVA (UVA2+UVA1) spectrum (320–450 nm), respectively. In order to deliver all UVA1 wavelengths (up to 400 nm), a part of visible light, ranging from 400 to 450 nm, could not be avoided and was part of both UVA1 and total UVA spectra. (PPTX) [file pone.0105263.s001.pptx]

## Slide 1
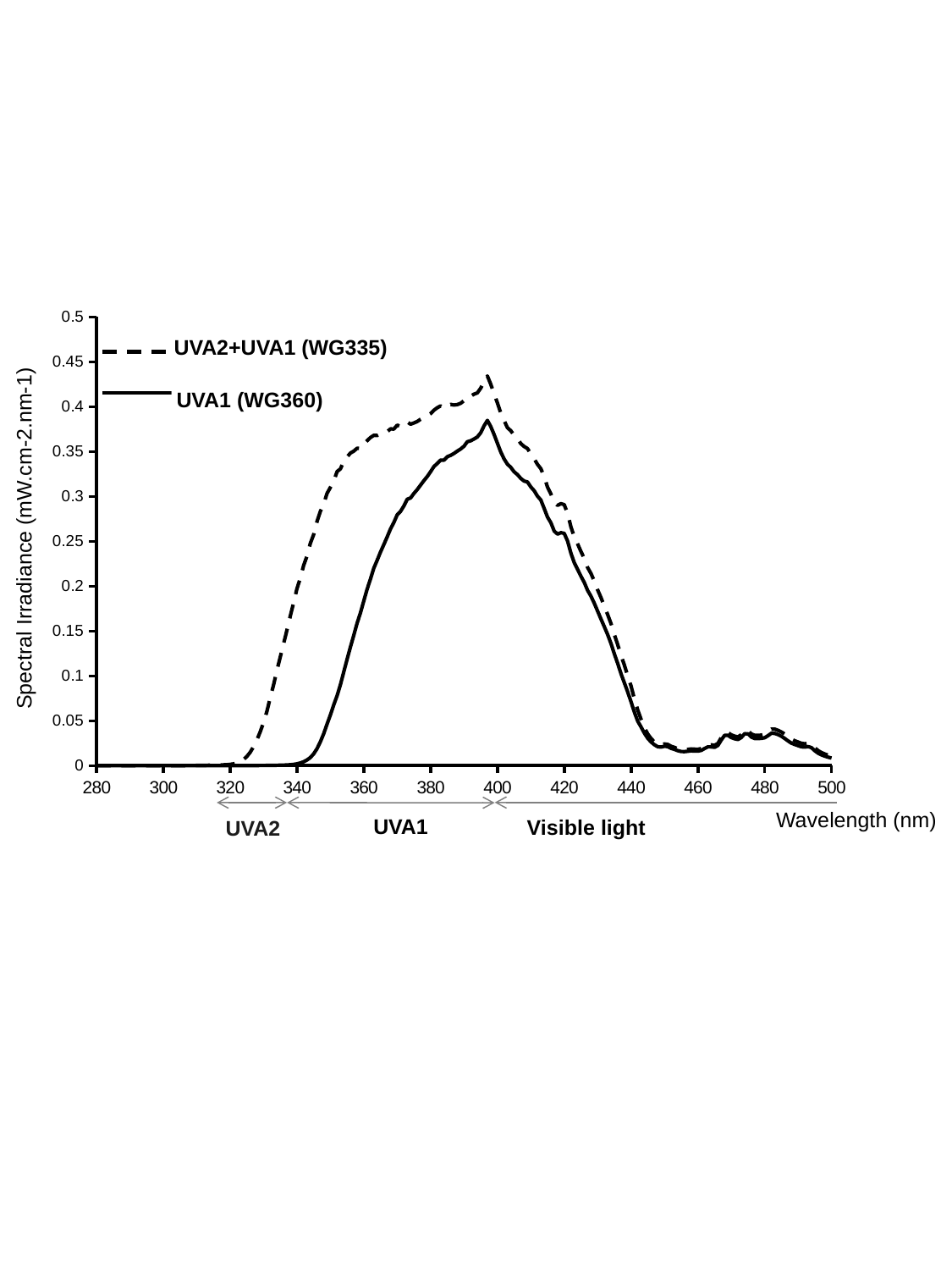

### Chart
| Category | | UVA-I (WG360) |
|---|---|---|UVA2+UVA1 (WG335)
UVA1 (WG360)
Spectral Irradiance (mW.cm-2.nm-1)
Wavelength (nm)
UVA1
Visible light
UVA2
