# Supplementary figures and images for: Diversity of Biological Effects Induced by Longwave UVA Rays (UVA1) in Reconstructed Skin
Source: PLoS One. 2014 Aug 20;9(8):e105263. doi: 10.1371/journal.pone.0105263 (PMC4139344; doi:10.1371/journal.pone.0105263)

## Slide 1
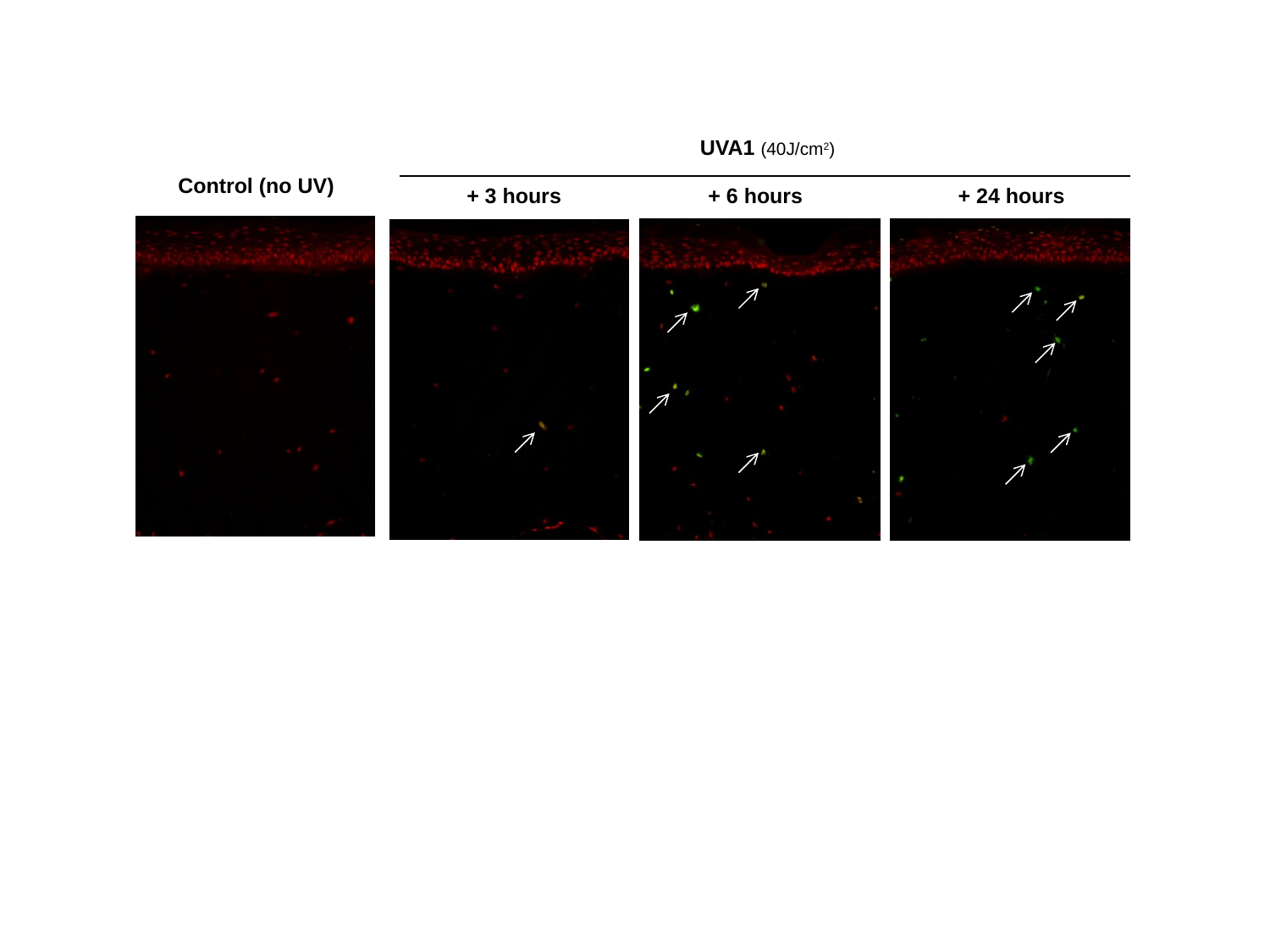

UVA1 (40J/cm2)
Control (no UV)
+ 3 hours
+ 6 hours
+ 24 hours

Supplement: Figure S2 — TUNEL assay on reconstructed skin exposed to UVA1. Reconstructed skins were exposed to 40 J/cm2 UVA1 and TUNEL reaction was performed at 0 h, 1 h, 2 h, 3 h, 6 h and 24 h following UV exposure, as described [15] using the In Situ Cell Detection Kit (Roche Diagnostic, Germany) on 4% formaldehyde fixed frozen sections. Nuclear conterstaining using propidium iodide was carried out routinely (red signal). Some TUNEL positive fibroblasts (green signal, indicated by white arrows) were detected in dermal equivalent 3 hours after UVA1 exposure. Six hours after exposure, most fibroblasts were stained and the level of signal intensified at 24 hours. (PPTX) [file pone.0105263.s002.pptx]

## Slide 1
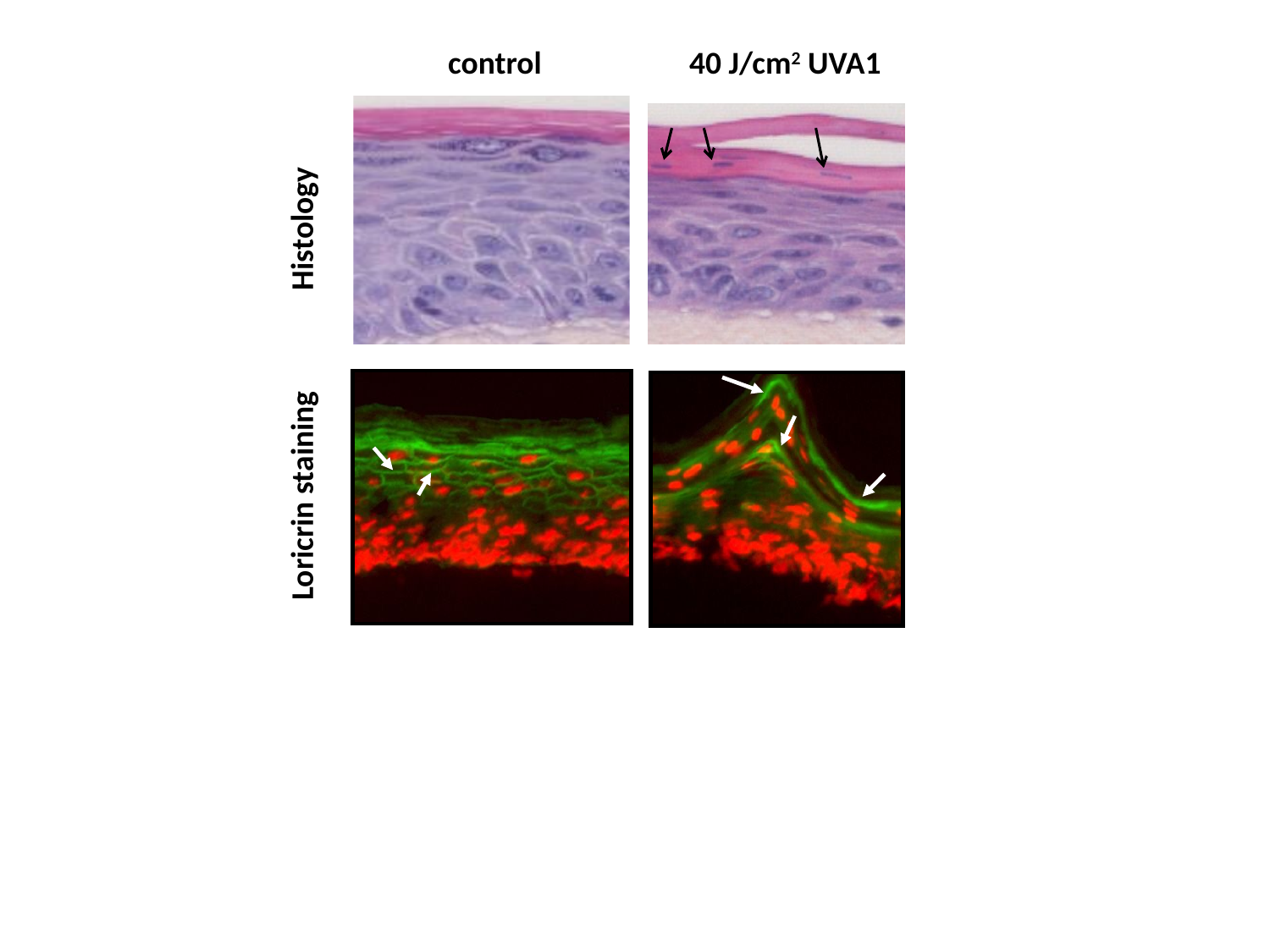

control
40 J/cm2 UVA1
Histology
Loricrin staining

Supplement: Figure S3 — Epidermal alterations induced by UVA1. 48 hours after 40J/cm2 UVA1 exposure, reconstructed skins were taken for histology (haematoxylin, eosin, saffron) and loricrin immunostaining using a rabbit polyclonal antibody against loricrin (Dr Magnaldo; [86]) and FITC-conjugate swine anti rabbit immunoglobulin as second antibodies. Histology of UVA1 exposed samples revealed an alteration of granular layers, with a disappearance of keratohyalin granule and, in some cases, the appearance of parakeratosis (black arrows). The impact of UVA1 on granular layers was also evidenced by loricrin immunostaining. In non-exposed control samples, loricrin staining was in periphery of granular cells while UVA1 led to a subcellular redistribution of loricrin, leading to a wider cytoplamic localization (white arrows). (PPTX) [file pone.0105263.s003.pptx]

## Slide 1
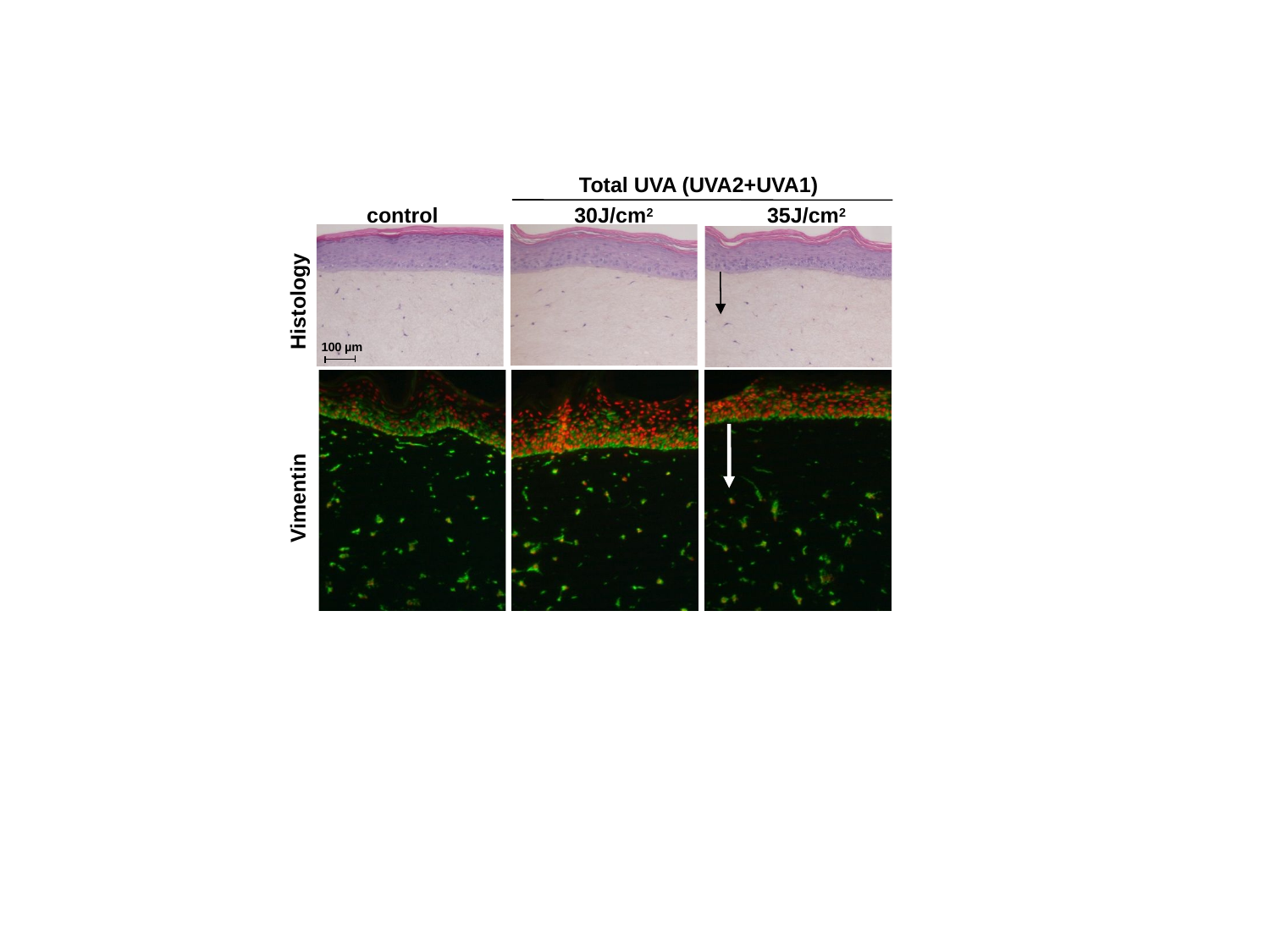

Total UVA (UVA2+UVA1)
control
30J/cm2
35J/cm2
Histology
100 µm
Vimentin

Supplement: Figure S4 — Cellular effects in human reconstructed skin exposed to total UVA (UVA2+UVA1). Sham-exposed (control) and UV-exposed samples were taken for classical histology and for vimentin staining (vimentin: green labeling, nuclei counterstaining: red labeling) at 48 h post (UVA1+UVA2) exposure (see Figure S1 for UVA1+UVA2 spectrum). Arrows indicate fibroblast disappearance in human dermal equivalent. The BED of total UVA was found to be 35–40 J/cm2 (depending on experiments). (PPTX) [file pone.0105263.s004.pptx]

## Slide 1
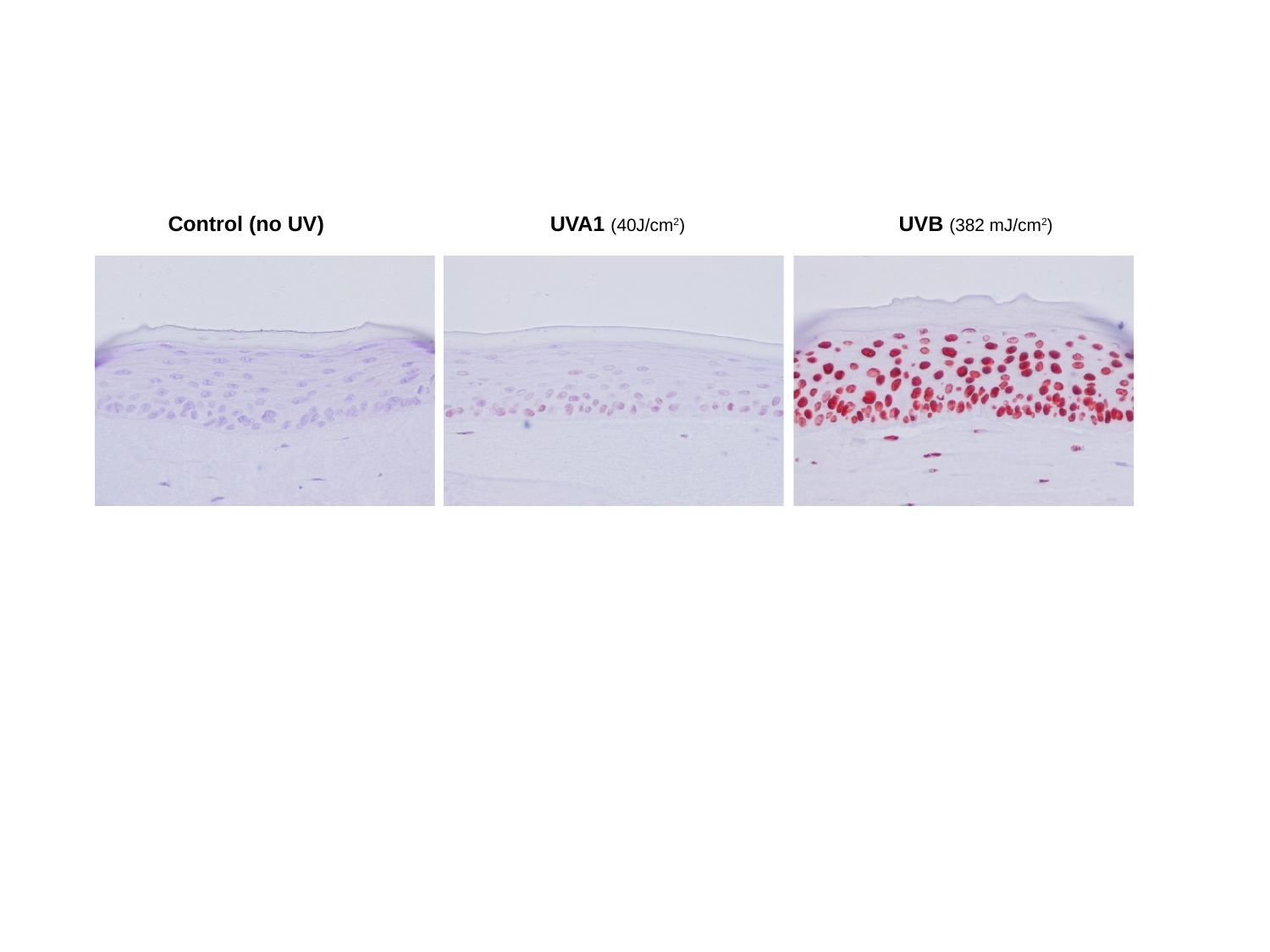

Control (no UV)
UVA1 (40J/cm2)
UVB (382 mJ/cm2)

Supplement: Figure S5 — Cyclobutane pyrimidine dimers (CPD) immunostaining in human reconstructed skin exposed to UVA1. Reconstructed skins were exposed to 40 J/cm2 UVA1 or to 382 mJ/cm2 UVB (positive control). Skin samples were harvested one hour after exposure in order to perform CPD immunostaining using a monoclonal anti-thymine dimer antibody (1∶1000, TDM2, CosmoBio, UK), a biotinylated goat anti-mouse secondary antibody (BA-9200, Vector Laboratories, UK), and Vectasein Elite ABC Kit for peroxidase detection (PK-6100, Vector Laboratories, UK). UVB-exposed reconstructed skins exhibited strong positive staining in nuclei of keratinocytes, throughout the epidermis. In UVA1 exposed reconstructed skin a lower but clear signal was detected in nuclei of basal keratinocytes compared to non exposed skin sample. (PPTX) [file pone.0105263.s005.pptx]
