# Supplementary material for: Diversity of Biological Effects Induced by Longwave UVA Rays (UVA1) in Reconstructed Skin
Source: PLoS One. 2014 Aug 20;9(8):e105263. doi: 10.1371/journal.pone.0105263 (PMC4139344; doi:10.1371/journal.pone.0105263)
Supplement: Table S1 — Primer sequences used in quantitative PCR experiments. (DOCX) [file pone.0105263.s006.docx]

**Table S1 : PCR primer sequences.**

| **Official Full Name** | [**abbrev (iHOP)**](http://www.pdg.cnb.uam.es/UniPub/iHOP/) | **Genbank** | **Size** | **Sense primer** | **Anti-sense primer** |
| --- | --- | --- | --- | --- | --- |
| Beta-2-microglobulin | B2M | NM_004048 | 228 | TTTCATCCATCCGACATTGA | CCTCCATGATGCTGCTTACA |
| Glyceraldehyde-3-phosphate dehydrogenase | GAPDH | NM_002046 | 269 | GGCTCTCCAGAACATCATCCCTGC | GGGTGTCGCTGTTGAAGTCAGAGG |
| Ribosomal protein S28 | RPS28 | NM_001031 | 204 | ccgtgtgcagcctatcaag | caagctcagcgcaacctc |
| Ribosomal protein L13a | RPL13A | NM_012423 | [483](file:///C:\Documents%20and%20Settings\fxb\Application%20Data\Microsoft\Excel\Photos%20gels\RetD149n19b.ppt) | TAAACAGGTACTGCTGGGCCGGAAGGTG | CACGTTCTTCTCGGCCTGTTTCCGTAGC |
| Ribosomal protein S9 | RPS9 | NM_001013 | 431 | GATGAGAAGGACCCACGGCGTCTGTTCG | GAGACAATCCAGCAGCCCAGGAGGGAC |
| Adenomatosis polyposis coli down-regulated 1 | APCDD1 | NM_153000 | 212 | gagaggagaacggctgtgag | gtcgtggttcttggcattct |
| Activating transcription factor 3 | ATF3 | NM_001674,NM_001030287,NM_001040619,NM_004024 | 211 | gtttgaggattttgctaacctgac | agctgcaatcttatttctttctcgt |
| Bone morphogenetic protein 2 | BMP2 | NM_001200 | 257 | CCCACTTGGAGGAGAAACAA | AGCCACAATCCAGTCATTCC |
| Chemokine (C-C motif) ligand 20 | CCL20 | NM_004591 | 199 | TGAGCTAAAAACCATGTGCTGTA | AGCATTGATGTCACAGCCTTC |
| C-type lectin domain family 2, member A | CLEC2A | NM_001130711 | 225 | GGACTGGCTTGGAGTGAGAG | TTGAATGTGGTGCCATTTGT |
| Collagen, type I, alpha 1 | COL1A1 | NM_000088 | 420 | CGATGGATTCCAGTTCGAGTA | GTTTACAGGAAGCAGACAGG |
| Colony stimulating factor 2 (granulocyte-macrophage) | CSF2 | NM_000758 | 230 | GCCAGCCACTACAAGCAGCAC | TCCCTCCAAGATGACCATCCTGAG |
| Cathepsin H | CTSH | NM_004390 | 198 | GGCAGTTGCTGGACTTTCTC | GGTGTCTTCACCCATGATCC |
| Cathepsin L1 | CTSL1 | NM_001257972, NM_001257973, NM_001912, NM_145918, NM_001257971 | 146 | TGACTTTGCATTTTCGTTTTT | TGGCTTAGAGCCCAATTATGTT |
| DNA-damage-inducible transcript 3 | DDIT3 | NM_001195053, NM_001195054, NM_001195055, NM_001195056, NM_001195057, NM_004083 | 206 | TGCCTTTCTCTTCGGACACT | TGTGACCTCTGCTGGTTCTG |
| DEAD (Asp-Glu-Ala-Asp) box polypeptide 58 | DDX58 | NM_014314 | 213 | agagcacttgtggacgcttt | tgcaatgtcaatgccttcat |
| DnaJ (Hsp40) homolog, subfamily B, member 1 | DNAJB1 | NM_006145 | 242 | CAAGAGCCCGCCCGAAAGAAG | GCTGGAATGTTGTTGGAGGTCTGG |
| FBJ murine osteosarcoma viral oncogene homolog B | FOSB | NM_001114171, NM_006732 | 179 | CCCGTTGTTAACCCTTCGTA | CTCTCCCCCATGTGTTTGTT |
| Guanylate binding protein 1, interferon-inducible | GBP1 | NM_002053 | 246 | tgcaaagatggactttaaaagatg | ccactttcctaggacttttcaca |
| Guanylate binding protein 2, interferon-inducible | GBP2 | NM_004120 | 247 | ggcaaactcctcacctggta | gacgaagcacttcctcttgg |
| Guanylate binding protein 5 | GBP5 | NM_052942, NM_001134486 | 248 | gctgactctgcgagcttctt | tcaagttgggcaagcttttt |
| Guanylate binding protein family, member 6 | GBP6 | NM_198460 | 218 | cccaactgaaggagaagctg | aaggttgtccaaggttcgtg |
| Growth differentiation factor 15 | GDF15 | NM_004864 | 207 | acaatcccatggtgctcatt | atacagctgtttgggcagga |
| GTP binding protein overexpressed in skeletal muscle | GEM | NM_005261, NM_181702 | 206 | aagctggtcctctgactcca | cactttccccatcaaccatc |
| Heme oxygenase (decycling) 1 | HMOX1 | NM_002133 | 266 | tccgatgggtccttacactc | attgcctggatgtgcttttc |
| Heat shock 70kDa protein 1A | HSPA1A | NM_005345 | 213 | cgacctgaacaagagcatca | aagatctgcgtctgcttggt |
| Heat shock 70kDa protein 6 (HSP70B') | HSPA6 | NM_002155 | 229 | CCAAGCAGACCCAGACTTTC | GCCTTACCTGTGCTCCTGTC |
| Intercellular adhesion molecule 1 | ICAM1 | NM_000201 | 213 | CACCTATGGCAACGACTCCTTCTC | GCCTCACACTTCACTGTCACCTC |
| Immediate early response 3 | IER3 | NM_003897 | 168 | tcttcaccttcgaccctctc | gcagcagaaagagaagccttt |
| Interferon-induced protein with tetratricopeptide repeats 1 | IFIT1 | NM_001548 | 252 | agtggtagaagaaacaatgcaagac | tcattcatatttccttccaatttgt |
| Interferon-induced protein with tetratricopeptide repeats 2 | IFIT2 | NM_001547 | 196 | gcgtgaagaaggtgaagagg | aatttggcaatgcaggtagg |
| Interferon-induced protein with tetratricopeptide repeats 3 | IFIT3 | NM_0010316 | 192 | gaacatgctgaccaagcaga | cagttgtgtccacccttcct |
| Insulin-like growth factor 1 (somatomedin C) | IGF1 | NM_000618 | 265 | TGGATGCTCTTCAGTTCGTG | CCTGCACTCCCTCTACTTGC |
| Interleukin 1, alpha | IL1A | NM_000575 | 283 | atcagtacctcacggctgct | aacaagtttggatgggcaac |
| Interleukin 1, beta | IL1B | NM_000576 | 291 | aaggcggccaggatataact | ctggctgatggacaggagat |
| Interleukin 6 (interferon, beta 2) | IL6 | NM_000600 | 190 | ATGTAGCCGCCCCACACAGA | CATCCATCTTTTTCAGCCAT |
| Interleukin 8 | IL8 | NM_000584 | 270 | attctctgtggtatccaagaatcag | cgtgcaatatctaggaaaatcctta |
| Jun oncogene | JUN | NM_002228 | 280 | ccacgcaagagaagaaggac | aaaagtcgcggtcactcact |
| Keratin 2 | KRT2 | NM_000423 | 341 | CCGAGGTCAAGGCCCAGTATGAGG | GTAGTCACGCAGCAGCCGCGCCAA |
| Keratin 10 | KRT10 | NM_000421 | 236 | aggccacaagtcctcctctt | tggagactttgttttccatgc |
| Leukemia inhibitory factor (cholinergic differentiation factor) | LIF | NM_002309 | 162 | CCAAGGCCCTCTGAAGTG | CATGAGGTTGTTGTGACATGG |
| Matrix metallopeptidase 1 (interstitial collagenase) | MMP1 | NM_002421 | 249 | ACTGCTGCTGCTGCTGTTCTG | TGCTTCATCACCTTCAGGGTTTCAG |
| Matrix metallopeptidase 3 (stromelysin 1, progelatinase) | MMP3 | NM_002422 | 270 | GGCACAATATGGGCACTTTAAATGAAGC | GTCTACACAGATACAGTCACTTGTCTG |
| Myxovirus (influenza virus) resistance 1, interferon-inducible protein p78 (mouse) | MX1 | NM_002462; NM_001144925; NM_001178046 | 200 | accacagaggctctcagcat | ctcagctggtcctggatctc |
| Myxovirus (influenza virus) resistance 2 (mouse) | MX2 | NM_002463 | 205 | AAGCAGTATCGAGGCAAGGA | TCGTGCTCTGAACAGTTTGG |
| NAD(P)H dehydrogenase, quinone 1 | NQO1 | NM_000903, NM_001025433, NM_001025434 | 233 | ttactatgggatggggtcca | tctcccatttttcaggcaac |
| Nuclear receptor subfamily 4, group A, member 1 | NR4A1 | NM_001202233, NM_002135, NM_173157 | 244 | GGCATGGTGAAGGAAGTTGT | CGGAGAGCAGGTCGTAGAAC |
| 2'-5'-oligoadenylate synthetase 1, 40/46kDa | OAS1 | NM_016816, NM_002534, NM_001032409 | 228 | ttgactggcggctataaacc | gagctccagggcatactgag |
| 2'-5'-oligoadenylate synthetase 2, 69/71kDa | OAS2 | NM_016817, NM_002535, NM_001032731 | 247 | tgggttggtttatccaggaa | aggatgtcacgttggcttct |
| Ornithine decarboxylase 1 | ODC1 | NM_002539 | 205 | CCCAGCGTTGGACAAATACT | TCCATAGACGCCATCATTCA |
| Odd-skipped related 2 (Drosophila) | OSR2 | NM_053001, NM_001142462 | 191 | AAGATGGGAGACCTGAGCAA | GTGTGGGTCCTCTCATGGAT |
| Prostaglandin-endoperoxide synthase 2 (prostaglandin G/H synthase and cyclooxygenase) | PTGS2 | NM_000963 | 158 | TGAGCATCTACGGTTTGCTG | TGCTTGTCTGGAACAACTGC |
| Sterile alpha motif domain containing 9 | SAMD9 | NM_017654;NM_001193307 | 200 | tgaggttttccgatttgctt | tgcacttctttgcttgttgg |
| Sterile alpha motif domain containing 9-like | SAMD9L | NM_152703 | 200 | cgagaactggtcaccacaga | ttctgggatcaggaggaatg |
| Serpin peptidase inhibitor, clade B (ovalbumin), member 2 | SERPINB2 | NM_002575 | 359 | CTTTCCGTGTAAACTCGGCTCAGCGC | GAAATTGGCCCGTCCCTTGTTGAAGG |
| Solute carrier family 7, (cationic amino acid transporter, y+ system) member 11 | SLC7A11 | NM_014331 | 228 | tttgcaccctttgacaatga | gggtccgaatagagggaaag |
| Transglutaminase 1 (K polypeptide epidermal type I, protein-glutamine-gamma-glutamyltransferase) | TGM1 | NM_000359 | 257 | GGTTACAGAGGCCCAAGATCCTCA | AAGCTCCACCTCGAGATGCCATAG |
| Toll-like receptor 3 | TLR3 | NM_003265 | 159 | CTTTCGAGAGTGCCGTCTATTTGC | CAGCATCCCAAAGGGCAAAAGG |
| Tumor necrosis factor, alpha-induced protein 3 | TNFAIP3 | NM_006290 | 256 | AAGGGTGTCTGAGCAGGAGA | AGCCAAGACGATGAAGCAGT |
| Thioredoxin interacting protein | TXNIP | NM_006472 | 180 | GCAGTGAGCATGTGGAAGAA | ATCCTTTAAGGCCCAGGAGA |
| Thioredoxin reductase 1 | TXNRD1 | NM_001093771, NM_003330, NM_182742, NM_182729, NM_182743 | 215 | aatttgcccctgtgtgctac | tcagcttgcttagaccagca |
