# Supplementary material for: Diversity of Biological Effects Induced by Longwave UVA Rays (UVA1) in Reconstructed Skin
Source: PLoS One. 2014 Aug 20;9(8):e105263. doi: 10.1371/journal.pone.0105263 (PMC4139344; doi:10.1371/journal.pone.0105263)
Supplement: Table S2 — Most significant enriched GO terms Biological Process in fibroblasts of reconstructed skin exposed to UVA1. Detailed list of the top 50 enriched GO terms related to Biological Process (BP) for the up-regulated probe sets and down-regulated probe sets in fibroblasts of reconstructed skins exposed to UVA1. GOBPID: Gene ontology identity of enriched terms. Size: total number of probes on microarray belonging to specific GO identities. Count: number of differentially expressed probe sets on microarray belonging to specific GO identities. (DOCX) [file pone.0105263.s007.docx]

**Table S2: Most significant enriched GO terms Biological Process in fibroblasts of reconstructed skin exposed to UVA1.**

**Up regulated probe sets (285)**

| **GOBPID** | **Pvalue** | **ExpCount** | **Count** | **Size** | **Term** | |  |
| --- | --- | --- | --- | --- | --- | --- | --- |
| **Response to stimulus** | |  |  |  |  | |  |
| GO:0006986 | 2.4e-12 | 1.52 | 16 | 132 | response to unfolded protein | |  |
| GO:0035966 | 5.4e-12 | 1.61 | 16 | 139 | response to topologically incorrect protein | |  |
| GO:0010033 | 5,00E-11 | 23.65 | 57 | 2048 | response to organic substance | |  |
| GO:0070887 | 2.8e-10 | 21.91 | 53 | 1897 | cellular response to chemical stimulus | |  |
| GO:0042221 | 2.8e-09 | 33.86 | 67 | 2932 | response to chemical stimulus | |  |
| GO:0030968 | 3.1e-09 | 0.97 | 11 | 84 | endoplasmic reticulum unfolded protein response | |  |
| GO:0034620 | 3.6e-09 | 0.98 | 11 | 85 | cellular response to unfolded protein | |  |
| GO:0035967 | 6.6e-09 | 1.04 | 11 | 90 | cellular response to topologically incorrect protein | |  |
| GO:0033554 | 7.4e-09 | 14.49 | 39 | 1255 | cellular response to stress | |  |
| GO:0006950 | 8,00E-09 | 35.5 | 68 | 3074 | response to stress | |  |
| GO:0071310 | 1.6e-08 | 17.51 | 43 | 1516 | cellular response to organic substance | |  |
| GO:0034976 | 7.5e-09 | 1.32 | 12 | 114 | response to endoplasmic reticulum stress | |  |
| GO:0032496 | 2,00E-08 | 2.43 | 15 | 210 | response to lipopolysaccharide | |  |
| GO:0002237 | 4.1e-08 | 2.55 | 15 | 221 | response to molecule of bacterial origin | |  |
| GO:0048583 | 8.4e-08 | 29.55 | 58 | 2559 | regulation of response to stimulus | |  |
| GO:0051716 | 9.8e-08 | 59.74 | 93 | 5173 | cellular response to stimulus | |  |
| GO:0071216 | 1.2e-07 | 1.36 | 11 | 118 | cellular response to biotic stimulus | |  |
| GO:0048584 | 2.4e-07 | 14.48 | 36 | 1254 | positive regulation of response to stimulus | |  |
| GO:0071222 | 2.7e-07 | 1.18 | 10 | 102 | cellular response to lipopolysaccharide | |  |
| **Cell death/Apoptosis** | |  |  |  |  | |  |
| GO:0008219 | 5.9e-12 | 19.78 | 53 | 1713 | cell death | |  |
| GO:0016265 | 6.3e-12 | 19.82 | 53 | 1716 | death | |  |
| GO:0010941 | 4.5e-11 | 13.3 | 41 | 1152 | regulation of cell death | |  |
| GO:0042981 | 1.8e-10 | 12.74 | 39 | 1103 | regulation of apoptotic process | |  |
| GO:0043067 | 2.5e-10 | 12.88 | 39 | 1115 | regulation of programmed cell death | |  |
| GO:0006915 | 1.7e-09 | 17.56 | 45 | 1521 | apoptotic process | |  |
| GO:0012501 | 2.3e-09 | 17.73 | 45 | 1535 | programmed cell death | |  |
| GO:0097285 | 1.3e-08 | 3.49 | 18 | 302 | cell-type specific apoptotic process | |  |
| GO:0060548 | 1.4e-07 | 7.6 | 25 | 658 | negative regulation of cell death | |  |
| GO:0043066 | 2,00E-07 | 7.19 | 24 | 623 | negative regulation of apoptotic process | |  |
| GO:0043069 | 2.5e-07 | 7.28 | 24 | 630 | negative regulation of programmed cell death | |  |
| **Signaling** | |  |  |  |  | |  |
| GO:0042325 | 3.9e-10 | 10.21 | 34 | 884 | regulation of phosphorylation | |  |
| GO:0006984 | 9,00E-10 | 1.1 | 12 | 95 | ER-nucleus signaling pathway | |  |
| GO:0001932 | 4.8e-09 | 9.54 | 31 | 826 | regulation of protein phosphorylation | |  |
| GO:0035556 | 7.3e-09 | 22.54 | 51 | 1952 | intracellular signal transduction | |  |
| GO:0009966 | 1.4e-08 | 22.24 | 50 | 1926 | regulation of signal transduction | |  |
| GO:0007243 | 2.8e-08 | 10.29 | 31 | 891 | intracellular protein kinase cascade | |  |
| GO:0007165 | 5.1e-08 | 49.02 | 82 | 4245 | signal transduction | |  |
| GO:0016310 | 1,00E-07 | 14.64 | 37 | 1268 | phosphorylation | |  |
| GO:0010627 | 1.9e-07 | 8.26 | 26 | 715 | regulation of intracellular protein kinase cascade | |  |
| GO:0023051 | 2.4e-07 | 25.06 | 51 | 2170 | regulation of signaling | |  |
| GO:0006468 | 1.6e-07 | 12.97 | 34 | 1123 | protein phosphorylation | |  |
| GO:0042327 | 2.9e-07 | 6.79 | 23 | 588 | positive regulation of phosphorylation | |  |
| **Protein modification** | |  |  |  |  | |  |
| GO:0051247 | 1.3e-08 | 10.54 | 32 | 913 | positive regulation of protein metabolic process | |  |
| GO:0031399 | 1.2e-07 | 12.19 | 33 | 1056 | regulation of protein modification process | |  |
| GO:0051246 | 1.7e-07 | 17.62 | 41 | 1526 | regulation of protein metabolic process | |  |
| **Cell communication** | |  |  |  |  | |  |
| GO:0010646 | 9.8e-08 | 25.12 | 52 | 2175 | regulation of cell communication | |  |
| GO:0007154 | 2.7e-07 | 56.24 | 88 | 4870 | cell communication | |  |
| **Regulation of metabolic process/regulation of biological process** | | | | | |  | |
| GO:0019220 | 2.6e-07 | 14.55 | 36 | 1260 | regulation of phosphate metabolic process | |  |
| GO:0051174 | 3.4e-07 | 14.7 | 36 | 1273 | regulation of phosphorus metabolic process | |  |
| GO:0032069 | 1,00E-07 | 0.81 | 9 | 70 | regulation of nuclease activity | |  |
| GO:0048519 | 1.8e-07 | 37.41 | 67 | 3240 | negative regulation of biological process | |  |

**Down-regulated probe sets (209)**

| **GOBPID** | **Pvalue** | **ExpCount** | **Count** | | **Size** | | **Term** | | |  |
| --- | --- | --- | --- | --- | --- | --- | --- | --- | --- | --- |
| **Response to virus** | |  |  | |  | |  | | |  |
| GO:0009615 | 8.1e-12 | 4.29 | 24 | | 268 | | response to virus | | |  |
| GO:0060337 | 9.5e-12 | 1.18 | 14 | | 74 | | type I interferon-mediated signaling pathway | | |  |
| GO:0071357 | 9.5e-12 | 1.18 | 14 | | 74 | | cellular response to type I interferon | | |  |
| GO:0034340 | 1.1e-11 | 1.2 | 14 | | 75 | | response to type I interferon | | |  |
| GO:0051607 | 3.2e-11 | 3.07 | 20 | | 192 | | defense response to virus | | |  |
| **Response to cytokine/Innate immunity** | | | | | |  | |  |  | |
| GO:0071345 | 9.8e-08 | 6.85 | 24 | | 428 | | cellular response to cytokine stimulus | | |  |
| GO:0034097 | 2.1e-07 | 8.22 | 26 | | 514 | | response to cytokine stimulus | | |  |
| GO:0019221 | 4.3e-07 | 5.34 | 20 | | 334 | | cytokine-mediated signaling pathway | | |  |
| GO:0045087 | 5.6e-07 | 12.22 | 32 | | 764 | | innate immune response | | |  |
| **Development/Morphogenesis** | | | |  | |  | |  |  | |
| GO:0072001 | 3.3e-09 | 3.98 | 20 | | 249 | | renal system development | | |  |
| GO:0001655 | 7.4e-09 | 4.61 | 21 | | 288 | | urogenital system development | | |  |
| GO:0061448 | 2.4e-08 | 3.22 | 17 | | 201 | | connective tissue development | | |  |
| GO:0001822 | 2.8e-08 | 3.25 | 17 | | 203 | | kidney development | | |  |
| GO:0051216 | 6.3e-08 | 2.64 | 15 | | 165 | | cartilage development | | |  |
| GO:0001501 | 1.5e-07 | 6.48 | 23 | | 405 | | skeletal system development | | |  |
| GO:0001657 | 6.2e-07 | 1.62 | 11 | | 101 | | ureteric bud development | | |  |
| GO:0048731 | 8.9e-07 | 56.11 | 89 | | 3507 | | system development | | |  |
| GO:0048729 | 1.1e-06 | 7.84 | 24 | | 490 | | tissue morphogenesis | | |  |
| GO:0002009 | 1.1e-06 | 6.21 | 21 | | 388 | | morphogenesis of an epithelium | | |  |
| GO:0048645 | 1.8e-06 | 0.85 | 8 | | 53 | | organ formation | | |  |
| GO:0051239 | 2.5e-06 | 30.99 | 57 | | 1937 | | regulation of multicellular organismal process | | |  |
| GO:0009887 | 2.6e-06 | 12.48 | 31 | | 780 | | organ morphogenesis | | |  |
| GO:0035295 | 3.1e-06 | 7.17 | 22 | | 448 | | tube development | | |  |
| GO:0048856 | 5.9e-06 | 64.78 | 96 | | 4049 | | anatomical structure development | | |  |
| GO:0022603 | 7.6e-06 | 9.97 | 26 | | 623 | | regulation of anatomical structure morphogenesis | | |  |
| GO:0032502 | 7.6e-06 | 73.34 | 105 | | 4584 | | developmental process | | |  |
| GO:0044707 | 7.3e-06 | 89.28 | 122 | | 5580 | | single-multicellular organism process | | |  |
| GO:2000026 | 7.9e-06 | 18.66 | 39 | | 1166 | | regulation of multicellular organismal development | | |  |
| GO:0032501 | 8.1e-06 | 92.35 | 125 | | 5772 | | multicellular organismal process | | |  |
| GO:0061035 | 8.9e-06 | 0.75 | 7 | | 47 | | regulation of cartilage development | | |  |
| GO:0030111 | 1.2e-05 | 3.04 | 13 | | 190 | | regulation of Wnt receptor signaling pathway | | |  |
| GO:0048706 | 1.3e-05 | 1.81 | 10 | | 113 | | embryonic skeletal system development | | |  |
| GO:0072006 | 1.3e-05 | 1.44 | 9 | | 90 | | nephron development | | |  |
| GO:0002062 | 2,00E-07 | 1.15 | 10 | | 72 | | chondrocyte differentiation | | |  |
| GO:0051674 | 2.9e-06 | 15.17 | 35 | | 948 | | localization of cell | | |  |
| GO:0003002 | 9.2e-06 | 4.91 | 17 | | 307 | | regionalization | | |  |
| GO:0007154 | 1.3e-05 | 77.92 | 109 | | 4870 | | cell communication | | |  |
| **Cell migration/Motility** | |  |  | |  | |  | | |  |
| GO:0040012 | 3.2e-08 | 7.47 | 26 | | 467 | | regulation of locomotion | | |  |
| GO:2000145 | 1.1e-07 | 6.9 | 24 | | 431 | | regulation of cell motility | | |  |
| GO:0030334 | 1.7e-07 | 6.53 | 23 | | 408 | | regulation of cell migration | | |  |
| GO:0051270 | 3.3e-07 | 7.86 | 25 | | 491 | | regulation of cellular component movement | | |  |
| GO:0016477 | 1.2e-06 | 13.94 | 34 | | 871 | | cell migration | | |  |
| GO:0048870 | 2.9e-06 | 15.17 | 35 | | 948 | | cell motility | | |  |
| **Signaling** | |  |  | |  | |  | | |  |
| GO:0007166 | 7.6e-07 | 39.25 | 69 | | 2453 | | cell surface receptor signaling pathway | | |  |
| GO:0007165 | 2.2e-06 | 67.92 | 101 | | 4245 | | signal transduction | | |  |
| **Response to stimulus** | |  |  | |  | |  | | |  |
| GO:0009607 | 7.8e-07 | 9.97 | 28 | | 623 | | response to biotic stimulus | | |  |
| GO:0071310 | 2.7e-06 | 24.26 | 48 | | 1516 | | cellular response to organic substance | | |  |
| GO:0070887 | 2.9e-06 | 30.35 | 56 | | 1897 | | cellular response to chemical stimulus | | |  |
| GO:0051707 | 3.4e-06 | 9.54 | 26 | | 596 | | response to other organism | | |  |
| GO:0051716 | 4.4e-06 | 82.77 | 116 | | 5173 | | cellular response to stimulus | | |  |

GOBPID: Gene ontology identity of enriched terms. Size: total number of probes on microarray belonging to specific GO identities**.** Count: number of differentially expressed probe sets on microarray belonging to specific GO identities
