# Supplementary material for: Diversity of Biological Effects Induced by Longwave UVA Rays (UVA1) in Reconstructed Skin
Source: PLoS One. 2014 Aug 20;9(8):e105263. doi: 10.1371/journal.pone.0105263 (PMC4139344; doi:10.1371/journal.pone.0105263)
Supplement: Table S3 — Most significant enriched GO terms Biological Process in keratinocytes of reconstructed skin exposed to UVA1. Detailed list of the top 50 enriched GO terms related to Biological Process (BP) for the up-regulated probe sets and down-regulated probe sets in keratinocytes of reconstructed skins exposed to UVA1. GOBPID: Gene ontology identity of enriched terms. Size: total number of probes on microarray belonging to specific GO identities. Count: number of differentially expressed probe sets on microarray belonging to specific GO identities. (DOCX) [file pone.0105263.s008.docx]

**Table S3: Most significant enriched GO terms Biological Process in keratinocytes of reconstructed skin exposed to UVA1.**

**Up regulated probe sets (274)**

| **GOBPID** | **Pvalue** | | | **ExpCount** | | **Count** | | | **Size** | | | | | | **Term** | | | | | | | | |  |  |
| --- | --- | --- | --- | --- | --- | --- | --- | --- | --- | --- | --- | --- | --- | --- | --- | --- | --- | --- | --- | --- | --- | --- | --- | --- | --- |
| **Response to stimulus** | | | |  | |  | | |  | | | | | |  | | | | | | | | |  |  |
| GO:0010033 | 2.3e-11 | | | 32.77 | | 72 | | | 2048 | | | | | | response to organic substance | | | | | | | | |  |  |
| GO:0070887 | 3.4e-10 | | | 30.35 | | 66 | | | 1897 | | | | | | cellular response to chemical stimulus | | | | | | | | |  |  |
| GO:0071310 | 4.7e-10 | | | 24.26 | | 57 | | | 1516 | | | | | | cellular response to organic substance | | | | | | | | |  |  |
| GO:0042221 | 6.6e-10 | | | 46.91 | | 87 | | | 2932 | | | | | | response to chemical stimulus | | | | | | | | |  |  |
| GO:0006950 | 1.4e-09 | | | 49.18 | | 89 | | | 3074 | | | | | | response to stress | | | | | | | | |  |  |
| GO:0009611 | 4.5e-08 | | | 17.76 | | 43 | | | 1110 | | | | | | response to wounding | | | | | | | | |  |  |
| GO:0048583 | 7.8e-08 | | | 40.94 | | 74 | | | 2559 | | | | | | regulation of response to stimulus | | | | | | | | |  |  |
| GO:0033993 | 2.1e-07 | | | 8.77 | | 27 | | | 548 | | | | | | response to lipid | | | | | | | | |  |  |
| GO:0032496 | 2.7e-07 | | | 3.36 | | 16 | | | 210 | | | | | | response to lipopolysaccharide | | | | | | | | |  |  |
| GO:0051716 | 3.2e-07 | | | 82.77 | | 120 | | | 5173 | | | | | | cellular response to stimulus | | | | | | | | |  |  |
| GO:0050896 | 3.9e-07 | | | 106.46 | | 144 | | | 6654 | | | | | | response to stimulus | | | | | | | | |  |  |
| GO:0048585 | 4.3e-07 | | | 13.95 | | 35 | | | 872 | | | | | | negative regulation of response to stimulus | | | | | | | | |  |  |
| GO:0009719 | 5.2e-07 | | | 18.03 | | 41 | | | 1127 | | | | | | response to endogenous stimulus | | | | | | | | |  |  |
| GO:0002237 | 5.3e-07 | | | 3.54 | | 16 | | | 221 | | | | | | response to molecule of bacterial origin | | | | | | | | |  |  |
| GO:0080134 | 6.3e-07 | | | 12.91 | | 33 | | | 807 | | | | | | regulation of response to stress | | | | | | | | |  |  |
| GO:1901700 | 1.3e-06 | | | 16.61 | | 38 | | | 1038 | | | | | | response to oxygen-containing compound | | | | | | | | |  |  |
| GO:0009617 | 3.8e-06 | | | 5.62 | | 19 | | | 351 | | | | | | response to bacterium | | | | | | | | |  |  |
| GO:0071222 | 5.3e-06 | | | 1.63 | | 10 | | | 102 | | | | | | cellular response to lipopolysaccharide | | | | | | | | |  |  |
| GO:0014070 | 6.5e-06 | | | 8.67 | | 24 | | | 542 | | | | | | response to organic cyclic compound | | | | | | | | |  |  |
| GO:0009605 | 7.5e-06 | | | 21.49 | | 43 | | | 1343 | | | | | | response to external stimulus | | | | | | | | |  |  |
| GO:1901701 | 7.8e-06 | | | 10.61 | | 27 | | | 663 | | | | | | cellular response to oxygen-containing compound | | | | | | | | |  |  |
| **Signaling** |  | | |  | |  | | |  | | | | | |  | | | | | | | | |  |  |
| GO:0009966 | 4.6e-09 | | | 30.82 | | 64 | | | 1926 | | | | | | regulation of signal transduction | | | | | | | | |  |  |
| GO:0023051 | 3.4e-08 | | | 34.72 | | 67 | | | 2170 | | | | | | regulation of signaling | | | | | | | | |  |  |
| GO:0010627 | 1.3e-07 | | | 11.44 | | 32 | | | 715 | | | | | | regulation of intracellular protein kinase cascade | | | | | | | | |  |  |
| GO:0042325 | 2,00E-07 | | | 14.14 | | 36 | | | 884 | | | | | | regulation of phosphorylation | | | | | | | | |  |  |
| GO:0007165 | 2.9e-07 | | | 67.92 | | 104 | | | 4245 | | | | | | signal transduction | | | | | | | | |  |  |
| GO:0001932 | 3.6e-07 | | | 13.22 | | 34 | | | 826 | | | | | | regulation of protein phosphorylation | | | | | | | | |  |  |
| GO:0023057 | 4.7e-07 | | | 12.13 | | 32 | | | 758 | | | | | | negative regulation of signaling | | | | | | | | |  |  |
| GO:0009968 | 5.4e-07 | | | 11.58 | | 31 | | | 724 | | | | | | negative regulation of signal transduction | | | | | | | | |  |  |
| GO:0042327 | 8.2e-07 | | | 9.41 | | 27 | | | 588 | | | | | | positive regulation of phosphorylation | | | | | | | | |  |  |
| GO:0001934 | 1.8e-06 | | | 9.2 | | 26 | | | 575 | | | | | | positive regulation of protein phosphorylation | | | | | | | | |  |  |
| GO:0007243 | 2,00E-06 | | | 14.26 | | 34 | | | 891 | | | | | | intracellular protein kinase cascade | | | | | | | | |  |  |
| GO:0023052 | 4.2e-06 | | | 76.16 | | 109 | | | 4760 | | | | | | signaling | | | | | | | | |  |  |
| GO:0044700 | 4.2e-06 | | | 76.16 | | 109 | | | 4760 | | | | | | single organism signaling | | | | | | | | |  |  |
| **Cell communication** | | | |  | |  | | |  | | | | | |  | | | | | | | | |  |  |
| GO:0010646 | 3.8e-08 | | | 34.8 | | 67 | | | 2175 | | | | | | regulation of cell communication | | | | | | | | |  |  |
| GO:0010648 | 5.2e-07 | | | 12.18 | | 32 | | | 761 | | | | | | negative regulation of cell communication | | | | | | | | |  |  |
| GO:0007154 | 1.1e-06 | | | 77.92 | | 113 | | | 4870 | | | | | | cell communication | | | | | | | | |  |  |
| **Cell death/apoptosis** |  | | |  | |  | | |  | | | | | |  | | | | | | | | |  |  |
| GO:0008219 | 6,00E-07 | | | 27.41 | | 54 | | | 1713 | | | | | | cell death | | | | | | | | |  |  |
| GO:0016265 | 6.4e-07 | | | 27.46 | | 54 | | | 1716 | | | | | | death | | | | | | | | |  |  |
| GO:0012501 | 3.8e-06 | | | 24.56 | | 48 | | | 1535 | | | | | | programmed cell death | | | | | | | | |  |  |
| GO:0006915 | 6.7e-06 | | | 24.34 | | 47 | | | 1521 | | | | | | apoptotic process | | | | | | | | |  |  |
| **Biosynthesis/metabolism (protein, glucose, phosphate)** | | | | | | | | | |  | | | | | |  | | | |  |  |  | | | |
| *Glucose metabolism* | |  | | |  | |  | | | |  | | | | | | |  | | | | |  |  |  |
| GO:0006048 | 3.2e-07 | | | 0.08 | | 4 | | | 5 | | | | | | UDP-N-acetylglucosamine biosynthetic process | | | | | | | | |  |  |
| GO:0046349 | 2.2e-06 | | | 0.11 | | 4 | | | 7 | | | | | | amino sugar biosynthetic process | | | | | | | | |  |  |
| GO:0009226 | 3.8e-06 | | | 0.26 | | 5 | | | 16 | | | | | | nucleotide-sugar biosynthetic process | | | | | | | | |  |  |
| *Phosphate metabolism* |  | | |  | |  | | |  | | | | | |  | | | | | | | | |  |  |
| GO:0051174 | 1.9e-06 | | | 20.37 | | 43 | | | 1273 | | | | | | regulation of phosphorus metabolic process | | | | | | | | |  |  |
| GO:0019220 | 3.6e-06 | | | 20.16 | | 42 | | | 1260 | | | | | | regulation of phosphate metabolic process | | | | | | | | |  |  |
| *Protein metabolism* |  | | |  | |  | | |  | | | | | |  | | | | | | | | |  |  |
| GO:0051247 | 3.4e-06 | | | 14.61 | | 34 | | | 913 | | | | | | positive regulation of protein metabolic process | | | | | | | | |  |  |
| GO:0051246 | 7.4e-06 | | | 24.42 | | 47 | | | 1526 | | | | | | regulation of protein metabolic process | | | | | | | | |  |  |
| **Development** | | | |  | |  | | |  | | | | | |  | | | | | | | | |  |  |
| GO:0044767 | 9.2e-07 | | | 57.02 | | 90 | | | 3564 | | | | | | single-organism developmental process | | | | | | | | |  |  |
| **Oxidative stress response** | | |  | | | | | | | | | |  |  | | |  | |  | | | | | |  |
| GO:0072593 | 5.2e-06 | | | | | | | 2 | 11 | | | 125 | | | reactive oxygen species metabolic process | | | | | | | | |  |  |
|  | | | | | | | | | | | | |  |  |  |  |  |  |  |  |  |  |  |  |  |

**Down- regulated probe sets (228)**

| **GOBPID** | **Pvalue** | **ExpCount** | **Count** | **Size** | **Term** |
| --- | --- | --- | --- | --- | --- |
| **Lipid metabolism** | |  |  |  |  |
| GO:0006629 | 1.9e-06 | 15.02 | 35 | 1161 | lipid metabolic process |
| GO:0044255 | 5.5e-06 | 11.1 | 28 | 858 | cellular lipid metabolic process |
| GO:0008610 | 1.2e-05 | 7.25 | 21 | 560 | lipid biosynthetic process |
| GO:0006631 | 5.7e-05 | 4.05 | 14 | 313 | fatty acid metabolic process |
| GO:0035384 | 8.1e-05 | 0.45 | 5 | 35 | thioester biosynthetic process |
| GO:0071616 | 8.1e-05 | 0.45 | 5 | 35 | acyl-CoA biosynthetic process |
| GO:0035383 | 0.00024 | 0.88 | 6 | 68 | thioester metabolic process |
| GO:0006637 | 0.00024 | 0.88 | 6 | 68 | acyl-CoA metabolic process |
| GO:0006086 | 0.00043 | 0.16 | 3 | 12 | acetyl-CoA biosynthetic process from pyruvate |
| GO:0010510 | 0.00043 | 0.16 | 3 | 12 | regulation of acetyl-CoA biosynthetic process from pyruvate |
| GO:0019216 | 0.00044 | 2.74 | 10 | 212 | regulation of lipid metabolic process |
| GO:0050812 | 0.00055 | 0.17 | 3 | 13 | regulation of acyl-CoA biosynthetic process |
| GO:0019432 | 0.00071 | 0.71 | 5 | 55 | triglyceride biosynthetic process |
| GO:0046460 | 0.00083 | 0.74 | 5 | 57 | neutral lipid biosynthetic process |
| GO:0046463 | 0.00083 | 0.74 | 5 | 57 | acylglycerol biosynthetic process |
| GO:0006085 | 0.00086 | 0.19 | 3 | 15 | acetyl-CoA biosynthetic process |
| GO:0019367 | 0.0016 | 0.06 | 2 | 5 | fatty acid elongation, saturated fatty acid |
| GO:0006633 | 0.0018 | 1.73 | 7 | 134 | fatty acid biosynthetic process |
| GO:0046486 | 0.0023 | 3.99 | 11 | 308 | glycerolipid metabolic process |
| GO:0006641 | 0.0023 | 1.35 | 6 | 104 | triglyceride metabolic process |
| GO:0035336 | 0.0027 | 0.28 | 3 | 22 | long-chain fatty-acyl-CoA metabolic process |
| GO:0006639 | 0.0029 | 1.41 | 6 | 109 | acylglycerol metabolic process |
| GO:0006638 | 0.0031 | 1.42 | 6 | 110 | neutral lipid metabolic process |
| GO:0072330 | 0.0033 | 2.46 | 8 | 190 | monocarboxylic acid biosynthetic process |
| **Response to virus** | |  |  |  |  |
| GO:0060337 | 4.9e-06 | 0.96 | 8 | 74 | type I interferon-mediated signaling pathway |
| GO:0071357 | 4.9e-06 | 0.96 | 8 | 74 | cellular response to type I interferon |
| GO:0034340 | 5.4e-06 | 0.97 | 8 | 75 | response to type I interferon |
| GO:0009615 | 0.00019 | 3.47 | 12 | 268 | response to virus |
| GO:0051607 | 2,00E-04 | 2.48 | 10 | 192 | defense response to virus |
| GO:1900245 | 5,00E-04 | 0.04 | 2 | 3 | positive regulation of MDA-5 signaling pathway |
| GO:0039533 | 0.00098 | 0.05 | 2 | 4 | regulation of MDA-5 signaling pathway |
| GO:1900246 | 0.00098 | 0.05 | 2 | 4 | positive regulation of RIG-I signaling pathway |
| GO:0006955 | 0.00027 | 16.02 | 31 | 1238 | immune response |
| GO:0045087 | 0.0021 | 9.89 | 20 | 764 | innate immune response |
| GO:0039530 | 0.0024 | 0.08 | 2 | 6 | MDA-5 signaling pathway |
| GO:0039531 | 0.0034 | 0.09 | 2 | 7 | regulation of viral-induced cytoplasmic pattern recognition receptor signaling pathway |
| GO:0039535 | 0.0034 | 0.09 | 2 | 7 | regulation of RIG-I signaling pathway |
| **Metabolic process** | |  |  |  |  |
| GO:0044710 | 0.00012 | 40.07 | 62 | 3097 | single-organism metabolic process |
| GO:0032787 | 0.00067 | 5.74 | 15 | 444 | monocarboxylic acid metabolic process |
| GO:0051193 | 7,00E-04 | 0.18 | 3 | 14 | regulation of cofactor metabolic process |
| GO:0051196 | 7,00E-04 | 0.18 | 3 | 14 | regulation of coenzyme metabolic process |
| GO:0006732 | 0.00089 | 2.48 | 9 | 192 | coenzyme metabolic process |
| GO:0044281 | 0.0014 | 33.19 | 50 | 2565 | small molecule metabolic process |
| **cell migration** | |  |  |  |  |
| GO:0010631 | 0.0028 | 1.88 | 7 | 145 | epithelial cell migration |
| GO:0090132 | 0.0028 | 1.88 | 7 | 145 | epithelium migration |
| **Response to stress** | |  |  |  |  |
| GO:0034097 | 0.0028 | 6.65 | 15 | 514 | response to cytokine stimulus |
| GO:0006952 | 0.0033 | 17.11 | 29 | 1322 | defense response |
| **Miscellanous** | |  |  |  |  |
| GO:0097411 | 0.00017 | 0.03 | 2 | 2 | hypoxia-inducible factor-1alpha signaling pathway |
| GO:0042127 | 0.0016 | 15.53 | 28 | 1200 | regulation of cell proliferation |
| GO:0000087 | 0.0024 | 0.08 | 2 | 6 | M phase of mitotic cell cycle |

GOBPID: Gene ontology identity of enriched terms. Size: total number of probes on microarray belonging to specific GO identities**.** Count: number of differentially expressed probe sets on microarray belonging to specific GO identities
