# Supplementary material for: Diversity of Biological Effects Induced by Longwave UVA Rays (UVA1) in Reconstructed Skin
Source: PLoS One. 2014 Aug 20;9(8):e105263. doi: 10.1371/journal.pone.0105263 (PMC4139344; doi:10.1371/journal.pone.0105263)
Supplement: Table S4 — Enriched KEGG pathways for the 494 probe sets found modulated in fibroblasts of reconstructed skin exposed to UVA1. KEGGID: KEGG identity of enriched terms. Size: total number of probes on microarray belonging to specific KEGGID. Count: number of differentially expressed probe sets on microarray belonging to specific KEGGID. (DOCX) [file pone.0105263.s009.docx]

**Table S4: Enriched KEGG pathways for the 494 probe sets found modulated in fibroblasts of reconstructed skin exposed to UVA1.**

| **KEGGID** | **Pvalue** | **ExpCount** | **Count** | **Size** | **Term** |
| --- | --- | --- | --- | --- | --- |
| 5200 | 4.1e-06 | 8.72 | 24 | 327 | Pathways in cancer |
| 4510 | 0.00022 | 5.28 | 15 | 198 | Focal adhesion |
| 5217 | 0.0032 | 1.47 | 6 | 55 | Basal cell carcinoma |
| 4010 | 0.0048 | 7.14 | 15 | 268 | MAPK signaling pathway |
| 5410 | 0.0059 | 2.19 | 7 | 82 | Hypertrophic cardiomyopathy (HCM) |
| 4060 | 0.0078 | 6.82 | 14 | 256 | Cytokine-cytokine receptor interaction |
| 5412 | 0.013 | 1.95 | 6 | 73 | Arrhythmogenic right ventricular cardiomyopathy (ARVC) |
| 4620 | 0.017 | 2.67 | 7 | 100 | Toll-like receptor signaling pathway |
| 4512 | 0.023 | 2.21 | 6 | 83 | ECM-receptor interaction |
| 4621 | 0.024 | 1.65 | 5 | 62 | NOD-like receptor signaling pathway |
| 5414 | 0.031 | 2.37 | 6 | 89 | Dilated cardiomyopathy |
| 5120 | 0.032 | 1.79 | 5 | 67 | Epithelial cell signaling in Helicobacter pylori infection |
| 4330 | 0.036 | 1.25 | 4 | 47 | Notch signaling pathway |
| 5211 | 0.038 | 1.87 | 5 | 70 | Renal cell carcinoma |
| 480 | 0.043 | 1.33 | 4 | 50 | Glutathione metabolism |
| 4520 | 0.049 | 2 | 5 | 75 | Adherens junction |

KEGGID: KEGG identity of enriched terms

Size: total number of probes on microarray belonging to specific KEGGID

Count: number of differentially expressed probe sets on microarray belonging to specific KEGGID
