# Supplementary material for: Diversity of Biological Effects Induced by Longwave UVA Rays (UVA1) in Reconstructed Skin
Source: PLoS One. 2014 Aug 20;9(8):e105263. doi: 10.1371/journal.pone.0105263 (PMC4139344; doi:10.1371/journal.pone.0105263)
Supplement: Table S5 — Enriched KEGG pathways for the 502 probe sets found modulated in keratinocytes of reconstructed skin exposed to UVA1. KEGGID: KEGG identity of enriched terms. Size: total number of probes on microarray belonging to specific KEGGID. Count: number of differentially expressed probe sets on microarray belonging to specific KEGGID. (DOCX) [file pone.0105263.s010.docx]

**Table S5: Enriched KEGG pathways for the 502 probe sets found modulated in keratinocytes of reconstructed skin exposed to UVA1.**

| **KEGGID** | **Pvalue** | **ExpCount** | **Count** | **Size** | **Term** |
| --- | --- | --- | --- | --- | --- |
| 4060 | 0.00088 | 8.62 | 19 | 256 | Cytokine-cytokine receptor interaction |
| 4360 | 0.0012 | 4.34 | 12 | 129 | Axon guidance |
| 4010 | 0.0015 | 9.02 | 19 | 268 | MAPK signaling pathway |
| 520 | 0.0033 | 1.48 | 6 | 44 | Amino sugar and nucleotide sugar metabolism |
| 4621 | 0.0045 | 2.09 | 7 | 62 | NOD-like receptor signaling pathway |
| 4630 | 0.0051 | 5.15 | 12 | 153 | Jak-STAT signaling pathway |
| 4144 | 0.0091 | 6.23 | 13 | 185 | Endocytosis |
| 561 | 0.017 | 1.51 | 5 | 45 | Glycerolipid metabolism |
| 5120 | 0.024 | 2.26 | 6 | 67 | Epithelial cell signaling in Helicobacter pylori infection |
| 480 | 0.025 | 1.68 | 5 | 50 | Glutathione metabolism |
| 4622 | 0.03 | 2.36 | 6 | 70 | RIG-I-like receptor signaling pathway |
| 5211 | 0.03 | 2.36 | 6 | 70 | Renal cell carcinoma |
| 4370 | 0.04 | 2.52 | 6 | 75 | VEGF signaling pathway |
| 910 | 0.04 | 0.77 | 3 | 23 | Nitrogen metabolism |
| 590 | 0.042 | 1.92 | 5 | 57 | Arachidonic acid metabolism |

KEGGID: KEGG identity of enriched terms

Size: total number of probes on microarray belonging to specific KEGGID

Count: number of differentially expressed probe sets on microarray belonging to specific KEGGID
