# Supplementary material for: Diversity of Biological Effects Induced by Longwave UVA Rays (UVA1) in Reconstructed Skin
Source: PLoS One. 2014 Aug 20;9(8):e105263. doi: 10.1371/journal.pone.0105263 (PMC4139344; doi:10.1371/journal.pone.0105263)
Supplement: Table S6 — Restricted list of the 134 genes modulated by UVA1 in fibroblasts of reconstructed skins, in Affimetrix microarrays. Selection criteria of the restricted list of modulated genes were as follows: fold change modulation threshold >2 or <0.5, and the Adjp value <0.001. Ratio values <1 were transformed as -1/ratio value, so that positive and negative values denote up-regulations (red) and down-regulations (green), respectively. Twenty-six genes were classified into two or three functional families. They were marked with an asterisk. The section “Other” includes 23 genes that could not be classified in functional families, because their functions were not enough described or determined. (DOCX) [file pone.0105263.s011.docx]

**Table S6: Restricted list of the 134 genes modulated by UVA1 in fibroblasts of reconstructed skins, in Affymetrix microarrays.**

| **Development / Cell cycle/ /apoptosis/oncogene/tumor suppressor/cancer** | | | | | | | |  | | | |  | | |  | |  | |  |
| --- | --- | --- | --- | --- | --- | --- | --- | --- | --- | --- | --- | --- | --- | --- | --- | --- | --- | --- | --- |
| **Cancer/skin cancer/oncogene/tumor suppressor** | | | | | |  |  |  | | | |  | | |  | |  | |  |
|  | NM_005252 | FOS* | 3,48 | v-fos FBJ murine osteosarcoma viral oncogene homolog | | | | | | | | | | |  | |  | |  |
|  | NM_006732 | FOSB* | 3,39 | FBJ murine osteosarcoma viral oncogene homolog B | | | | | | |  | | |  | |  |  |  |  |
|  | NM_002359 | MAFG* | 2,21 | v-maf musculoaponeurotic fibrosarcoma oncogene homolog G (avian) | | | | | | | | | |  | |  |  |  |  |
|  | NM_007314 | ABL2* | 2,33 | v-abl Abelson murine leukemia viral oncogene homolog 2 (arg, Abelson-related gene) | | | | | | | | | | | |  |  |  |  |
|  | NM_000963 | PTGS2* | 9,80 | prostaglandin-endoperoxide synthase 2 (prostaglandin G/H synthase and cyclooxygenase) | | | | |  |  | | |  | | |  | |  | |
|  | NM_182643 | DLC1* | -2,06 | deleted in liver cancer 1 | | | | |  |  | | |  | | |  | |  | |
|  | NM_175634 | RUNX1T1 | -2,04 | runt-related transcription factor 1; translocated to, 1 (cyclin D-related) | | | | | | | | | |  | |  |  |  |  |
|  | NM_030915 | LBH* | -2,94 | limb bud and heart development homolog (mouse) | | | | | | |  | | |  | |  |  |  |  |
|  | NM_002309 | LIF* | 4,00 | leukemia inhibitory factor (cholinergic differentiation factor) | | | | | | |  | | |  | |  |  |  |  |
|  | NM_001038633 | RSPO1* | -2,06 | R-spondin homolog (Xenopus laevis) |  | |  | | | |  | | |  | |  |  |  |  |
|  | NM_005985 | SNAI1* | -2,01 | snail homolog 1 (Drosophila) |  | |  | | | |  | | |  | |  |  |  |  |
| **Cell cycle/proliferation** | |  |  |  | |  |  |  | | | |  | | |  | |  | |  |
|  | NM_001111283 | IGF1 | -4,82 | insulin-like growth factor 1 (somatomedin C) | | | | | | | |  | | |  | |  | |  |
|  | NM_015714 | G0S2 | 2,33 | G0/G1switch 2 | | |  |  | | | |  | | |  | |  | |  |
|  | NM_201433 | GAS7 | -2,09 | growth arrest-specific 7 | | |  |  | | | |  | | |  | |  | |  |
| **Apoptosis** | | | | | | | | | | | | | | | | | | |  |
|  | NM_003897 | IER3 | 2,29 | immediate early response 3 | | | |  | | | |  | | |  | |  | |  |
|  | NM_014330 | PPP1R15A | 2,76 | protein phosphatase 1, regulatory (inhibitor) subunit 15A | | | | | | | | | | |  | |  | |  |
|  | NM_001165 | BIRC3 | 2,80 | baculoviral IAP repeat-containing 3 | | | |  | | | |  | | |  | |  | |  |
|  | NM_004083 | DDIT3 | 3,10 | DNA-damage-inducible transcript 3 | | | |  | | | |  | | |  | |  | |  |
|  | NM_133328 | DEDD2 | 2,03 | death effector domain containing 2 | | | |  | | | |  | | |  | |  | |  |
|  | NM_006850 | IL24 | 2,39 | interleukin 24 | | |  |  | | | |  | | |  | |  | |  |
|  | NM_002135 | NR4A1* | 3,35 | nuclear receptor subfamily 4, group A, member 1 | | | | | | | |  | | |  | |  | |  |
|  | NM_006186 | NR4A2* | 2,43 | nuclear receptor subfamily 4, group A, member 2 | | | | | | | |  | | |  | |  | |  |
|  | NM_173198 | NR4A3* | 2,11 | nuclear receptor subfamily 4, group A, member 3 | | | | | | | |  | | |  | |  | |  |
|  | NM_005168 | RND3* | 2,28 | Rho family GTPase 3 | | |  |  | | | |  | | |  | |  | |  |
| **Development** | |  |  |  | |  |  |  | | | |  | | |  | |  | |  |
|  | NM_001037954 | DIXDC1 | -2,13 | DIX domain containing 1 | | |  |  | | | |  | | |  | |  | |  |
|  | NM_005264 | GFRA1 | -2,34 | GDNF family receptor alpha 1 | | | |  | | | |  | | |  | |  | |  |
|  | NM_001038633 | RSPO1* | -2,06 | R-spondin homolog (Xenopus laevis) | | | |  | | | |  | | |  | |  | |  |
|  | NM_001083962 | TCF4* | -2,15 | transcription factor 4 | | |  |  | | | |  | | |  | |  | |  |
|  | NM_153000 | APCDD1 | -2,15 | adenomatosis polyposis coli down-regulated 1 | | | | | | | |  | | |  | |  | |  |
|  | NM_001200 | BMP2* | 2,44 | bone morphogenetic protein 2 | | | |  | | | |  | | |  | |  | |  |
|  | NM_203371 | FIBIN | -2,61 | fin bud initiation factor homolog (zebrafish) | | | | | | | |  | | |  | |  | |  |
|  | NM_017637 | BNC2 | -2,06 | basonuclin 2 | | |  |  | | | |  | | |  | |  | |  |
|  | NM_148957 | TNFRSF19 | -2,12 | tumor necrosis factor receptor superfamily, member 19 | | | | | | | | | | |  | |  | |  |
|  | NM_002397 | MEF2C* | -2,04 | myocyte enhancer factor 2C | | | |  | | | |  | | |  | |  | |  |
|  | NM_001550 | IFRD1 | 2,33 | interferon-related developmental regulator 1 | | | | | | | |  | | |  | |  | |  |
| *Wnt pathway* | | | | | | | | | | | | | | | | | | |  |
|  | NM_030915 | LBH* | -2,94 | limb bud and heart development homolog (mouse) | | | | | | | | | | |  | |  | |  |
|  | NM_153026 | PRICKLE1 | -2,32 | prickle homolog 1 (Drosophila) | | | |  | | | |  | | |  | |  | |  |
|  | NM_003882 | WISP1* | -2,12 | WNT1 inducible signaling pathway protein 1 | | | | | | | |  | | |  | |  | |  |
| *Notch pathway* | | | | | | | | | | | | | | | | | | |  |
|  | NM_018717 | MAML3 | -2,02 | mastermind-like 3 (Drosophila) | | | |  | | | |  | | |  | |  | |  |
| **Innate immunity** | | | | | | | | | | | | | | | | | | |  |
| **Inflammation** | |  |  |  | |  |  |  | | | |  | | |  | |  | |  |
|  | NM_000600 | IL6 | 5,49 | interleukin 6 (interferon, beta 2) | | | |  | | | |  | | |  | |  | |  |
|  | NM_000584 | IL8 | 2,80 | interleukin 8 | | |  |  | | | |  | | |  | |  | |  |
|  | NM_004591 | CCL20 | 6,06 | chemokine (C-C motif) ligand 20 | | | |  | | | |  | | |  | |  | |  |
|  | NM_000201 | ICAM1 | 2,30 | intercellular adhesion molecule 1 | | | |  | | | |  | | |  | |  | |  |
|  | NM_000963 | PTGS2* | 9,80 | prostaglandin-endoperoxide synthase 2 (prostaglandin G/H synthase and cyclooxygenase) | | | | | | | | | | | | | | |  |
|  | NM_002309 | LIF* | 4,00 | leukemia inhibitory factor (cholinergic differentiation factor) | | | | | | | | | | |  | |  | |  |
|  | NM_001570 | IRAK2 | 3,43 | interleukin-1 receptor-associated kinase 2 | | | | | | | |  | | |  | |  | |  |
|  | NM_002135 | NR4A1* | 3,35 | nuclear receptor subfamily 4, group A, member 1 | | | | | | | |  | | |  | |  | |  |
|  | NM_006186 | NR4A2* | 2,43 | nuclear receptor subfamily 4, group A, member 2 | | | | | | | |  | | |  | |  | |  |
|  | NM_173198 | NR4A3* | 2,11 | nuclear receptor subfamily 4, group A, member 3 | | | | | | | |  | | |  | |  | |  |
|  | NM_172220 | CSF3 | 2,35 | colony stimulating factor 3 (granulocyte) | | | | | | | |  | | |  | |  | |  |
|  | NM_004864 | GDF15* | 5,20 | growth differentiation factor 15 | |  |  |  | | | |  | | |  | |  | |  |
| *TNF pathway* | | | | | | | | | | | | | | | | | | |  |
|  | NM_006290 | TNFAIP3 | 2,29 | tumor necrosis factor, alpha-induced protein 3 | | | | | | | |  | | |  | |  | |  |
|  | NM_007115 | TNFAIP6 | 2,07 | tumor necrosis factor, alpha-induced protein 6 | | | | | | | |  | | |  | |  | |  |
| **Antiviral/Bacterial Recognition/Defense** | | | | | | | | | | | | | | | | | | |  |
| *Interferon inducible genes* | | |  |  | |  |  |  | | | |  | | |  | |  | |  |
|  | NM_017654 | SAMD9 | -2,17 | sterile alpha motif domain containing 9 | | | | | | | |  | | |  | |  | |  |
|  | NM_152703 | SAMD9L | -2,95 | sterile alpha motif domain containing 9-like | | | | | | | |  | | |  | |  | |  |
|  | NM_001548 | IFIT1 | -3,78 | interferon-induced protein with tetratricopeptide repeats 1 | | | | | | | | | | |  | |  | |  |
|  | NM_001547 | IFIT2 | -2,82 | interferon-induced protein with tetratricopeptide repeats 2 | | | | | | | | | | |  | |  | |  |
|  | NM_001031683 | IFIT3 | -2,52 | interferon-induced protein with tetratricopeptide repeats 3 | | | | | | | | | | |  | |  | |  |
|  | NM_002462 | MX1 | -2,51 | myxovirus (influenza virus) resistance 1, interferon-inducible protein p78 (mouse) | | | | | | | | | | | | | | |  |
|  | NM_002463 | MX2 | -3,45 | myxovirus (influenza virus) resistance 2 (mouse) | | | | | | | |  | | |  | |  | |  |
|  | NM_016816 | OAS1 | -2,24 | 2',5'-oligoadenylate synthetase 1, 40/46kDa | | | | | | | |  | | |  | |  | |  |
|  | NM_002535 | OAS2 | -2,14 | 2'-5'-oligoadenylate synthetase 2, 69/71kDa | | | | | | | |  | | |  | |  | |  |
|  | NM_002053 | GBP1 | -2,23 | guanylate binding protein 1, interferon-inducible, 67kDa | | | | | | | | | | |  | |  | |  |
|  | NM_004120 | GBP2 | -2,01 | guanylate binding protein 2, interferon-inducible | | | | | | | |  | | |  | |  | |  |
|  | NM_052942 | GBP5 | -2,10 | guanylate binding protein 5 | | | |  | | | |  | | |  | |  | |  |
|  | NM_001002264 | EPSTI1* | -2,14 | epithelial stromal interaction 1 (breast) | | | | | | | |  | | |  | |  | |  |
|  | NM_015474 | SAMHD1 | -2,03 | SAM domain and HD domain 1 | | | |  | | | |  | | |  | |  | |  |
| *ds RNA receptors* | | | | | | |  |  | | | |  | | |  | |  | |  |
|  | NM_014314 | DDX58 | -2,67 | DEAD (Asp-Glu-Ala-Asp) box polypeptide 58 | | | | | | | |  | | |  | |  | |  |
|  | NM_003265 | TLR3 | -2,24 | toll-like receptor 3 | | |  |  | | | |  | | |  | |  | |  |
| **Immune response** | |  |  |  | |  |  |  | | | |  | | |  | |  | |  |
|  | NM_006895 | HNMT | -2,08 | histamine N-methyltransferase | | | |  | | | |  | | |  | |  | |  |
|  | NM_004072 | CMKLR1 | -3,82 | chemokine-like receptor 1 | | | |  | | | |  | | |  | |  | |  |
|  | NM_031458 | PARP9 | -2,28 | poly (ADP-ribose) polymerase family, member 9 | | | | | | | |  | | |  | |  | |  |
|  | NM_001776 | ENTPD1 | -2,48 | ectonucleoside triphosphate diphosphohydrolase 1 | | | | | | | | | | |  | |  | |  |
| **Extra cellular Matrix/Wound repair/ Epithelial-mesenchymal transition/Dermis epidermis cross talk / Epidermal Differentiation** | | | | | | | | | | | | | | | | |  | |  |
| **Extra cellular Matrix** | | | |  | | | | | | | |  | | |  | |  | |  |
|  | NM_003882 | WISP1* | -2,12 | WNT1 inducible signaling pathway protein 1 | | | | | | | |  | | |  | |  | |  |
| *TGF pathway* | | | | | | | | | | | | | | | | | | |  |
|  | NM_001200 | BMP2* | 2,44 | bone morphogenetic protein 2 | | | |  | | | |  | | |  | |  | |  |
|  | *NM_004864* | GDF15* | 5,20 | growth differentiation factor 15 | | | |  | | | |  | | |  | |  | |  |
| *Growth factors* | |  |  |  | |  |  |  | | | |  | | |  | |  | |  |
|  | NM_000800 | FGF1 | -2,28 | fibroblast growth factor 1 (acidic) | | | |  | | | |  | | |  | |  | |  |
|  | NM_004469 | FIGF | -3,11 | c-fos induced growth factor (vascular endothelial growth factor D) | | | | | | | | | | | | |  | |  |
|  | NM_000601 | HGF* | -2,40 | hepatocyte growth factor (hepapoietin A; scatter factor) | | | | | | | | | | |  | |  | |  |
| **Wound repair** | | | | | | | | | | | | | | | | | | |  |
|  | NM_001083962 | TCF4* | -2,15 | transcription factor 4 | | |  |  | | | |  | | |  | |  | |  |
|  | NM_000129 | F13A1 | -3,16 | coagulation factor XIII, A1 polypeptide | | | | | | | |  | | |  | |  | |  |
|  | NM_002309 | LIF* | 4,00 | leukemia inhibitory factor (cholinergic differentiation factor) | | | | | | | | | | |  | |  | |  |
| **Epithelial-mesenchymal transition- dermis epidermis cross talk** | | | | | |  |  |  | | | |  | | |  | |  | |  |
|  | NM_005985 | SNAI1* | -2,01 | snail homolog 1 (Drosophila) | | | |  | | | |  | | |  | |  | |  |
|  | NM_005252 | FOS* | 3,48 | v-fos FBJ murine osteosarcoma viral oncogene homolog | | | | | | | | | | |  | |  | |  |
|  | NM_006732 | FOSB* | 3,39 | FBJ murine osteosarcoma viral oncogene homolog B | | | | | | | | | | |  | |  | |  |
|  | NM_001002264 | EPSTI1* | -2,14 | epithelial stromal interaction 1 (breast) | | | | | | | |  | | |  | |  | |  |
| **Epidermal Differentiation** | | | | | | | | | | | | | | | | | | |  |
|  | NM_002359 | MAFG* | 2,21 | v-maf musculoaponeurotic fibrosarcoma oncogene homolog G (avian) | | | | | | | | | | | | |  | |  |
|  | NM_002758 | MAP2K6* | -2,55 | mitogen-activated protein kinase kinase 6 | | | | | | | |  | | |  | |  | |  |
| **Intracellular signalling** | | | | | | | | | | | | | | | | | | |  |
|  | NM_005261 | GEM | 2,64 | GTP binding protein overexpressed in skeletal muscle | | | | | | | | | | |  | |  | |  |
|  | NM_053064 | GNG2 | -2,54 | guanine nucleotide binding protein (G protein), gamma 2 | | | | | | | | | | |  | |  | |  |
|  | NM_000856 | GUCY1A3 | -2,32 | guanylate cyclase 1, soluble, alpha 3 | | | |  | | | |  | | |  | |  | |  |
|  | NM_018945 | PDE7B | -2,43 | phosphodiesterase 7B | | |  |  | | | |  | | |  | |  | |  |
|  | NM_002758 | MAP2K6* | -2,55 | mitogen-activated protein kinase kinase 6 | | | | | | | |  | | |  | |  | |  |
|  | NM_001098512 | PRKG1 | -2,11 | protein kinase, cGMP-dependent, type I | | | | | | | |  | | |  | |  | |  |
|  | NM_023940 | RASL11B | -3,03 | RAS-like, family 11, member B | | | |  | | | |  | | |  | |  | |  |
|  | NM_018440 | PAG1 | 2,10 | phosphoprotein associated with glycosphingolipid microdomains 1 | | | | | | | | | | | | |  | |  |
|  | NM_002923 | RGS2* | 2,00 | regulator of G-protein signaling 2, 24kDa | | | | | | | |  | | |  | |  | |  |
|  | NM_005168 | RND3* | 2,28 | Rho family GTPase 3 | | |  |  | | | |  | | |  | |  | |  |
| **Oxidative stress response** | | | | | | | | | | | | | | | | | | |  |
|  | NM_002061 | GCLM | 2,38 | glutamate-cysteine ligase, modifier subunit | | | | | | | |  | | |  | |  | |  |
|  | NM_002133 | HMOX1* | 6,55 | heme oxygenase (decycling) 1 | | | |  | | | |  | | |  | |  | |  |
|  | NM_003330 | TXNRD1 | 3,39 | thioredoxin reductase 1 | | |  |  | | | |  | | |  | |  | |  |
|  | NM_003900 | SQSTM1* | 2,17 | sequestosome 1 | | |  |  | | | |  | | |  | |  | |  |
|  | NM_007314 | ABL2* | 2,33 | v-abl Abelson murine leukemia viral oncogene homolog 2 (arg, Abelson-related gene) | | | | | | | | | | | | | | |  |
|  | NM_002359 | MAFG* | 2,21 | v-maf musculoaponeurotic fibrosarcoma oncogene homolog G (avian) | | | | | | | | | | | | |  | |  |
|  | NM_014331 | SLC7A11* | 7,63 | solute carrier family 7, (cationic amino acid transporter, y+ system) member 11 | | | | | | | | | | | | | | |  |
|  | NM_016931 | NOX4 | -2,70 | NADPH oxidase 4 | | |  |  | | | |  | | |  | |  | |  |
| **Transport/ Calcium/ Ion /amino acids** | | | | | | | | | | | | | | | | | | |  |
|  | NM_014331 | SLC7A11* | 7,63 | solute carrier family 7, (cationic amino acid transporter, y+ system) member 11 | | | | | | | | | | | | | | |  |
|  | NM_006996 | SLC19A2 | 2,33 | solute carrier family 19 (thiamine transporter), member 2 | | | | | | | | | | |  | |  | |  |
|  | NM_001128431 | SLC39A14 | 2,13 | solute carrier family 39 (zinc transporter), member 14 | | | | | | | | | | |  | |  | |  |
|  | NM_001012661 | SLC3A2 | 2,11 | solute carrier family 3 (activators of dibasic and neutral amino acid transport), member 2 | | | | | | | | | | | | | | |  |
|  | NM_181785 | SLC46A3 | -2,04 | solute carrier family 46, member 3 | | | |  | | | |  | | |  | |  | |  |
|  | NM_001040624 | NCALD | -2,02 | neurocalcin delta | | |  |  | | | |  | | |  | |  | |  |
|  | NM_001001323 | ATP2B1 | 2,14 | ATPase, Ca++ transporting, plasma membrane 1 | | | | | | | |  | | |  | |  | |  |
| **Stress** | | | | | | | | | | | | | | | | | | |  |
|  | NM_006145 | DNAJB1 | 2,54 | DnaJ (Hsp40) homolog, subfamily B, member 1 | | | | | | | |  | | |  | |  | |  |
|  | NM_012328 | DNAJB9 | 4,25 | DnaJ (Hsp40) homolog, subfamily B, member 9 | | | | | | | |  | | |  | |  | |  |
|  | NM_005345 | HSPA1A | 3,57 | heat shock 70kDa protein 1A | | | |  | | | |  | | |  | |  | |  |
|  | NM_005346 | HSPA1B | 4,40 | heat shock 70kDa protein 1B | | | |  | | | |  | | |  | |  | |  |
|  | NM_002155 | HSPA6 | 13,16 | heat shock 70kDa protein 6 (HSP70B') | | | |  | | | |  | | |  | |  | |  |
|  | NM_014365 | HSPB8 | 2,19 | heat shock 22kDa protein 8 | | | |  | | | |  | | |  | |  | |  |
|  | NM_002133 | HMOX1* | 6,55 | heme oxygenase (decycling) 1 | | | |  | | | |  | | |  | |  | |  |
| **Lipid metabolism** | | | | | | | | | | | | | | | | | | |  |
|  | NM_152310 | ELOVL3 | -2,36 | elongation of very long chain fatty acids (FEN1/Elo2, SUR4/Elo3, yeast)-like 3 | | | | | | | | | | | | |  | |  |
|  | NM_001277 | CHKA | 2,33 | choline kinase alpha | | |  |  | | | |  | | |  | |  | |  |
|  | NM_001006630 | CHRM2 | -2,24 | cholinergic receptor, muscarinic 2 | | | |  | | | |  | | |  | |  | |  |
|  | NM_002612 | PDK4 | -2,14 | pyruvate dehydrogenase kinase, isozyme 4 | | | | | | | |  | | |  | |  | |  |
|  | NM_013261 | PPARGC1A | -2,00 | peroxisome proliferator-activated receptor gamma, coactivator 1 alpha | | | | | | | | | | | | |  | |  |
|  | NM_024560 | ACSS3 | -2,15 | acyl-CoA synthetase short-chain family member 3 | | | | | | | |  | | |  | |  | |  |
| **Smooth muscle biology** | | | | | | | | | | | | | | | | | | |  |
|  | NM_012134 | LMOD1* | -2,51 | leiomodin 1 (smooth muscle) | | | |  | | | |  | | |  | |  | |  |
|  | NM_002397 | MEF2C* | -2,04 | myocyte enhancer factor 2C | | | |  | | | |  | | |  | |  | |  |
|  | NM_053025 | MYLK* | -2,06 | myosin light chain kinase | | |  |  | | | |  | | |  | |  | |  |
|  |  |  |  |  | |  |  |  | | | |  | | |  | |  | |  |
| **Regulation of gene/protein expression** | | | | | | | | | | | | | | | | | | |  |
|  | NM_004836 | EIF2AK3 | 2,26 | eukaryotic translation initiation factor 2-alpha kinase 3 | | | | | | | | | | |  | |  | |  |
|  | NM_017631 | DDX60 | -2,23 | DEAD (Asp-Glu-Ala-Asp) box polypeptide 60 | | | | | | | |  | | |  | |  | |  |
|  | NM_001012967 | DDX60L | -2,07 | DEAD (Asp-Glu-Ala-Asp) box polypeptide 60-like | | | | | | | |  | | |  | |  | |  |
| **Cytoskeleton** | | | | | | | | | | | | | | | | | | |  |
|  | NM_012134 | LMOD1* | -2,51 | leiomodin 1 (smooth muscle) | | | |  | | | |  | | |  | |  | |  |
|  | NM_053025 | MYLK* | -2,06 | myosin light chain kinase | | |  |  | | | |  | | |  | |  | |  |
|  | NM_182643 | DLC1* | -2,06 | deleted in liver cancer 1 | | |  |  | | | |  | | |  | |  | |  |
| **Pigmentation** | | | | | | | | | | | | | | | | | | |  |
|  | NM_000601 | HGF* | -2,40 | hepatocyte growth factor (hepapoietin A; scatter factor) | | | | | | | | | | |  | |  | |  |
|  | NM_014331 | SLC7A11* | 7,63 | solute carrier family 7, (cationic amino acid transporter, y+ system) member 11 | | | | | | | | | | | | | | |  |
| **Proteasome** | | | | | | | | | | | | | | | | | | |  |
|  | AK303463 | USP41 | -2,26 | ubiquitin specific peptidase 41 | | | |  | | | |  | | |  | |  | |  |
|  | NM_003900 | SQSTM1* | 2,17 | sequestosome 1 | | |  |  | | | |  | | |  | |  | |  |
| **Hair growth** | | | | | | | | | | | | | | | | | | |  |
|  | NM_000949 | PRLR | -2,29 | prolactin receptor | | |  |  | | | |  | | |  | |  | |  |
| **Vasoconstriction** | | | | | | | | | | | | | | | | | | |  |
|  | NM_002923 | RGS2* | 2,00 | regulator of G-protein signaling 2, 24kDa | | | | | | | |  | | |  | |  | |  |
| **Angiogenesis** | | | | | | | | | | | | | | | | | | |  |
|  | NM_000601 | HGF* | -2,40 | hepatocyte growth factor (hepapoietin A; scatter factor) | | | | | | | | | | |  | |  | |  |
| **Other** | | | | | | | | | | | | | | | | | | |  |
|  | NM_015886 | PI15 | -2,31 | peptidase inhibitor 15 | | |  |  | | | |  | | |  | |  | |  |
|  | NM_016619 | PLAC8 | -2,27 | placenta-specific 8 | | |  |  | | | |  | | |  | |  | |  |
|  | NM_152495 | CNIH3 | 2,01 | cornichon homolog 3 (Drosophila) | | | |  | | | |  | | |  | |  | |  |
|  | NM_020354 | ENTPD7 | 2,09 | ectonucleoside triphosphate diphosphohydrolase 7 | | | | | | | | | | |  | |  | |  |
|  | NM_033397 | ITPRIP | 2,04 | inositol 1,4,5-triphosphate receptor interacting protein | | | | | | | | | | |  | |  | |  |
|  | NM_014851 | KLHL21 | 2,12 | kelch-like 21 (Drosophila) | | | |  | | | |  | | |  | |  | |  |
|  | AK093957 | LOC728264 | -2,49 | hypothetical protein LOC728264 | | | |  | | | |  | | |  | |  | |  |
|  | AF176921 | MST131 | 2,11 | MSTP131 | |  |  |  | | | |  | | |  | |  | |  |
|  | NM_198474 | OLFML1 | -3,26 | olfactomedin-like 1 | | |  |  | | | |  | | |  | |  | |  |
|  | NM_005068 | SIM1 | -3,29 | single-minded homolog 1 (Drosophila) | | | | | | | |  | | |  | |  | |  |
|  | NM_198152 | UTS2D | -2,35 | urotensin 2 domain containing | | | |  | | | |  | | |  | |  | |  |
|  | NM_053276 | VIT | -2,03 | vitrin | |  |  |  | | | |  | | |  | |  | |  |
|  | NM_182491 | ZFAND2A | 2,70 | zinc finger, AN1-type domain 2A | | | |  | | | |  | | |  | |  | |  |
|  | NM_020747 | ZNF608 | -2,12 | zinc finger protein 608 | | |  |  | | | |  | | |  | |  | |  |
|  | NM_017565 | FAM20A | -2,33 | family with sequence similarity 20, member A | | | | | | | |  | | |  | |  | |  |
|  | NM_153711 | FAM26E | -2,15 | family with sequence similarity 26, member E | | | | | | | |  | | |  | |  | |  |
|  | NM_153690 | FAM43A | -2,03 | family with sequence similarity 43, member A | | | | | | | |  | | |  | |  | |  |
|  | NM_016323 | HERC5 | -2,36 | hect domain and RLD 5 | | |  |  | | | |  | | |  | |  | |  |
|  | NM_017912 | HERC6 | -2,14 | hect domain and RLD 6 | | |  |  | | | |  | | |  | |  | |  |
|  | NM_001003793 | RBMS3 | -2,29 | RNA binding motif, single stranded interacting protein | | | | | | | | | | |  | |  | |  |
|  | NM_024007 | EBF1 | -2,27 | early B-cell factor 1 | | |  |  | | | |  | | |  | |  | |  |
|  | NM_175839 | SMOX | 2,01 | spermine oxidase | | |  |  | | | |  | | |  | |  | |  |
|  | NM_018266 | TMEM39A | 2,07 | transmembrane protein 39A | | | |  | | | |  | | |  | |  | |  |
|  | NM_015310 | PSD3 | -2,01 | pleckstrin and Sec7 domain containing 3 | | |  |  | | | |  | | |  | |  | |  |
