# Supplementary material for: Diversity of Biological Effects Induced by Longwave UVA Rays (UVA1) in Reconstructed Skin
Source: PLoS One. 2014 Aug 20;9(8):e105263. doi: 10.1371/journal.pone.0105263 (PMC4139344; doi:10.1371/journal.pone.0105263)
Supplement: Table S7 — Restricted list of the 141 genes modulated by UVA1 in keratinocytes of reconstructed skins, in Affimetrix microarrays. Selection criteria of the restricted list of modulated genes were as follows: fold change modulation threshold >2 or <0.5, and the Adjp value <0.001. Ratio values <1 were transformed as -1/ratio value, so that positive and negative values denote up-regulations (red) and down-regulations (green), respectively. Eleven genes were classified into two or three functional families. They were marked with an asterisk. The section “Other” includes 30 genes that could not be classified in functional families, because their functions were not enough described or determined. (DOCX) [file pone.0105263.s012.docx]

**Table S7: Restricted list of the 141 genes modulated by UVA1 in keratinocytes of reconstructed skins, in Affymetrix microarrays.**

| **Innate Immunity** | | | | |
| --- | --- | --- | --- | --- |
| **Inflammation** | | | | |
|  | NM_000584 | IL8 | 2,43 | interleukin 8 |
|  | NM_018724 | IL20 | 2,92 | interleukin 20 |
|  | NM_004591 | CCL20 | 7,69 | chemokine (C-C motif) ligand 20 |
|  | NM_000963 | PTGS2* | 4,34 | prostaglandin-endoperoxide synthase 2 (prostaglandin G/H synthase and cyclooxygenase) |
|  | NM_003764 | STX11* | 3,00 | syntaxin 11 |
|  | NM_000640 | IL13RA2 | 4,93 | interleukin 13 receptor, alpha 2 |
|  | NM_002350 | LYN* | 2,10 | v-yes-1 Yamaguchi sarcoma viral related oncogene homolog |
|  | NM_004864 | GDF15* | 2,99 | growth differentiation factor 15 |
|  | NM_001712 | CEACAM1 | 3,82 | carcinoembryonic antigen-related cell adhesion molecule 1 (biliary glycoprotein) |
|  | NM_014439 | IL1F7 | -3,27 | interleukin 1 family, member 7 (zeta) |
| *TNF pathway* | | | | |
|  | NM_025218 | ULBP1 | 3,08 | UL16 binding protein 1 |
|  | NM_006290 | TNFAIP3 | 3,27 | tumor necrosis factor, alpha-induced protein 3 |
|  | NM_000594 | TNF | 2,34 | tumor necrosis factor (TNF superfamily, member 2) |
| *Associated with inflammatory process/strongly induced in psoriasis* | | | | |
|  | NM_003937 | KYNU | 2,02 | kynureninase (L-kynurenine hydrolase) |
|  | NM_001085 | SERPINA3 | -2,45 | serpin peptidase inhibitor, clade A (alpha-1 antiproteinase, antitrypsin), member 3 |
|  | NM_004529 | MLLT3 | -2,12 | myeloid/lymphoid or mixed-lineage leukemia (trithorax homolog, Drosophila); translocated to, 3 |
| **Antiviral/Bacterial Recognition/Defense** | | | | |
| *Interferon inducible genes* | | | | |
|  | NM_006417 | IFI44 | -2,18 | interferon-induced protein 44 |
|  | NM_006820 | IFI44L | -2,37 | interferon-induced protein 44-like |
|  | NM_001548 | IFIT1 | -4,09 | interferon-induced protein with tetratricopeptide repeats 1 |
|  | NM_001031683 | IFIT3 | -2,31 | interferon-induced protein with tetratricopeptide repeats 3 |
|  | NM_002462 | MX1 | -2,08 | myxovirus (influenza virus) resistance 1, interferon-inducible protein p78 (mouse) |
|  | NM_002463 | MX2 | -2,53 | myxovirus (influenza virus) resistance 2 (mouse) |
|  | NM_152703 | SAMD9L | -2,26 | sterile alpha motif domain containing 9-like |
|  | NM_004120 | GBP2 | -2,12 | guanylate binding protein 2, interferon-inducible |
|  | NM_198460 | GBP6 | -2,10 | guanylate binding protein family, member 6 |
|  | NM_001565 | CXCL10 | -2,88 | chemokine (C-X-C motif) ligand 10 |
| *dsRNA receptor* | | | | |
|  | NM_003265 | TLR3 | -2,38 | toll-like receptor 3 |
| *C-type lectin-like receptors (CTLR)* | | | | |
|  | NM_001130711 | CLEC2A | -4,18 | C-type lectin domain family 2, member A |
|  | NM_005127 | CLEC2B | -4,53 | C-type lectin domain family 2, member B |
| **Immune response** | | | | |
|  | NM_006577 | B3GNT2 | 2,70 | UDP-GlcNAc:betaGal beta-1,3-N-acetylglucosaminyltransferase 2 |
|  | NM_014069 | PSORS1C2 | -2,05 | psoriasis susceptibility 1 candidate 2 |
| **Development/Cell cycle/Apoptosis/Oncogene/Tumor suppressor/Cancer** | | | | |
| **Cancer/skin cancer/oncogene/tumor suppressor** | | | | |
|  | NM_012449 | STEAP1 | 2,02 | six transmembrane epithelial antigen of the prostate 1 |
|  | NM_182606 | TMPRSS11A | -2,02 | transmembrane protease, serine 11A |
|  | NM_002539 | ODC1 | 2,70 | ornithine decarboxylase 1 |
|  | NM_153487 | MDGA1 | 2,13 | MAM domain containing glycosylphosphatidylinositol anchor 1 |
|  | NM_004390 | CTSH | -2,53 | cathepsin H |
|  | NM_001912 | CTSL1 | 3,12 | cathepsin L1 |
|  | NM_005238 | ETS1 | 2,12 | v-ets erythroblastosis virus E26 oncogene homolog 1 (avian) |
|  | NM_001127500 | MET | 2,21 | met proto-oncogene (hepatocyte growth factor receptor) |
|  | NM_007314 | ABL2* | 2,07 | v-abl Abelson murine leukemia viral oncogene homolog 2 (arg, Abelson-related gene) |
|  | NM_000963 | PTGS2* | 4,34 | prostaglandin-endoperoxide synthase 2 (prostaglandin G/H synthase and cyclooxygenase) |
|  | NM_014375 | FETUB | -2,14 | fetuin B |
|  | NM_206963 | RARRES1 | -2,52 | retinoic acid receptor responder (tazarotene induced) 1 |
| **Development/Morphogenesis** | | | | |
|  | NM_005098 | MSC | 2,54 | musculin (activated B-cell factor-1) |
|  | NM_001200 | BMP2* | 2,62 | bone morphogenetic protein 2 |
|  | NM_053001 | OSR2 | -2,15 | odd-skipped related 2 (Drosophila) |
|  | NM_002585 | PBX1 | -2,10 | pre-B-cell leukemia homeobox 1 |
|  | NM_013259 | TAGLN3 | 2,35 | transgelin 3 |
|  | NM_017893 | SEMA4G | -2,13 | sema domain, immunoglobulin domain (Ig), transmembrane domain (TM) and short cytoplasmic domain, (semaphorin) 4G |
|  | NM_175078 | KRT77 | -2,37 | keratin 77 |
| **Apoptosis** | | | | |
|  | NM_001165 | BIRC3 | 3,32 | baculoviral IAP repeat-containing 3 |
|  | NM_004083 | DDIT3 | 2,31 | DNA-damage-inducible transcript 3 |
|  | NM_014330 | PPP1R15A | 2,03 | protein phosphatase 1, regulatory (inhibitor) subunit 15A |
|  | NM_006850 | IL24 | 3,01 | interleukin 24 |
|  | NM_002350 | LYN* | 2,10 | v-yes-1 Yamaguchi sarcoma viral related oncogene homolog |
|  | NM_022073 | EGLN3* | -2,03 | egl nine homolog 3 (C, elegans) |
| **Cell cycle/Proliferation/Differentiation** | | | | |
|  | NM_005192 | CDKN3 | -2,04 | cyclin-dependent kinase inhibitor 3 |
|  | NM_025195 | TRIB1 | 2,07 | tribbles homolog 1 (Drosophila) |
|  | NM_001099772 | CYP4B1 | -2,05 | cytochrome P450, family 4, subfamily B, polypeptide 1 |
|  | NM_002275 | KRT15 | -2,55 | keratin 15 |
|  | NM_000423 | KRT2 | -2,13 | keratin 2 |
|  | NM_002192 | INHBA | 2,63 | inhibin, beta A |
| **Oxidative stress response** | | | | |
|  | NM_001354 | AKR1C2 | 2,36 | aldo-keto reductase family 1, member C2 (dihydrodiol dehydrogenase 2; bile acid binding protein; 3-alpha hydroxysteroid dehydrogenase, type III) |
|  | NM_003739 | AKR1C3 | 2,99 | aldo-keto reductase family 1, member C3 (3-alpha hydroxysteroid dehydrogenase, type II) |
|  | NM_000146 | FTL | 2,40 | ferritin, light polypeptide |
|  | NM_002061 | GCLM | 2,62 | glutamate-cysteine ligase, modifier subunit |
|  | NM_000903 | NQO1 | 3,23 | NAD(P)H dehydrogenase, quinone 1 |
|  | NM_000433 | NCF2 | 2,69 | neutrophil cytosolic factor 2 |
|  | NM_007314 | ABL2* | 2,07 | v-abl Abelson murine leukemia viral oncogene homolog 2 (arg, Abelson-related gene) |
|  | NM_003900 | SQSTM1* | 2,22 | sequestosome 1 |
|  | NM_014331 | SLC7A11* | 2,49 | solute carrier family 7, (cationic amino acid transporter, y+ system) member 11 |
|  | NM_007207 | DUSP10* | 2,23 | dual specificity phosphatase 10 |
|  | NM_003046 | SLC7A2* | 2,92 | solute carrier family 7 (cationic amino acid transporter, y+ system), member 2 |
|  | NM_001172 | ARG2 | 2,00 | arginase, type II |
|  | NM_006472 | TXNIP | -5,66 | thioredoxin interacting protein |
|  | NM_022073 | EGLN3* | -2,03 | egl nine homolog 3 (C, elegans) |
| **Oxidoreduction/Detoxification** | | | | |
|  | NM_014324 | AMACR | -2,17 | alpha-methylacyl-CoA racemase |
|  | NM_001460 | FMO2 | -2,64 | flavin containing monooxygenase 2 (non-functional) |
|  | NM_001461 | FMO5 | -2,06 | flavin containing monooxygenase 5 |
|  | NM_152908 | SLC47A2* | -3,26 | solute carrier family 47, member 2 |
| **Metabolism** | | | | |
| *Lipid metabolism* | | | | |
|  | NM_003956 | CH25H | 2,35 | cholesterol 25-hydroxylase |
|  | NM_000351 | STS | -2,56 | steroid sulfatase (microsomal), isozyme S |
|  | NM_021187 | CYP4F11 | 2,99 | cytochrome P450, family 4, subfamily F, polypeptide 11 |
|  | NM_032717 | AGPAT9 | 2,06 | 1-acylglycerol-3-phosphate O-acyltransferase 9 |
|  | NM_152310 | ELOVL3 | -2,17 | elongation of very long chain fatty acids (FEN1/Elo2, SUR4/Elo3, yeast)-like 3 |
|  | NM_007238 | PXMP4 | -2,72 | peroxisomal membrane protein 4, 24kDa |
| *Nucleotide metabolism* | | | | |
|  | NM_003364 | UPP1 | 2,36 | uridine phosphorylase 1 |
| *Energy metabolism* | | | | |
|  | NM_001482 | GATM | -2,06 | glycine amidinotransferase (L-arginine:glycine amidinotransferase) |
| *Protein metabolism* | | | | |
|  | NM_019891 | ERO1LB | 2,05 | ERO1-like beta (S, cerevisiae) |
| Vitamin D metabolism | | | | |
|  | NM_000782 | CYP24A1 | 2,40 | cytochrome P450, family 24, subfamily A, polypeptide 1 |
| **Ion/ amino acid/ iron Transport** | | | | |
|  | NM_018593 | SLC16A10 | -3,97 | solute carrier family 16, member 10 (aromatic amino acid transporter) |
|  | NM_014585 | SLC40A1 | -3,75 | solute carrier family 40 (iron-regulated transporter), member 1 |
|  | NM_152908 | SLC47A2* | -3,26 | solute carrier family 47, member 2 |
|  | NM_182767 | SLC6A15 | 2,14 | solute carrier family 6 (neutral amino acid transporter), member 15 |
|  | NM_014331 | SLC7A11* | 2,49 | solute carrier family 7, (cationic amino acid transporter, y+ system) member 11 |
|  | NM_003046 | SLC7A2* | 2,92 | solute carrier family 7 (cationic amino acid transporter, y+ system), member 2 |
|  | NM_024524 | ATP13A3 | 2,08 | ATPase type 13A3 |
|  | NM_000891 | KCNJ2 | -2,39 | potassium inwardly-rectifying channel, subfamily J, member 2 |
| **Extracellular Matrix** | | | | |
|  | NM_007350 | PHLDA1 | 2,15 | pleckstrin homology-like domain, family A, member 1 |
|  | NM_014822 | SEC24D | 2,43 | SEC24 family, member D (S, cerevisiae) |
|  | NM_080474 | SERPINB12 | -3,52 | serpin peptidase inhibitor, clade B (ovalbumin), member 12 |
| *TGFpathway* |  |  |  |  |
|  | NM_015277 | NEDD4L | 2,12 | neural precursor cell expressed, developmentally down-regulated 4-like |
|  | NM_001200 | BMP2* | 2,62 | bone morphogenetic protein 2 |
|  | NM_001343 | DAB2 | 2,57 | disabled homolog 2, mitogen-responsive phosphoprotein (Drosophila) |
|  | NM_004864 | GDF15* | 2,99 | growth differentiation factor 15 |
| **Intracellular signalling** | | | | |
|  | NM_001010000 | ARHGAP28 | -2,06 | Rho GTPase activating protein 28 |
|  | NM_005261 | GEM | 2,23 | GTP binding protein overexpressed in skeletal muscle |
|  | NM_004570 | PIK3C2G | -2,02 | phosphoinositide-3-kinase, class 2, gamma polypeptide |
|  | NM_007207 | DUSP10* | 2,23 | dual specificity phosphatase 10 |
| **Transmembrane protein** | | | | |
|  | NM_138461 | TM4SF19 | 5,36 | transmembrane 4 L six family member 19 |
|  | NM_013390 | TMEM2 | 2,00 | transmembrane protein 2 |
|  | NM_015993 | PLLP | -2,06 | plasma membrane proteolipid (plasmolipin) |
| **Proteasome** | | | | |
|  | AK303463 | USP41 | -2,40 | ubiquitin specific peptidase 41 |
|  | NM_014363 | SACS | 2,18 | spastic ataxia of Charlevoix-Saguenay (sacsin) |
|  | NM_003900 | SQSTM1* | 2,22 | sequestosome 1 |
| **Protein traffic** | | | | |
|  | NM_001503 | GPLD1 | -2,26 | glycosylphosphatidylinositol specific phospholipase D1 |
|  | NM_003764 | STX11* | 3,00 | syntaxin 11 |
| **Cytoskeleton** | | | | |
|  | NM_022041 | GAN | -2,02 | gigaxonin |
|  | NM_005909 | MAP1B | 2,06 | microtubule-associated protein 1B |
| **Pigmentation** | | | | |
|  | NM_198159 | MITF | -2,06 | microphthalmia-associated transcription factor |
|  | NM_014331 | SLC7A11* | 2,49 | solute carrier family 7, (cationic amino acid transporter, y+ system) member 11 |
| **Regulation of Gene expression/Chromatin remodeling** | | | | |
|  | NM_005325 | HIST1H1A | -2,00 | histone cluster 1, H1a |
|  | NM_017631 | DDX60 | -2,07 | DEAD (Asp-Glu-Ala-Asp) box polypeptide 60 |
| **Stress** |  |  |  |  |
|  | NM_001040619 | ATF3 | 2,09 | activating transcription factor 3 |
| **Other** |  |  |  |  |
|  | NM_005103 | FEZ1 | 2,17 | fasciculation and elongation protein zeta 1 (zygin I) |
|  | NM_017671 | FERMT1 | 2,02 | fermitin family homolog 1 (Drosophila) |
|  | NM_014570 | ARFGAP3 | 2,04 | ADP-ribosylation factor GTPase activating protein 3 |
|  | NM_005733 | KIF20A | -2,15 | kinesin family member 20A |
|  | NM_021724 | NR1D1 | 2,53 | nuclear receptor subfamily 1, group D, member 1 |
|  | NM_138573 | NRG4 | -2,40 | neuregulin 4 |
|  | NM_024165 | PHF1 | 2,09 | PHD finger protein 1 |
|  | NM_024769 | ASAM | 2,36 | adipocyte-specific adhesion molecule |
|  | NM_181533 | ABHD12B | -2,83 | abhydrolase domain containing 12B |
|  | NM_206966 | C5orf46 | -2,15 | chromosome 5 open reading frame 46 |
|  | NM_183373 | C6orf145 | 2,05 | chromosome 6 open reading frame 145 |
|  | NM_018325 | C9orf72 | 2,19 | chromosome 9 open reading frame 72 |
|  | NM_001215 | CA6 | -2,08 | carbonic anhydrase VI |
|  | NM_014157 | CCDC113 | -2,29 | coiled-coil domain containing 113 |
|  | NM_024519 | FAM65A | 2,01 | family with sequence similarity 65, member A |
|  | NM_017938 | FAM70A | -3,67 | family with sequence similarity 70, member A |
|  | NM_173815 | FLJ37464 | -2,64 | hypothetical protein FLJ37464 |
|  | NM_001490 | GCNT1 | -2,02 | glucosaminyl (N-acetyl) transferase 1, core 2 (beta-1,6-N-acetylglucosaminyltransferase) |
|  | NM_015187 | KIAA0746 | 2,23 | KIAA0746 protein |
|  | NM_005779 | LHFPL2 | 2,02 | lipoma HMGIC fusion partner-like 2 |
|  | BC107865 | LOC204010 | 2,07 | ribosomal protein SA pseudogene |
|  | NM_006152 | LRMP | -2,35 | lymphoid-restricted membrane protein |
|  | NM_024717 | MCTP1 | 2,68 | multiple C2 domains, transmembrane 1 |
|  | NM_014033 | METTL7A | -2,41 | methyltransferase like 7A |
|  | BC066301 | MGC87042 | 2,02 | similar to Six transmembrane epithelial antigen of prostate |
|  | NM_144599 | NIPA1 | 2,11 | non imprinted in Prader-Willi/Angelman syndrome 1 |
|  | NM_001085382 | PSAPL1 | -2,18 | prosaposin-like 1 |
|  | NM_031469 | SH3BGRL2 | -2,02 | SH3 domain binding glutamic acid-rich protein like 2 |
|  | NM_020808 | SIPA1L2 | 2,06 | signal-induced proliferation-associated 1 like 2 |
|  | NM_007000 | UPK1A | -2,09 | uroplakin 1A |
